# Supplementary material for: Agreement between patient’s description of abdominal symptoms of possible upper gastrointestinal cancer and general practitioner consultation notes: a qualitative analysis of video-recorded UK primary care consultation data
Source: BMJ Open. 2023 Jan 5;13(1):e058766. doi: 10.1136/bmjopen-2021-058766 (PMC9827246; doi:10.1136/bmjopen-2021-058766)
Supplement: Supplementary data [file bmjopen-2021-058766supp001.pdf]

Supplementary file A. Details of included patients and their upper GI/systemic bodily sensations

Patient ID	Gender	Age	Bodily sensations
1	Female	66	Follow-up post-emergency operation for strangulated hernia, 'sticky bowels'
2	Female	71	Nausea, acid reflux
3	Female	72	Abdominal and pelvic pain, back ache
4	Male	55	Abdominal pain
5	Female	65	Nausea
6	Female	79	Heartburn, nausea
7	Female	73	Abdominal pain
8	Male	65	Abdominal and rectal pain, loose stools
9	Male	60	Acid reflux, indigestion
10	Male	59	Tiredness, headaches
11	Female	69	Abdominal distension/bloated
12	Male	73	Bowel pain, constipation
13	Male	83	Swallowing difficulties
14	Female	58	Back pain
15	Female	45	Fatigue, difficulty urinating
16	Male	81	Constipation
17	Female	77	Epigastric and back pain, nausea and vomiting
18	Female	53	Abdominal pain, nausea, diarrhoea, blood in stools
19	Female	42	Heartburn
20	Female	72	Reflux
21	Male	71	Loose stools, colitis flare-up
22	Female	41	Change in bowels, low haemoglobin
23	Female	70	Abdominal pain, change in bowels
24	Male	43	Fatigue, change in bowels
25	Female	59	Voice hoarseness, stomach problems
26	Male	74	Hernia
27	Male	64	Gastritis, follow-up of biopsy reversal
28	Female	53	Epigastric discomfort
