# Supplementary material for: Agreement between patient’s description of abdominal symptoms of possible upper gastrointestinal cancer and general practitioner consultation notes: a qualitative analysis of video-recorded UK primary care consultation data
Source: BMJ Open. 2023 Jan 5;13(1):e058766. doi: 10.1136/bmjopen-2021-058766 (PMC9827246; doi:10.1136/bmjopen-2021-058766)
Supplement: Author's manuscript [file bmjopen-2021-058766.draft_revisions.pdf]

BMJ Open

BMJ Open is committed to open peer review. As part of this commitment we make the peer review history of every article we publish publicly available.

When an article is published we post the peer reviewers' comments and the authors' responses online. We also post the versions of the paper that were used during peer review. These are the versions that the peer review comments apply to.

The versions of the paper that follow are the versions that were submitted during the peer review process. They are not the versions of record or the final published versions. They should not be cited or distributed as the published version of this manuscript.

BMJ Open is an open access journal and the full, final, typeset and author-corrected version of record of the manuscript is available on our site with no access controls, subscription charges or pay-per-view fees (http://bmjopen.bmj.com).

If you have any questions on BMJ Open's open peer review process please email info.bmjopen@bmj.com

Agreement between patient's description of abdominal symptoms of possible upper gastrointestinal cancer and General Practitioner consultation notes: a qualitative analysis of video-recorded UK primary care consultation data

Journal:	*BMJ Open*
Manuscript ID	bmjopen-2021-058766
Article Type:	Original research
Date Submitted by the Author:	29-Oct-2021

Complete List of Authors:	Hardy, Victoria; University of Cambridge, Public Health and Primary Care<br>Usher-Smith, Juliet; University of Cambridge, Department of Public Health and Primary Care<br>Archer, Stephanie; University of Cambridge, Department of Public Health and Primary Care<br>Barnes, Rebecca ; University of Oxford Nuffield Department of Primary Care Health Sciences<br>Lancaster, John; University of Cambridge, Patient Representative c/o Public Health & Primary Care<br>Johnson, Margaret ; University of Cambridge, Patient Representative c/o Public Health & Primary Care<br>Thompson, Matthew; University of Washington, Department of Family Medicine<br>Emery, Jon; University of Melbourne Victorian Comprehensive Cancer Centre, Centre for Cancer Research and General Practice and Primary Care Academic Centre<br>Singh, Hardeep; Michael E DeBakey VA Medical Center, Houston Veterans Affairs Center for Innovations in Quality, Effectiveness and Safety; Baylor College of Medicine, Department of Medicine<br>Walter, Fiona; Queen Mary University of London, Wolfson Institute of Population Health, Barts and the London School of Medicine and Dentistry; University of Cambridge, Department of Public Health and Primary Care

Keywords:	PRIMARY CARE, QUALITATIVE RESEARCH, MEDICAL EDUCATION & TRAINING

SCHOLARONE™
Manuscripts

I, the Submitting Author has the right to grant and does grant on behalf of all authors of the Work (as defined in the below author licence), an exclusive licence and/or a non-exclusive licence for contributions from authors who are: i) UK Crown employees; ii) where BMJ has agreed a CC-BY licence shall apply, and/or iii) in accordance with the terms applicable for US Federal Government officers or employees acting as part of their official duties; on a worldwide, perpetual, irrevocable, royalty-free basis to BMJ Publishing Group Ltd ("BMJ") its licensees and where the relevant Journal is co-owned by BMJ to the co-owners of the Journal, to publish the Work in this journal and any other BMJ products and to exploit all rights, as set out in our licence.

The Submitting Author accepts and understands that any supply made under these terms is made by BMJ to the Submitting Author unless you are acting as an employee on behalf of your employer or a postgraduate student of an affiliated institution which is paying any applicable article publishing charge ("APC") for Open Access articles. Where the Submitting Author wishes to make the Work available on an Open Access basis (and intends to pay the relevant APC), the terms of reuse of such Open Access shall be governed by a Creative Commons licence – details of these licences and which Creative Commons licence will apply to this Work are set out in our licence referred to above.

Other than as permitted in any relevant BMJ Author's Self Archiving Policies, I confirm this Work has not been accepted for publication elsewhere, is not being considered for publication elsewhere and does not duplicate material already published. I confirm all authors consent to publication of this Work and authorise the granting of this licence.

For peer review only

Agreement between patient's description of abdominal symptoms of possible upper gastrointestinal cancer and General Practitioner consultation notes: a qualitative analysis of video-recorded UK primary care consultation data

Victoria Hardy[1], Juliet Usher-Smith[1], Stephanie Archer[1], Rebecca K. Barnes[2], John Lancaster[1], Margaret Johnson[1], Matthew Thompson[3], Jon Emery[4], Hardeep Singh[5], Fiona M. Walter[6,1]

Corresponding author

Victoria Hardy, Department of Public Health & Primary Care, University of Cambridge, Cambridge, UK

Veh29@medschl.cam.ac.uk

Affiliations

[1]Department of Public Health & Primary Care, University of Cambridge, Cambridge, UK

[2]Nuffield Department of Primary Care Health Sciences, University of Oxford, Oxford, UK

[3]Department of Family Medicine, University of Washington, Seattle, Washington, USA

[4]Centre for Cancer Research and Department of General Practice, University of Melbourne, Victorian Comprehensive Cancer Centre, Melbourne, Victoria, Australia

[5]Center for Innovations in Quality, Effectiveness and Safety, Michael E. DeBakey VA Medical Center and Baylor College of Medicine, Houston, TX, USA.

[6]Wolfson Institute of Population Health, Barts and the London School of Medicine and Dentistry, Queen Mary University of London, UK

Key words: primary care, symptoms, diagnosis, qualitative research

Word count: 4398

For peer review only - http://bmjopen.bmj.com/site/about/guidelines.xhtml

Abstract

Introduction

Abdominal symptoms are common in primary care but infrequently might be due to an upper gastrointestinal (UGI) cancer. Patients' descriptions may differ from medical terminology used by General Practitioners (GPs). This may affect how information about abdominal symptoms possibly due to an UGI cancer are documented, creating potential missed opportunities for timely investigation.

Objectives

To explore how abdominal symptoms are communicated during primary care consultations, and identify characteristics of patients' descriptions that underpin variation in the accuracy and completeness with which they are documented in medical records.

Methods and analysis

Primary care consultation video recordings, transcripts and medical records from an existing dataset were screened for adults reporting abdominal symptoms. We conducted a qualitative content analysis to capture alignments (medical record entries matching patient verbal and non-verbal descriptions) and misalignments (symptom information omitted or differing from patient descriptions). Categories were informed by the Calgary-Cambridge Guide's 'gathering information' domains and patterns in descriptions explored.

Results

Our sample included 28 consultations (28 patients with 18 GPs): ten categories of different clinical features of abdominal symptoms were discussed. The information GPs documented about these features commonly did not match what patients described, with misalignments more common than alignments (71 vs 40 instances, respectively). Misalignments often featured patients using vague descriptors, figurative speech, lengthy explanations and broad hand gestures. Alignments were characterised by patients using well-defined terms, succinct descriptions and precise gestures for symptoms with an exact location. Abdominal sensations reported as 'pain' were almost always documented compared to expressions of 'discomfort'.

Conclusions

Abdominal symptoms that are well-defined or communicated as 'pain' may be more salient to GPs than those expressed vaguely or as 'discomfort'. Variable documentation of abdominal symptoms in medical records may have implications for the development of clinical decision support systems and decisions to investigate possible UGI cancer.

For peer review only - http://bmjopen.bmj.com/site/about/guidelines.xhtml

ARTICLE SUMMARY

Strengths and limitations

- This is the first study to explore similarities and differences between how patients describe and GPs document abdominal symptoms that could be due to an UGI cancer using naturalistic UK primary care consultation data
- We did not have access to patients' previous consultation history and so could not determine the clinical importance of symptom information that was either not documented in medical records, or did not reflect the patient's history.
- As our sample was mostly White British and native English speakers, patterns in how patients described and GPs documented abdominal symptoms may not be transferable to practices serving patient populations with different characteristics

For peer review only

Introduction

Sir William Osler, lauded as the father of modern medicine, implored his medical students to *"listen to your patient: he is telling you the diagnosis"*.(1) Over a century later, this adage remains germane. As depicted in the National Academy of Medicine's (NAM) conceptual model of the diagnostic process, an accurate and timely diagnosis is predicated on clinicians collecting sufficient data from the patient history.(2) Communication skills teaching is integral to medical training,(3) but suboptimal information gathering is common during the medical interview(4–6) and has been implicated in diagnostic error in primary care settings.(5)

Abdominal symptoms are common in patients presenting to primary care.(7) While such symptoms far more frequently herald benign conditions,(8) they are also described by patients diagnosed with upper gastrointestinal (UGI) cancers (i.e., gastric, oesophageal, and pancreatic), as well as other cancers within the abdomen.(7) In the United Kingdom (UK), compared with 'alarm' symptoms which have relatively high positive predictive value (PPV), abdominal symptoms alone do not meet the 3% PPV threshold recommended by the National Institute for Clinical Excellence (NICE) NG12 (2015) for a 'fast-track' suspected cancer referral.(9) Compounded by a lack of screening programmes or biomarkers reliably raising suspicion of UGI cancer in low prevalence populations,(10) patients with UGI cancers are disproportionately burdened with lengthy diagnostic intervals and late-stage diagnosis, associated with worse survival and patient experiences of healthcare.(11) While the novel 'CytoSponge' test (for Barrett's oesophagus, a precursor lesion for oesophageal cancer(12)) is a promising development for early detection, effective triage of abdominal symptoms for a timely UGI cancer diagnosis, remains currently largely contingent upon the patient history.

Symptoms are subjectively experienced changes in bodily sensations that reflect a perceived departure from the body's "normal" state.(13,14) Patients' experiences of bodily changes are the convergence of sociocultural(13) and experiential factors,(14) and may be communicated differently from standardised medical terminology used by general practitioners (GPs).(15) Studies of UGI cancer patients' symptom appraisal have shown they use a wide range of words and phrases to conceptualise abdominal changes(16,17) that are inconsistent with medical definitions familiar to GPs.(17,18) Without a common language, potentially significant bodily sensations risk being overlooked or misinterpreted, and the clinical picture distorted as 'lay' descriptions are translated into equivalent medical nomenclature.(15) Consequently, what GPs document in medical records may not be a complete or accurate representation of the patient's history,(19) potentially creating opportunities for diagnostic delay.

As well as being important at the patient-level for subsequent management decisions, the ways GPs document patients' bodily sensations in medical records is important for population approaches to early cancer detection. Initiatives in the UK currently harvest coded symptom data from primary care records to develop national guidelines and prediction tools that help

For peer review only - http://bmjopen.bmj.com/site/about/guidelines.xhtml

GPs estimate patients' current or future cancer risk.(20–23) However, there is mounting interest in harnessing Natural Language Processing techniques to incorporate free-text symptoms.(24,25) Determining how patients describe abdominal sensations to GPs and the extent to which medical records reflect the patient's history is paramount for ensuring clinical decisions tools for UGI (and other) cancers facilitate effective use of tests and referral pathways.

To address this research need, we compared video-recorded primary care consultations featuring new or ongoing abdominal sensations that could be due to an UGI cancer with medical record documentation and explored similarities and differences between patient descriptions of bodily sensations and the way GPs document these as symptoms. We aimed to explore how abdominal symptoms are recorded in the medical record to identify the characteristics of patients' descriptions underpinning GP variation in the medical record entries and the implications of this on opportunities for earlier diagnosis of UGI cancers. This study builds on research from the wider literature that has utilised naturalistic data to quantify the concordance between GPs' spoken and documented safety-netting advice,(26) and information captured in medical records by physicians compared to details given by unannounced standardised patients (USPs).(27)

Methods

Design

A cross-sectional descriptive study of video-recorded primary care consultations with linked medical record data entries from the *"One in a Million"* primary care consultation archive, analyzed using qualitative content analysis.

Data source

The *"One in a Million"* dataset was derived from the 'Bristol Archive project,' a prospective observational cohort study that sought to create a repository of routine primary care consultation data available for use in future research.(28) Between July 2014 and April 2015, twelve purposively sampled general practices in the West of England recorded unselected consultations with adult patients (aged 18 years and above) who presented to primary care over two to three half days.(29) A total of 327 consultations were recorded with linked patient, GP and practice demographic data, and available medical record entries. 89.8% (n=300) of patients consented to future data access by other researchers, subject to specific ethical approval. All these recordings were anonymized for spoken identifiers, transcribed verbatim and coded for their content according to sixteen "problem type(s)" based on the International Classification of Primary Care – 2nd Edition (ICPC-2) classification scheme.(29) Consultations were assigned more than one code if multiple problems were discussed. We requested all available data for 72 consultations archived as 'digestive' or 'general and unspecified'(29) to yield data directly relevant for addressing our research question.

For peer review only - http://bmjopen.bmj.com/site/about/guidelines.xhtml

Eligibility criteria and assessment

We included consultations if there was: a) an accompanying videorecording, transcript, and medical record entry, and b) communication of any new or ongoing abdominal and systemic sensations that could reflect an underlying UGI cancer. We limited inclusion to patients aged 40 years and above, because UGI cancers below this age are rare.(30) Consultations of patients known to have a previous or current cancer diagnosis based on information disclosed during the video-recording were excluded, as a history of cancer provokes more suspicion of serious illness among GPs and patients, and we were interested in GPs' appraisal of patient's bodily sensations in an as-yet undiagnosed population. We also excluded consultations during which no relevant bodily sensations were communicated. This was determined by abstracting all bodily sensations discussed during consultations into a pre-prepared Microsoft Excel form. Bodily sensations were then assessed by VH (a non-clinical PhD student) as 'include', 'exclude', or 'unsure', before final adjudication of relevant bodily sensations by FW (a senior academic GP).

Sampling

We used a convenience sampling strategy whereby all consultations of symptomatic patients that met study eligibility criteria were included.

Data analysis

Data were analysed using qualitative content analysis, which is suitable for exploring and describing similarities and differences in the content of textual and visual data sources.(31–33) Data analysis was conducted in excel by VH, with analysis facilitated by SA (senior non-clinical researcher), and data interpretation verified by FW, JUS (senior academic GPs), and PPI (JL, MJ).

We employed a mixed deductive and inductive approach whereby the analysis was informed by relevant clinical features specified across the 'gathering information' domains of the Calgary-Cambridge Guide: biomedical perspective, patient perspective, and background information. The Calgary-Cambridge Guide is an evidence-based communication tool used in medical training programmes internationally to promote consultation quality by encouraging the collection and integration of information pertaining to both the patient and biomedical perspectives. Data analysis followed four phases of content analysis outlined by Erlingsson *et al.*: 'familiarising oneself with the data', 'dividing and condensing the text into meaning units', 'formulating codes', and 'developing categories and themes'.(34)

After transcripts had been cross-referenced with video-recordings to verify accuracy, transcripts, video-recordings, and medical record entries were reviewed repeatedly, individually and concomitantly, to become familiar with the content. Video-recordings were reviewed alongside transcripts to observe the nonverbal body language exhibited by patients and GPs. Six nonverbal aspects of communication were annotated in parentheses adjacent to the relevant text. These included posture, proximity, touch, body movements, facial expression, and eye behaviour.(35)

For peer review only - http://bmjopen.bmj.com/site/about/guidelines.xhtml

Short verbatim excerpts or 'meaning units' (encompassing nonverbal gestures) were juxtaposed with medical record entries using an adapted consultation proforma originally developed for examining the content of different primary care data sources.(36) Throughout analysis, excerpts from each consultation were kept together to allow contextualization of the data. 'Meaning units' were condensed by VH into an abbreviated narrative capturing the essence of patient's reason for presentation. Care was taken to preserve the patient voice by weaving verbatim descriptions from excerpts into a reconfigured narrative. Patient reported bodily sensations were then compared with symptoms documented by GPs in the patients' medical record, and alignments and misalignments in documentation coded. Alignments were symptom information in medical records that both mirrored the content and verbal and non-verbal descriptors patients communicated; misalignments were symptoms that had either not been documented or differed from information given by patients. This task was independently undertaken by VH and two patient and public representatives (PPI) (MJ and JL); differences were resolved through further discussion, and consensus with FW and JUS. Coded excerpts constituted the unit of analysis and encompassed multiple features of an individual symptom.

Coded alignments and misalignments were separated from original data sources and, with regular input from study authors, iteratively grouped into descriptive categories, informed by the symptom features listed in the 'gathering information' domains.(35)

Final categories were re-contextualised, and the features of patient's verbal descriptions and non-verbal gestures underpinning alignments and misalignments explored. Attention was given to isolated and discrepant excerpts to illuminate as much diversity of symptom expression encountered in general practice. We also explored differences in GP documentation by GP and patient demographic characteristics.

Ethical considerations

Research ethics approval was obtained from the East of Scotland Research Ethics Committee (REC) prior to commencing the study (reference: 19/ES/0057).

Patient and public involvement

Two patient and public involvement representatives (JL, ML) actively advised the research group from study inception to manuscript preparation. Both representatives were involved in the analysis and interpretation of the data, and will facilitate dissemination of study findings.

For peer review only - http://bmjopen.bmj.com/site/about/guidelines.xhtml

Results

Included consultations

Of the 72 consultations requested from the 'One in a Million' data repository, 28 met study eligibility criteria and were included in the final analysis (**Figure 1**). Participants comprised 28 patients, one caregiver, and 18 GPs. Overall, patients presented with 46 new or ongoing problems, captured in a total of 111 textual excerpts that were relevant for this analysis.

INSERT: Figure 1. Flow diagram of included consultations

Sample characteristics

Characteristics of included patients and GPs are presented in **Table 1** (the one caregiver's characteristics were unavailable)**.** Patients were mostly female (*n*=17), aged 40 to 89 years, with varying educational attainment. GPs were aged 30 to 69 years and had been qualified as a GP for up to 31 years. Patients and GPs were all born in the UK and spoke English as their primary language. Apart from one patient of mixed ethnicity, our sample was white British. Patients presented with a variety of UGI and systemic bodily sensations (**Supplementary File A)**.

Table 1. Sample characteristics

For peer review only - http://bmjopen.bmj.com/site/about/guidelines.xhtml

Patient characteristics	Number of patients (*N*=28)	GP characteristics	Number of GPs (*N*=18)
**Sex**		**Sex**	
Male	11	Male	10
Female	17	Female	8
**Ethnicity***		**Ethnicity**	
White British/White other	26	White British/White other	18
Mixed/multiple ethnic groups	1	Mixed/multiple ethnic groups	-
**Age**		**Age**	
30-39	NA	30-39	5
40-49	5	40-49	5
50-59	6	50-59	7
60-69	6	60-69	1
70-79	9	70-79	-
80-89	2	80-89	-
**Main language***		**Main language**	
English	27	English	18
**Country of birth**		**Country of birth**	
UK	28	UK	18
**Highest educational attainment**		**Years since qualified as a GP**	
Degree or higher degree	8	1-10	5
A/AS-levels or advanced diploma	2	11-20	6
Professional or vocational qualifications	6	21-30	6
Apprenticeship	3	31+	1
No qualifications	4		
**IMD quintiles**			
1st (least deprived)	6		
2nd	8		
3rd	4		
4th	5		
5th (most deprived)	5		

*Not reported (*n*=1); IMD, index of multiple deprivation; NA, not applicable (not eligible for inclusion)

GP alignments and misalignments

Overall, there was more evidence of GP misalignments (*n*=71) than alignments (*n*=40), with a median of 2 (*range:* 0–8) misalignments and 1 (*range:* 0–5) alignments, per consultation.

We identified ten categories reflecting ten clinical features which were informed by the 'gathering information' domains of the Calgary-Cambridge Guide (**Figure 2** and **Table 2**).(35) Of these ten categories/features, seven related to the *'biomedical perspective',* and three to the *'patient's perspective' (***Figure 2***)*. Most misalignments occurred for abdominal sensations that were 'persistent or evolving', or when documenting the 'location', 'effect of on patients' life', and 'self-management' of abdominal sensations (**Figure 3**). Alignments were most common when abdominal sensations were described as 'pain' compared to 'discomfort', and when capturing patient's ideas and beliefs. Findings for all ten clinical features are detailed below. A summary of the main differences in patient descriptions of each feature between alignments and misalignments is presented in **Table 2**.

For peer review only - http://bmjopen.bmj.com/site/about/guidelines.xhtml

INSERT: Figure 2. Final categories representing the clinical features of abdominal sensations discussed in primary care consultations based on the 'gathering information' domains of the Calgary-Cambridge model

INSERT: Figure 3. Frequency of alignments and misalignments for each feature of patients' abdominal and systemic bodily sensations, according to the 'gathering information' domain of the Calgary-Cambridge Guide

Table 2. Differences in the characteristics of patients' verbal and non-verbal descriptions of abdominal sensations for alignments and misalignments

Clinical feature	Alignments	Misalignments
**Domain: Biomedical perspective**		
Persistent or evolving	Well-defined terms, articulating changes in quality, frequency, and intensity of worsening sensations	Non-definitive, figurative speech for bodily sensations that were worsening, improving but not resolved, or re-appraised as worsening
Location of pain/discomfort	Well-defined or medical terms, succinctly communicated; non-verbal gestures pointing to site of pain/discomfort	Difficulty articulating precise site of pain/discomfort; broad or global non-verbal gestures not indicating site of pain/discomfort
Aggravating and relieving factors	Communication of a relationship between activities of daily living and onset or worsening of sensations	Infrequent provocation of sensations; lengthy explanations regarding activities that provoked or worsened sensations; activities that relieved sensations
Discomfort	Adjectives or figurative speech for unpleasant sensations	Adjectives or figurative speech for unpleasant sensations
Pain	Pain explicitly articulated with qualifying adjectives, similes, and verbs describing an aversive sensation	Pain explicitly articulated with qualifying adjectives, similes, and verbs describing an aversive sensation
Duration	Communicating the day or number of weeks or months since onset of symptoms	Vague or oblique sentences without offering a timeframe
Self- management	Reporting sensations that were controlled or settling with medication	Reporting suboptimal therapeutic effect of medication
**Domain: Patient's perspective**		
Effects on life	*(no differences in patient descriptions between alignments and misalignments)*	
Ideas and beliefs	Plausible and convincing explanation as to underlying cause related to medication side-effects, flare-up of pre-existing condition, diet, or stress	Lack of a justified explanation for possible cause of sensations
Concerns about what symptoms might mean	Explicit concerns voiced about the possibility of cancer, or more broad concerns that something was not right	Verbalising concerns about the possibility symptoms had a sinister cause, but not explicitly mentioning cancer

Domain: Biomedical perspective

Persistent or evolving

For peer review only - http://bmjopen.bmj.com/site/about/guidelines.xhtml

Abdominal sensations that were persistent or evolving in frequency or intensity were described by patients most often, though were poorly reflected in medical records. Alignments occurred when patients used well-defined or concrete terms to indicate that their bodily sensations were progressing:

Patient: *"'my poo has got a lot freer and a lot looser probably in about the last six weeks. Most of the time when I go to the loo I will do some sort of poo as well at the same time. (shakes head)" (P22: F, 40-49 years)*

Medical records: last *6 weeks loose stools and more freq (GP19)*

Abdominal sensations described in non-definitive ("not getting better") or figurative speech, that were receding but not resolved, or that patients re-appraised as worsening, were characteristic of misalignments. In these instances, bodily sensations were erroneously documented as normal, resolving, or stabilised, respectively:

Patient: *"bowels are a bit sticky (intermittent eye contact, squeezing toothpaste motion with both hands) but I've been eating prunes and apricots and (laughs)*

GP: *"And do you tend to have problems in that area anyway?"*

Patient: *"Not normally (looks upwards) and that was part of the symptoms last week" (P1: F, 60-69)*

Medical records: *constipation improving with prunes. (GP23)*

Location of pain/discomfort

The location of patients' problem was discussed in relation to abdominal 'pain' or 'discomfort' on thirteen occasions. Misalignments were slightly more common than alignments. They occurred when pain/discomfort was communicated to be diffuse, move around, radiate to another location, or in instances when patients were unable to articulate the precise location and relied on large non-verbal hand gestures.

GP: *"So where are you point- (tilts head) is it the middle of the belly, upper belly?"*

Patient: *"I'm not really sure, (looking down at abdomen) but I'm just going like that with it, (rocking back and forth both arms cradling abdomen) because it…" (P18: F, 50-59 years)*

Medical records: *nothing recorded in relation to location (GP15)*

Location of abdominal pain/discomfort was accurately documented when a specific anatomical site was isolated, communicated in succinct and well-defined (sometimes medical) terms, and supplemented with non-verbal gestures that reinforced the specific site of their pain/discomfort:

For peer review only - http://bmjopen.bmj.com/site/about/guidelines.xhtml

Patient: *"I've had an abdominal discomfort for about four or five weeks. It's just sore, really, just below the sternum. (tips of fingers press into epigastric region). (P28: F, 50-59 years)*

Medical records: *epigastric pain (GP12)*

Aggravating and relieving factors

Aggravating and relieving factors referred to activities of daily living that provoked, aggravated, or worsened patients' bodily sensations. GPs' medical record entries accurately reflected information patients communicated in relation to this feature, when a causal relationship between an activity (i.e., stress, medication, and certain foods) and the onset or worsening of sensations was articulated:

Patient: *"It's like cucumber, red onion, tomatoes; it's certain things. I love to eat jacket potatoes, it just sets off heartburn. (P19: F, 40-49 years)*

Medical records: *Gastro-oesophageal reflux: with certain foods (GP18)*

Information about activities aggravating bodily sensations were omitted from medical records when they were described as infrequent or improving, gave lengthy explanations of the precipitating factors, or in one instance, communicated an activity that eased bodily sensations:

Patient: *"I have weeks now as long as I don't eat sugar and I don't have any stress it's often really quite good […] At the moment it's a bit worse because I'm fighting for my son's funding and that gets me stressed. (shifts in chair, strokes forehead) Mostly it's so much better." (P25: F, 50-59 years)*

Medical records: *nothing recorded in relation to aggravating and relieving factors (GP20)*

Discomfort

Though there were no differences in patients' communication of discomfort between alignments and misalignments, patients used a range of adjectives and similes to describe abdominal sensations that were causing discomfort. Subtle verbal descriptors, such as "a nuisance" or as though "something was pressing down" conveyed discomfort as a sensation that was interfering or unpleasant. These descriptions were omitted or inaccurately reflected by GPs in most instances:

Patient: *"I can still feel a little something there (left hand touches hollow of neck) […] a sensation up here but it can't be a physical blockage because they looked […] it's just I feel like you've got an airlock..." (P20: F, 70-79 years)*

Medical records: *nothing recorded in relation to the quality of the sensation (GP18)*

Pain

For peer review only - http://bmjopen.bmj.com/site/about/guidelines.xhtml

GPs almost always documented 'pain' when it was mentioned. There were no observable differences in how patients described pain between alignments and the two misalignments. Patients also qualified 'pain' with supplementary adjectives, verbs, and similes, such as "terrible", "stabbing", and "like waves, like contractions", that communicated an aversive sensation. Such qualifiers were characteristic of both alignments and misalignments and rarely captured in medical records.

Duration

The duration of patients' bodily sensations was infrequently described. GPs' accurately documented what patients communicated in relation to this feature in half of the eight instances it was discussed. Offering a discrete timeframe by giving the day of the week or number of weeks or months that had elapsed since the onset of bodily sensations, was equally characteristic of misalignments and alignments:

**GP:** *"So how long have you been poorly, now?"*<br>**Patient:** *"Well, it wasn't last week. What's today? Tuesday. It was the Friday week. Last Friday week, yes" (nods head) (P3: F, 70-79 years)*	**Medical records:** *lower abdo pelvic pain for 10 days (GP5)*

In contrast, vague or open-ended descriptions of the time since onset were slightly more typical of this feature being omitted from medical records.

Self-management

Patients described use of prescription and over-the-counter medication taken to manage their bodily sensations. GP misalignments, which were more commonplace than alignments, were mostly characterised by lengthy explanations that the medication had eased but not resolved, or had failed to appreciably control the severity of bodily sensations:

**Patient:** *"I took those capsules. I took one in the morning before anything else. And I took one at night before a meal […] It [upper abdominal pain] settled a little bit. But it's still there (right hand presses upper abdomen) (P17: F, 70-79 years)*	**Medical records:** nothing *recorded in relation to self-management (GP14)*

In the few instances when GP documentation matched what patients had communicated, patients asserted that the medication was either helping to settle or control bodily sensations, or was having no effect:

**Patient:** *"I started taking prednisolone… [for colitis flare-up]. Well, it is working. (eye contact, nods head) I've been taking them for a couple of weeks and the condition is improved…(P21: M, 70-79 years)*	**Medical records:** *ulcerative colitis settled now with use of oral steroids (GP19)*

Domain: The patient's perspective

For peer review only - http://bmjopen.bmj.com/site/about/guidelines.xhtml

Effect on life

The effect of patients' bodily sensations on functional and social aspects of their life was discussed on twelve occasions but was rarely documented in medical records. There were no clear language differences between misalignments and alignments.

Ideas and beliefs

Patient's ideas and beliefs were documented on most occasions when patients offered a plausible explanation as to what may be causing their bodily sensations. Patients typically attributed sensations to medication side-effects, flare-up of a pre-existing condition, diet, and stress, which on occasion matched GPs ideas and beliefs. In the one instance that patient's ideas and beliefs were omitted from records, the patient was unable to justify the possible cause.

Concern about what symptoms might mean

Patients rarely expressed concerns about the cause or seriousness of their bodily sensations. In the two instances where patient's concerns were documented in medical records, patients had either relayed their partner's concern about "looking drawn" or explicitly asked the GP whether their bodily sensations could be due to an underlying cancer:

**Patient:** *"You don't think it's cancerous basically? (shakes head, right hand holding shirt collar)" (P25: F, 50-59 years)*	**Medical records:** *Very worried about cancer (GP20)*

Patients' concerns were typically not documented when the possibility of a sinister cause was voiced but cancer was not directly articulated:

**Patient:** *"There's nothing bad really down there? (touches upper abdomen with both hands) […] Because it swells up quite a bit when it happened" (P17: F, 70-79 years)*	**Medical records:** *nothing recorded in relation to patient concerns (GP14)*

GP and patient characteristics

The frequency of alignments and misalignments for each clinical feature was insufficient for stratifying data to identify GP characteristics (sex, age, years since qualified as a GP) or patient characteristics (sex, age, educational attainment, social deprivation level) as additional sources of GP variation in how abdominal symptoms were documented.

Discussion

Summary of key findings

For peer review only - http://bmjopen.bmj.com/site/about/guidelines.xhtml

A wide range of clinical features pertaining to abdominal and systemic sensations were discussed between patients and GPs during primary care consultations. There was a gap between what patients communicated and the information about these bodily sensations GPs documented in medical records. Vague descriptors, figurative speech, and lengthy explanations of bodily sensations without an exact location, characterised features of patients' history that was not recorded, documented incompletely, or did not match what patients verbalised. Clinical features that were well-defined and succinct with precise gesticulations for abdominal sensations were well documented. Abdominal 'pain' was almost always documented compared to 'discomfort'. 'Discomfort' was conveyed using descriptors that indicated sensations that were unpleasant, whereas 'pain' was communicated as an aversive sensation. Abdominal features were omitted from medical records when large hand gesticulations, which supplemented or superseded verbal descriptions for the location of 'pain' or 'discomfort', were used.

Strengths and limitations

A strength of this work is that our findings are derived from direct comparison of naturalistic data, verified through video observation and medical records. By leveraging the 'gathering information' domains of an established communication teaching tool in our analysis,(37) we characterised differences in patients' descriptions between GP alignments and misalignments according to the clinical features doctors are trained to explore during the medical interview. Our findings are therefore directly applicable to medical training programs. As abdominal symptoms more often suggest conditions other than cancer, our findings are relevant for enhancing the information GPs gather for differentiating serious and non-serious conditions more broadly. However, findings from this study only reflect the immediately observable aspects of verbal and non-verbal communication, rather than latent meaning. Thus, misalignments are not synonymous with a poor history as GPs may have accurately interpreted patients intended meaning despite superficial differences in written terms.(38) A complete patient history is more than the sum of its parts; parsing abdominal sensations from the patient narrative (as in our approach) potentially negates correct interpretation of the overall clinical picture. As we did not have access to records of patients' prior medical history or final diagnosis, we could not determine whether symptoms documented (or omitted) were truly indicative of serious disease. We therefore identified that there was a difference between what patients communicated and what GPs documented, not whether this difference was clinically important. Our sample was almost exclusively White British and native English speakers so our findings may not be transferable to practices serving patient populations with different characteristics. Data interpretation is subjective and influenced by the epistemic position of the research team; VH is a PhD student with a physiotherapy background where the character of pain implicates the involved structure. Consequently, certain aspects of the patient's history may have been more apparent than would have been if approached through a different lens. To ensure confirmability, data interpretation was

For peer review only - http://bmjopen.bmj.com/site/about/guidelines.xhtml

regularly discussed and revised in collaboration with senior academic GPs (FW, JUS) and PPI (MJ, JL).

Comparison with existing research

This is the only study to directly compare medical record entries with the patient's history for undifferentiated symptoms of possible cancer. Interview studies in the UK have contrasted differences between 'lay' experiences of abdominal symptoms among ovarian and esophagogastric cancer patients with medically recognised terms.(17,18) Patients articulated 'bloating' when describing an increase in abdominal size rather than the medical term 'abdominal distension',(18) or conflated heartburn, reflux, and indigestion (e.g., heartburn, medically defined as a "burning sensation in the retrosternal area",(39) was experienced as burning and acid reflux).(17) A Swedish study of over 300 patients compared abdominal symptoms reported by patients and GPs in a retrospective survey for patients receiving faecal immunochemical testing. GPs more frequently reported symptoms patients had indicated as more severe, with concordance increasing from 69.8% to 85.3% for mild and severe abdominal pain, respectively.(19) As retrospective methods are susceptible to recall bias, they are limited in their ability to capture bodily sensations and descriptors communicated or documented during a consultation. 'Pain' has historically been understood to reflect actual or potential physiological damage whereas 'discomfort' is considered to be an unpleasant feeling, despite both reflecting subjectively experienced states.(40,41) That 'pain' was documented more readily than 'discomfort' suggests GPs may attach greater import to bodily sensations described as 'pain' over 'discomfort'. Existing research highlights that patients' experiences of their bodily sensations differ from medically accepted definitions. We extend extant literature, by illuminating that discrepancies continue into the medical record, and identify characteristics of patients' descriptions which may affect what GPs view as salient and document, with poorly defined and non-specific abdominal symptoms less well captured.

Future research

We recommend future research examines the effect the different characteristics of patient's descriptions of their abdominal sensations identified in this study have on GPs' subsequent use of tests and referral pathways leading to an UGI cancer diagnosis. In particular, GPs interpretation of abdominal sensations described as 'pain' or 'discomfort' and subsequent diagnostic action warrants further enquiry. This may (in part) address gaps in current knowledge regarding additional sources of unaccounted practice variation in the rate and accuracy of suspected cancer referrals.(42,43) Additionally, it will be important to determine if differences between what patients describe and GPs document (or omit) leads to clinically important variation in quality of care metrics for early cancer diagnosis (e.g., number of pre-referral consultations, diagnostic interval length).

Implications for policy and practice

For peer review only - http://bmjopen.bmj.com/site/about/guidelines.xhtml

The intended purpose of medical records is to provide a ledger of "relevant clinical findings"(46,47) which can be affected by comorbidities, competing patient agendas,(48,49) and the structure and relative brevity of medical records in UK general practice.(47) Nonetheless, differences between the information patients communicate about their abdominal sensations and what GPs document is potentially clinically important given the majority of patients with cancer are diagnosed following symptomatic presentation to primary care,(50) and post-hoc case reviews suggest some symptoms are interpreted incorrectly or not acted upon.(51,52) Abdominal sensations described in well-defined terms or as 'pain' may be deemed more significant than those experienced as 'discomfort' or described more vaguely. This has implications for sex and socioeconomic inequalities in the timely investigation of cancer.(30,53–56) Patients more able to present their problems succinctly or in terminology familiar to GPs may be perceived to have more concerning symptoms which are investigated more promptly than patients with serious illness but who are less able to articulate their symptoms. GPs should be cognizant that how patients describe abdominal sensations may not be synonymous with illness severity. Patients' descriptions reflect how those bodily sensations are experienced within a broader sociocultural context. Therefore, there may not be an equivalent medical symptom. Policy makers and guideline developers should avoid false dichotomies that give precedence to abdominal symptoms packaged in 'medical' language, for example 'pain' over 'lay' expressions or 'discomfort'. Correct differentiation and documentation of diagnostically relevant symptoms may be aided by strategies that support GPs to decipher and act on patients' intended meaning rather than clinically exacting terminology. The integrity of current and future UGI risk prediction models(20,21,44,45) incorporating coded or free-text symptoms from medical records may be undermined if consultation notes do not reflect the patient's history. Finally, growing use of 'chatbot' or App technologies which utilize symptoms entered remotely by patients to suggest clinical management may be flawed if information about abdominal symptoms relies solely on medical terminology.(59)

Funding

This work is supported by the CanTest Collaborative which is funded by Cancer Research UK (C8640/A23385), where VH is a PhD student, FMW is Director, and MJT, HS, and JE are Associate Directors. SA is supported by Cancer Research UK grants (C12292/A20861). The Bristol Archive Project was funded by the National Institute for Health Research (NIHR) School for Primary Care Research (208) and the South West GP Trust.

Contributions

VH and FW designed the study. VH led analysis of the data and initial drafting of the manuscript with support from JUS, SA, and FW. All authors critically reviewed and approved the manuscript for submission.

For peer review only - http://bmjopen.bmj.com/site/about/guidelines.xhtml

Competing interests

None declared.

Data availability statement

No data are available.

References

1. Mulley AG, Trimble G EG. Stop the silent misdiagnosis: patients' preferences matter. *BMJ.* 2012;345:e6572.
2. Balogh EP, Miller BT, Ball JR. Improving diagnosis in health care. Improving Diagnosis in Health Care. 2016.p1–472.
3. Gillard S, Benson J, Silverman J. Teaching and assessment of explanation and planning in medical schools in the United Kingdom: Cross sectional questionnaire survey. *Med Teach*. 2009;31(4):328–31.
4. Zwaan L, Thijs A, Wagner C, *et al.* Does inappropriate selectivity in information use relate to diagnostic errors and patient harm? The diagnosis of patients with dyspnea. *Soc Sci Med*. 2013;91:32–8.
5. Singh H, Giardina TD, Meyer AND, *et al.* Types and origins of diagnostic errors in primary care settings. *JAMA Intern Med*. 2013;173(6):418–25.
6. Gruppen LD, Wolf FM, Billi JE. Information gathering and integration as sources of error in diagnostic decision making. *Med Decis Mak.* 1991;11(4):233–9.
7. Koo MM, von Wagner C, Abel GA, *et al.* The nature and frequency of abdominal symptoms in cancer patients and their associations with time to help-seeking: evidence from a national audit of cancer diagnosis. *J Public Health*. 2018;40(3):e388–95.
8. Rasmussen S, Haastrup PF, Balasubramaniam K, *et al.* Predictive values of upper gastrointestinal cancer alarm symptoms in the general population: a nationwide cohort study. *BMC Cancer.* 2018;18(440). https://doi.org/10.1186/s12885-018-4376-8
9. National Institute for Health and Care Excellence. Gastrointestinal tract (upper) cancers - recognition and referral (NG12). [cited 2021 Aug 3]. Available from: https://cks.nice.org.uk/topics/gastrointestinal-tract-upper-cancers-recognition-referral/
10. Calanzani N, Druce PE, Snudden C, *et al.* Identifying Novel Biomarkers Ready for Evaluation in Low-Prevalence Populations for the Early Detection of Upper Gastrointestinal Cancers: A Systematic Review. *Adv Ther*. 2020; 38(6):3032-3065
11. National Cancer Intelligence Network. Routes to Diagnosis. [cited 2021 Jan 15]. Available from: http://www.ncin.org.uk/publications/routes_to_diagnosis
12. Fitzgerald RC, di Pietro M, O'Donovan M, *et al.* Cytosponge-trefoil factor 3 versus usual care to identify Barrett's oesophagus in a primary care setting: a multicentre, pragmatic, randomised controlled trial. *Lancet.* 2020;396(10247):333–44.
13. Malterud K, Guassora AD, Graungaard AH, *et al.* Understanding medical symptoms: a conceptual review and analysis. *Theor Med Bioeth.* 2015;36(6):411–24.
14. Rosendal M, Jarbøl DE, Pedersen AF, *et al.* Multiple perspectives on symptom

For peer review only - http://bmjopen.bmj.com/site/about/guidelines.xhtml

interpretation in primary care research. *BMC Fam Pract.* 2013;14(167). https://doi.org/10.1186/1471-2296-14-167
15. Sonnenberg A, Gogel HK. Translating vague complaints into precise symptoms : the implications of a poor medical history. 2002;317–21.
16. Mills K, Birt L, Emery JD, *et al.* Understanding symptom appraisal and help-seeking in people with symptoms suggestive of pancreatic cancer: A qualitative study. *BMJ Open.* 2017;7(9):1–9.
17. Humphrys E, Walter FM, Rubin G, *et al.* Patient symptom experience prior to a diagnosis of oesophageal or gastric cancer: A multi-methods study. *BJGP Open.* 2020;4(1):1–15.
18. Bankhead CR, Collins C, Stokes-Lampard H, *et al.* Identifying symptoms of ovarian cancer: A qualitative and quantitative study. BJOG An Int J Obstet Gynaecol. 2008;115(8):1008–14.
19. Högberg C, Karling P, Rutegård J, *et al.* Patient-reported and doctor-reported symptoms when faecal immunochemical tests are requested in primary care in the diagnosis of colorectal cancer and inflammatory bowel disease: A prospective study. *BMC Fam Pract.* 2020;21(1):1–12.
20. Stapley S, Peters TJ, Neal RD, Rose PW, Walter FM, Hamilton W. The risk of pancreatic cancer in symptomatic patients in primary care: A large case-control study using electronic records. *Br J Cancer.* 2012;106(12):1940–4.
21. Stapley S, Peters TJ, Neal RD, *et al.* The risk of oesophago-gastric cancer in symptomatic patients in primary care: A large case-control study using electronic records. *Br J Cancer.* 2013;108(1):25–31.
22. Shephard E, Neal R, Rose P, *et al.* Clinical features of kidney cancer in primary care: A case-control study using primary care records. *Br J Gen Pract.* 2013;63(609):250–5.
23. Shephard EA, Hamilton W, Neal RD, *et al.* Symptoms of adult chronic and acute leukaemia before diagnosis: Large primary care case-control studies using electronic records. Br J Gen Pract. 2016;66(644):e182–8.
24. Tate AR, Martin AGR, Ali A, *et al.* Using free text information to explore how and when GPs code a diagnosis of ovarian cancer: An observational study using primary care records of patients with ovarian cancer. *BMJ Open.* 2011;1(1):1–7.
25. Koleck TA, Dreisbach C, Bourne PE, *et al.* Natural language processing of symptoms documented in free-text narratives of electronic health records: A systematic review. *J Am Med Informatics Assoc.* 2019;26(4):364–79.
26. Edwards PJ, Bennett-Britton I, Ridd MJ, *et al.* Factors affecting the documentation of spoken safety-netting advice in routine GP consultations: a cross-sectional study. *Br J Gen Pract.* 2021;*BJGP.*2021.0195.
27. Weiner SJ, Wang S, Kelly B, *et al.* How accurate is the medical record? A comparison of the physician's note with a concealed audio recording in unannounced standardized patient encounters. *J Am Med Informatics Assoc.* 2020;27(5):770–5.
28. Barnes R. One in a Million: A study of primary care consultations. 2017. [cited 2021 Oct 6]. Available from: https://doi.org/10.5523/bris.l3sq4s0w66ln1x20sye7s47wv.
29. Jepson M, Salisbury C, Ridd MJ, *et al.* The "One in a Million" study: Creating a database of UK primary care consultations. *Br J Gen Pract.* 2017;67(658):e345–51.
30. Din NU, Ukoumunne OC, Rubin G, *et al.* Age and Gender Variations in Cancer Diagnostic Intervals in 15 Cancers: Analysis of Data from the UK Clinical Practice Research Datalink. *PLoS One.* 2015;10(5).e0127717.

For peer review only - http://bmjopen.bmj.com/site/about/guidelines.xhtml

31. Hsieh HF, Shannon SE. Three approaches to qualitative content analysis. *Qual Health Res.* 2005;15(9):1277–88.
32. Bengtsson M. How to plan and perform a qualitative study using content analysis. *NursingPlus Open*. 2016;2:8–14.
33. Graneheim UH, Lindgren BM, Lundman B. Methodological challenges in qualitative content analysis: A discussion paper. *Nurse Educ Today.* 2017;56:29–34.
34. Erlingsson C, Brysiewicz P. A hands-on guide to doing content analysis. *African J Emerg Med*. 2017;7(3):93–9.
35. Silverman J, Kurtz S, Draper J. Skills for communicating with patients. 3rd ed. CRC Press Taylor & Francis Group. 2013.p69–130.
36. Procter S, Stewart K, Reeves D, *et al.* Complex consultations in primary care: A tool for assessing the range of health problems and issues addressed in general practice consultations. *BMC Fam Pract.* 2014;15(1):1–8.
37. Kurtz SM, Silverman JD. The Calgary-Cambridge Referenced Observation Guides: An aid to defining the curriculum and organizing the teaching in communication training programmes. *Med Educ.* 1996;30(2):83–9.
38. Amelung D, Whitaker KL, Lennard D, *et al.* Influence of doctor-patient conversations on behaviours of patients presenting to primary care with new or persistent symptoms: A video observation study. *BMJ Qual Saf.* 2020;29(3):198–208.
39. Vakil N, Van Zanten SV, Kahrilas P, *et al.* The Montreal definition and classification of gastroesophageal reflux disease: A global evidence-based consensus. Am *J Gastroenterol.* 2006;101(8).1900-20.
40. Drossman DA. The Functional Gastrointestinal Disorders and the Rome III Process. *Gastroenterology.* 2006;130(5):1377–90.
41. Raja SN, Carr DB, Cohen M, *et al.* The revised International Association for the Study of Pain definition of pain: concepts, challenges, and compromises. *Pain.* 2020;161(9):1976–82.
42. Meechan D, Gildea C, Hollingworth L, *et al.* Variation in use of the 2-week referral pathway for suspected cancer: A cross-sectional analysis. *Br J Gen Pract.* 2012;62(602):590–7.
43. Round T, Ashworth M, L'Esperance V, *et al.* Cancer detection via primary care urgent referral in England 2009/10 to 2018/19 and the association with practice characteristics. *Br J Gen Pract.* 2021. https://doi:10.3399/BJGP.2020.1030
44. Hippisley-Cox J, Coupland C. Identifying patients with suspected pancreatic cancer in primary care: Derivation and validation of an algorithm. *Br J Gen Pract.* 2012;62(594):38–45.
45. Hippisley-Cox J, Coupland C. Identifying patients with suspected gastro-oesophageal cancer in primary care: derivation and validation of an algorithm. *Br J Gen Pr.* 2011;e707-e714.
46. British Medical Association. Access to health records - Updated to reflect the General Data Protection Regulation and Data Protection Act 2018. 2018.
47. Colicchio TK, Cimino JJ. Clinicians' reasoning as reflected in electronic clinical note-entry and reading/retrieval: a systematic review and qualitative synthesis. *J Am Med Informatics Assoc.* 2019;26(2):172–84.
48. Renzi C, Lyratzopoulos G. Comorbidity and the diagnosis of symptomatic-but-as-yet-undiagnosed cancer. *Br J Gen Pract.* 2020;70(698):E598–9.
49. Britten N, Stevenson F, Barry C, *et al.* Patients' unvoiced agendas in general practice

For peer review only - http://bmjopen.bmj.com/site/about/guidelines.xhtml

consultations: qualitative study. *Gen Pract.* 2000;320:484–8.
50. Elliss-Brookes L, McPhail S, Ives A, *et al.* Routes to diagnosis for cancer - Determining the patient journey using multiple routine data sets. *Br J Cancer.* 2012;107(8):1220–6.
51. Singh H, Davis Giardina T, Petersen LA, *et al.* Exploring Situational Awareness in Diagnostic Errors in Primary Care Conclusions—A framework of SA can help analyze and understand diagnostic errors in primary care settings that use EHRs. *BMJ Qual Saf*. 2012;21(110):30–8.
52. Lyratzopoulos G, Vedsted P, Singh H. Understanding missed opportunities for more timely diagnosis of cancer in symptomatic patients after presentation. *Br J Cancer.* 2015;112:S84–91.
53. Zhou Yin, Mendonca SC, Abel GA, *et al.* Variation in "fast-track" referrals for suspected cancer by patient characteristic and cancer diagnosis: evidence from 670 000 patients with cancers of 35 different sites. *Br J Cancer.* 2017;118:24–31.
54. Forrest LF, Sowden S, Rubin G, *et al.* Socio-economic inequalities in stage at diagnosis, and in time intervals on the lung cancer pathway from first symptom to treatment: Systematic review and meta-analysis. *Thorax.* 2017;72(5):430–6.
55. Zhou Y, van Melle M, Singh H, *et al.* Quality of the diagnostic process in patients presenting with symptoms suggestive of bladder or kidney cancer: a systematic review. *BMJ Open.* 2020;9(10).e029143
56. Lyratzopoulos G, Abel GA, McPhail S, *et al.* Gender inequalities in the promptness of diagnosis of bladder and renal cancer after symptomatic presentation: Evidence from secondary analysis of an English primary care audit survey. *BMJ Open.* 2013;3(6):1–7.
57. Kreatsoulas C, Crea-Arsenio M, Shannon HS, *et al.* Interpreting angina: symptoms along a gender continuum. *Open Heart.* 2016;3(1):e000376.
58. Willems S, De Maesschalck S, Deveugele M, *et al.* Socio-economic status of the patient and doctor-patient communication: Does it make a difference? *Patient Educ Couns.* 2005;56(2):139–46.
59. 98point6. On-demand, text-based primary care. [cited 2021 Oct 6]. Available from: https://www.98point6.com/

For peer review only

For peer review only - http://bmjopen.bmj.com/site/about/guidelines.xhtml

For peer review only

For peer review only - http://bmjopen.bmj.com/site/about/guidelines.xhtml

Figure 1. Flow diagram of included consultations

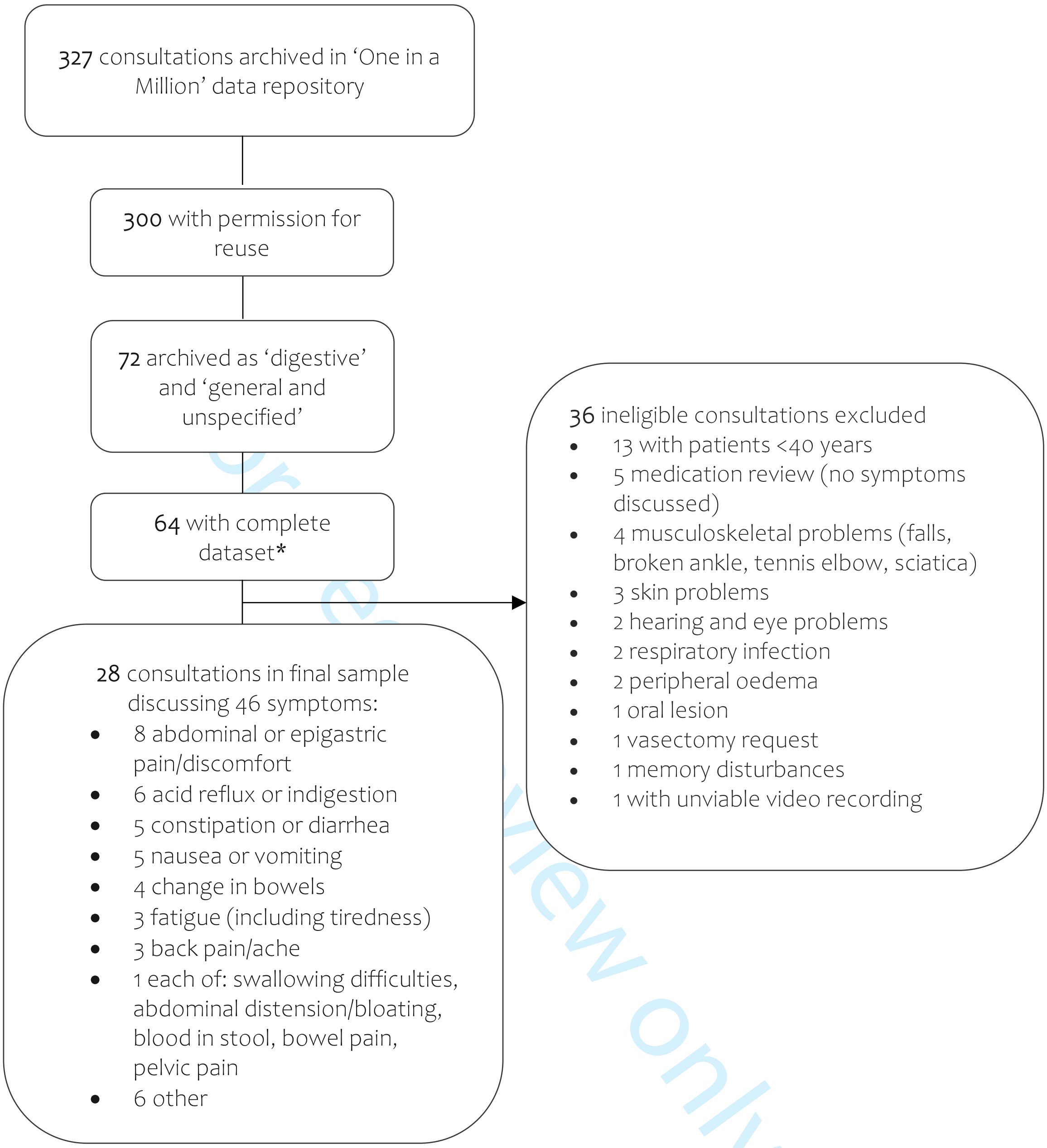

*Video recording, transcript, and medical record entry

For peer review only

Figure 2. Final categories representing the clinical features of abdominal sensations discussed in primary care consultations based on the 'gathering information' domains of the Calgary-Cambridge model

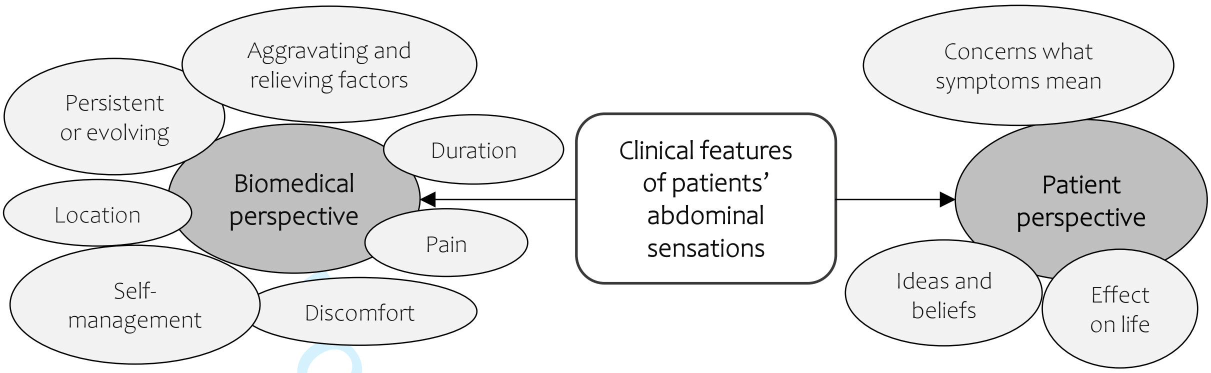

For peer review only

Figure 3. Frequency of alignments and misalignments for each feature of patients' abdominal and systemic bodily sensations, according to the 'gathering information' domain of the Calgary-Cambridge Guide

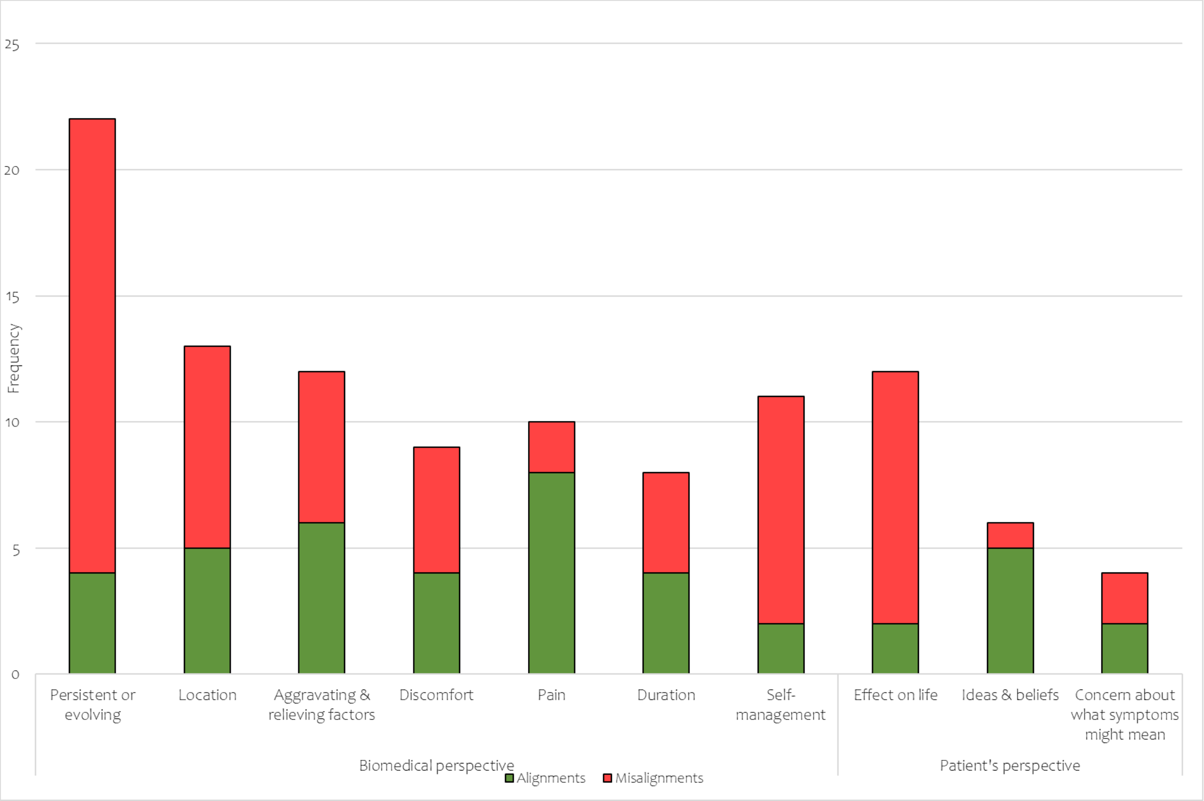

Supplementary file A. Details of included patients and their upper GI/systemic bodily sensations

Patient ID	Gender	Age	Bodily sensations
1	Female	66	Follow-up post-emergency operation for strangulated hernia, 'sticky bowels'
2	Female	71	Nausea, acid reflux
3	Female	72	Abdominal and pelvic pain, back ache
4	Male	55	Abdominal pain
5	Female	65	Nausea
6	Female	79	Heartburn, nausea
7	Female	73	Abdominal pain
8	Male	65	Abdominal and rectal pain, loose stools
9	Male	60	Acid reflux, indigestion
10	Male	59	Tiredness, headaches
11	Female	69	Abdominal distension/bloated
12	Male	73	Bowel pain, constipation
13	Male	83	Swallowing difficulties
14	Female	58	Back pain
15	Female	45	Fatigue, difficulty urinating
16	Male	81	Constipation
17	Female	77	Epigastric and back pain, nausea and vomiting
18	Female	53	Abdominal pain, nausea, diarrhoea, blood in stools
19	Female	42	Heartburn
20	Female	72	Reflux
21	Male	71	Loose stools, colitis flare-up
22	Female	41	Change in bowels, low haemoglobin
23	Female	70	Abdominal pain, change in bowels
24	Male	43	Fatigue, change in bowels
25	Female	59	Voice hoarseness, stomach problems
26	Male	74	Hernia
27	Male	64	Gastritis, follow-up of biopsy reversal
28	Female	53	Epigastric discomfort

For peer review only

COREQ (COnsolidated criteria for REporting Qualitative research) Checklist

A checklist of items that should be included in reports of qualitative research. You must report the page number in your manuscript where you consider each of the items listed in this checklist. If you have not included this information, either revise your manuscript accordingly before submitting or note N/A.

Topic	Item No.	Guide Questions/Description	Reported on Page No.
**Domain 1: Research team and reflexivity**			
Personal characteristics			
Interviewer/facilitator	1	Which author/s conducted the interview or focus group?	N/A
Credentials	2	What were the researcher's credentials? E.g. PhD, MD	15-16
Occupation	3	What was their occupation at the time of the study?	15-16
Gender	4	Was the researcher male or female?	N/A
Experience and training	5	What experience or training did the researcher have?	16
Relationship with participants			
Relationship established	6	Was a relationship established prior to study commencement?	N/A
Participant knowledge of the interviewer	7	What did the participants know about the researcher? e.g. personal goals, reasons for doing the research	N/A
Interviewer characteristics	8	What characteristics were reported about the inter viewer/facilitator? e.g. Bias, assumptions, reasons and interests in the research topic	N/A
**Domain 2: Study design**			
Theoretical framework			
Methodological orientation and Theory	9	What methodological orientation was stated to underpin the study? e.g. grounded theory, discourse analysis, ethnography, phenomenology, content analysis	2, 5
Participant selection			
Sampling	10	How were participants selected? e.g. purposive, convenience, consecutive, snowball	6
Method of approach	11	How were participants approached? e.g. face-to-face, telephone, mail, email	5, 6
Sample size	12	How many participants were in the study?	2, 7, 8
Non-participation	13	How many people refused to participate or dropped out? Reasons?	N/A
Setting			
Setting of data collection	14	Where was the data collected? e.g. home, clinic, workplace	5
Presence of non-participants	15	Was anyone else present besides the participants and researchers?	5
Description of sample	16	What are the important characteristics of the sample? e.g. demographic data, date	8
Data collection			
Interview guide	17	Were questions, prompts, guides provided by the authors? Was it pilot tested?	N/A
Repeat interviews	18	Were repeat inter views carried out? If yes, how many?	N/A
Audio/visual recording	19	Did the research use audio or visual recording to collect the data?	2, 5
Field notes	20	Were field notes made during and/or after the inter view or focus group?	6, 7
Duration	21	What was the duration of the inter views or focus group?	N/A
Data saturation	22	Was data saturation discussed?	N/A
Transcripts returned	23	Were transcripts returned to participants for comment and/or	N/A

For peer review only

Topic	Item No.	Guide Questions/Description	Reported on Page No.
		correction?	
**Domain 3: analysis and findings**			
Data analysis			
Number of data coders	24	How many data coders coded the data?	6-7
Description of the coding tree	25	Did authors provide a description of the coding tree?	6-7
Derivation of themes	26	Were themes identified in advance or derived from the data?	6-7
Software	27	What software, if applicable, was used to manage the data?	6-7
Participant checking	28	Did participants provide feedback on the findings?	15
Reporting			
Quotations presented	29	Were participant quotations presented to illustrate the themes/findings? Was each quotation identified? e.g. participant number	11-14
Data and findings consistent	30	Was there consistency between the data presented and the findings?	11-14
Clarity of major themes	31	Were major themes clearly presented in the findings?	11-14
Clarity of minor themes	32	Is there a description of diverse cases or discussion of minor themes?	11-14

Developed from: Tong A, Sainsbury P, Craig J. Consolidated criteria for reporting qualitative research (COREQ): a 32-item checklist for interviews and focus groups. *International Journal for Quality in Health Care*. 2007. Volume 19, Number 6: pp. 349 – 357

Once you have completed this checklist, please save a copy and upload it as part of your submission. DO NOT include this checklist as part of the main manuscript document. It must be uploaded as a separate file.

For peer review only

Agreement between patient's description of abdominal symptoms of possible upper gastrointestinal cancer and General Practitioner consultation notes: a qualitative analysis of video-recorded UK primary care consultation data

Journal:	*BMJ Open*
Manuscript ID	bmjopen-2021-058766.R1
Article Type:	Original research
Date Submitted by the Author:	06-Apr-2022
Complete List of Authors:	Hardy, Victoria; University of Cambridge, Public Health and Primary Care<br>Usher-Smith, Juliet; University of Cambridge, Department of Public Health and Primary Care<br>Archer, Stephanie; University of Cambridge, Department of Public Health and Primary Care<br>Barnes, Rebecca ; University of Oxford Nuffield Department of Primary Care Health Sciences<br>Lancaster, John; University of Cambridge, Patient Representative c/o Public Health & Primary Care<br>Johnson, Margaret ; University of Cambridge, Patient Representative c/o Public Health & Primary Care<br>Thompson, Matthew; University of Washington, Department of Family Medicine<br>Emery, Jon; University of Melbourne Victorian Comprehensive Cancer Centre, Centre for Cancer Research and General Practice and Primary Care Academic Centre<br>Singh, Hardeep; Michael E DeBakey VA Medical Center, Houston Veterans Affairs Center for Innovations in Quality, Effectiveness and Safety; Baylor College of Medicine, Department of Medicine<br>Walter, Fiona; Queen Mary University of London, Wolfson Institute of Population Health, Barts and the London School of Medicine and Dentistry; University of Cambridge, Department of Public Health and Primary Care
**Primary Subject Heading**:	General practice / Family practice
Secondary Subject Heading:	Qualitative research, Evidence based practice, Medical education and training
Keywords:	PRIMARY CARE, QUALITATIVE RESEARCH, MEDICAL EDUCATION & TRAINING

SCHOLARONE™
Manuscripts

I, the Submitting Author has the right to grant and does grant on behalf of all authors of the Work (as defined in the below author licence), an exclusive licence and/or a non-exclusive licence for contributions from authors who are: i) UK Crown employees; ii) where BMJ has agreed a CC-BY licence shall apply, and/or iii) in accordance with the terms applicable for US Federal Government officers or employees acting as part of their official duties; on a worldwide, perpetual, irrevocable, royalty-free basis to BMJ Publishing Group Ltd ("BMJ") its licensees and where the relevant Journal is co-owned by BMJ to the co-owners of the Journal, to publish the Work in this journal and any other BMJ products and to exploit all rights, as set out in our licence.

The Submitting Author accepts and understands that any supply made under these terms is made by BMJ to the Submitting Author unless you are acting as an employee on behalf of your employer or a postgraduate student of an affiliated institution which is paying any applicable article publishing charge ("APC") for Open Access articles. Where the Submitting Author wishes to make the Work available on an Open Access basis (and intends to pay the relevant APC), the terms of reuse of such Open Access shall be governed by a Creative Commons licence – details of these licences and which Creative Commons licence will apply to this Work are set out in our licence referred to above.

Other than as permitted in any relevant BMJ Author's Self Archiving Policies, I confirm this Work has not been accepted for publication elsewhere, is not being considered for publication elsewhere and does not duplicate material already published. I confirm all authors consent to publication of this Work and authorise the granting of this licence.

For peer review only

Agreement between patient's description of abdominal symptoms of possible upper gastrointestinal cancer and General Practitioner consultation notes: a qualitative analysis of video-recorded UK primary care consultation data

Victoria Hardy[1], Juliet Usher-Smith[1], Stephanie Archer[1], Rebecca K. Barnes[2], John Lancaster[1], Margaret Johnson[1], Matthew Thompson[3], Jon Emery[4], Hardeep Singh[5], Fiona M. Walter[6,1]

Corresponding author

Victoria Hardy, Department of Public Health & Primary Care, University of Cambridge, Cambridge, UK

veh29@medschl.cam.ac.uk

Affiliations

[1]Department of Public Health & Primary Care, University of Cambridge, Cambridge, UK

[2]Nuffield Department of Primary Care Health Sciences, University of Oxford, Oxford, UK

[3]Department of Family Medicine, University of Washington, Seattle, Washington, USA

[4]Centre for Cancer Research and Department of General Practice, University of Melbourne, Victorian Comprehensive Cancer Centre, Melbourne, Victoria, Australia

[5]Center for Innovations in Quality, Effectiveness and Safety, Michael E. DeBakey VA Medical Center and Baylor College of Medicine, Houston, TX, USA.

[6]Wolfson Institute of Population Health, Barts and the London School of Medicine and Dentistry, Queen Mary University of London, UK

Key words: primary care, symptoms, diagnosis, qualitative research

Word count: 4493

For peer review only - http://bmjopen.bmj.com/site/about/guidelines.xhtml

Abstract

Introduction

Abdominal symptoms are common in primary care but infrequently might be due to an upper gastrointestinal (UGI) cancer. Patients' descriptions may differ from medical terminology used by General Practitioners (GPs). This may affect how information about abdominal symptoms possibly due to an UGI cancer are documented, creating potential missed opportunities for timely investigation.

Objectives

To explore how abdominal symptoms are communicated during primary care consultations, and identify characteristics of patients' descriptions that underpin variation in the accuracy and completeness with which they are documented in medical records.

Methods and analysis

Primary care consultation video recordings, transcripts and medical records from an existing dataset were screened for adults reporting abdominal symptoms. We conducted a qualitative content analysis to capture alignments (medical record entries matching patient verbal and non-verbal descriptions) and misalignments (symptom information omitted or differing from patient descriptions). Categories were informed by the Calgary-Cambridge Guide's 'gathering information' domains and patterns in descriptions explored.

Results

Our sample included 28 consultations (28 patients with 18 GPs): ten categories of different clinical features of abdominal symptoms were discussed. The information GPs documented about these features commonly did not match what patients described, with misalignments more common than alignments (71 vs 40 instances, respectively). Misalignments often featured patients using vague descriptors, figurative speech, lengthy explanations and broad hand gestures. Alignments were characterised by patients using well-defined terms, succinct descriptions and precise gestures for symptoms with an exact location. Abdominal sensations reported as 'pain' were almost always documented compared to expressions of 'discomfort'.

Conclusions

Abdominal symptoms that are well-defined or communicated as 'pain' may be more salient to GPs than those expressed vaguely or as 'discomfort'. Variable documentation of abdominal symptoms in medical records may have implications for the development of clinical decision support systems and decisions to investigate possible UGI cancer.

For peer review only - http://bmjopen.bmj.com/site/about/guidelines.xhtml

ARTICLE SUMMARY

Strengths and limitations

- This is the first study to explore similarities and differences between how patients describe and GPs document abdominal symptoms that could be due to an UGI cancer using naturalistic UK primary care consultation data
- We did not have access to patients' previous consultation history and so could not determine the clinical importance of symptom information that was either not documented in medical records, or did not reflect the patient's history.
- As our sample was mostly White British and native English speakers, patterns in how patients described and GPs documented abdominal symptoms may not be transferable to practices serving patient populations with different characteristics

For peer review only

Introduction

Sir William Osler, lauded as the father of modern medicine, implored his medical students to *"listen to your patient: he is telling you the diagnosis"*.(1) Over a century later, this adage remains germane. As depicted in the National Academy of Medicine's (NAM) conceptual model of the diagnostic process, an accurate and timely diagnosis is predicated on clinicians collecting sufficient data from the patient history.(2) Communication skills teaching is integral to medical training,(3) but suboptimal information gathering is common during the medical interview(4–6) and has been implicated in diagnostic error in primary care settings.(5)

Abdominal symptoms are common in patients presenting to primary care.(7) While such symptoms far more frequently herald benign conditions,(8) they are also described by patients diagnosed with upper gastrointestinal (UGI) cancers (i.e., gastric, oesophageal, and pancreatic), as well as other cancers within the abdomen.(7) In the United Kingdom (UK), compared with 'alarm' symptoms which have relatively high positive predictive value (PPV), abdominal symptoms alone do not meet the 3% PPV threshold recommended by the National Institute for Clinical Excellence (NICE) NG12 (2015) for a 'fast-track' suspected cancer referral.(9) Compounded by a lack of screening programmes or biomarkers reliably raising suspicion of UGI cancer in low prevalence populations,(10) patients with UGI cancers are disproportionately burdened with lengthy diagnostic intervals and late-stage diagnosis, associated with worse survival and patient experiences of healthcare.(11) While the novel 'CytoSponge' test (for Barrett's oesophagus, a precursor lesion for oesophageal cancer(12)) is a promising development for early detection, effective triage of abdominal symptoms for a timely UGI cancer diagnosis, remains currently largely contingent upon the patient history.

Symptoms are subjectively experienced changes in bodily sensations that reflect a perceived departure from the body's "normal" state.(13,14) Patients' experiences of bodily changes are the convergence of sociocultural(13) and experiential factors,(14) and may be communicated differently from standardised medical terminology used by general practitioners (GPs).(15) Studies of UGI cancer patients' symptom appraisal have shown they use a wide range of words and phrases to conceptualise abdominal changes(16,17) that

For peer review only - http://bmjopen.bmj.com/site/about/guidelines.xhtml

are inconsistent with medical definitions familiar to GPs.(17,18) Without a common language, potentially significant bodily sensations risk being overlooked or misinterpreted, and the clinical picture distorted as 'lay' descriptions are translated into equivalent medical nomenclature.(15) Consequently, what GPs document in medical records may not be a complete or accurate representation of the patient's history,(19) potentially creating opportunities for diagnostic delay.

As well as being important at the patient-level for subsequent management decisions, the ways GPs (and other equivalent primary care providers in jurisdictions outside the UK) document patients' bodily sensations in medical records is important for population approaches to early cancer detection. Initiatives in the UK currently harvest coded symptom data from primary care records to develop national guidelines and prediction tools that help GPs estimate patients' current or future cancer risk.(20–23) However, there is mounting interest in harnessing Natural Language Processing techniques to incorporate free-text symptoms.(24,25) Determining how patients describe abdominal sensations to GPs and the extent to which medical records reflect the patient's history is paramount for ensuring clinical decisions tools for UGI (and other) cancers facilitate effective use of tests and referral pathways.

To address this research need, we compared video-recorded primary care consultations featuring new or ongoing abdominal sensations that could be due to an UGI cancer with medical record documentation and explored similarities and differences between patient descriptions of bodily sensations and the way GPs document these as symptoms. We aimed to explore how abdominal symptoms are recorded in the medical record to identify the characteristics of patients' descriptions underpinning GP variation in the medical record entries and the implications of this on opportunities for earlier diagnosis of UGI cancers. This study builds on research from the wider literature that has utilised naturalistic data to quantify the concordance between GPs' spoken and documented safety-netting advice,(26) and information captured in medical records by physicians compared to details given by unannounced standardised patients (USPs).(27)

Methods

Design

A cross-sectional descriptive study of video-recorded primary care consultations with linked medical record data entries from the *"One in a Million"* primary care consultation archive, analyzed using qualitative content analysis.

Data source

For peer review only - http://bmjopen.bmj.com/site/about/guidelines.xhtml

The *"One in a Million"* dataset was derived from the 'Bristol Archive project,' a prospective observational cohort study that sought to create a repository of routine primary care consultation data available for use in future research.(28) Between July 2014 and April 2015, twelve purposively sampled general practices in the West of England recorded unselected consultations with adult patients (aged 18 years and above) who presented to primary care over two to three half days.(29) A total of 327 consultations were recorded with linked patient, GP and practice demographic data, and available medical record entries. 89.8% (n=300) of patients consented to future data access by other researchers, subject to specific ethical approval. All these recordings were anonymized for spoken identifiers, transcribed verbatim and coded for their content according to sixteen "problem type(s)" based on the International Classification of Primary Care – 2nd Edition (ICPC-2) classification scheme.(29) Consultations were assigned more than one code if multiple problems were discussed. We requested all available data for 72 consultations archived as 'digestive' or 'general and unspecified'(29) to yield data directly relevant for addressing our research question.

Eligibility criteria and assessment

We included consultations if there was: a) an accompanying videorecording, transcript, and medical record entry, and b) communication of any new or ongoing abdominal and systemic sensations that could reflect an underlying UGI cancer. We limited inclusion to patients aged 40 years and above, because UGI cancers below this age are rare.(30) Consultations of patients known to have a previous or current cancer diagnosis based on information disclosed during the video-recording were excluded, as a history of cancer provokes more suspicion of serious illness among GPs and patients, and we were interested in GPs' appraisal of patient's bodily sensations in an as-yet undiagnosed population. We also excluded consultations during which no relevant bodily sensations were communicated. This was determined by abstracting all bodily sensations discussed in consultations into a pre-prepared Microsoft Excel form and assessing each as 'include', 'exclude', or 'unsure'. Eligibility assessment was initially undertaken by VH (a non-clinical PhD student) before final adjudication of relevant bodily sensations by FW (a senior academic GP). Consultations for which there were no relevant bodily sensations were excluded at this juncture.

Sampling

As this study was an analysis of a pre-existing archived dataset and no further data collection was possible, we included all consultations of symptomatic patients that met study eligibility criteria.

Data analysis

For peer review only - http://bmjopen.bmj.com/site/about/guidelines.xhtml

Data were analysed using a manifest qualitative content analysis, following the four phases outlined by Erlingsson *et al.*: 'familiarising oneself with the data', 'dividing and condensing the text into meaning units', 'formulating codes', and 'developing categories and themes'.(31) A manifest qualitative content analysis was selected as it is the most suitable analytical technique for exploring and describing visible and explicit patterns (as opposed to the underlying meaning of text as in latent content analysis) across data sources that incorporate visual and textual communication data.(32–34) Data analysis was conducted in excel by VH, with analysis facilitated by SA (senior non-clinical researcher), and data interpretation verified by FW, JUS (senior academic GPs), and PPI (JL, MJ).

We employed a mixed deductive and inductive approach whereby the analytical framework was informed by the 'gathering information' domains of the Calgary-Cambridge Guide. We selected the Calgary-Cambridge Guide over other possible instruments (e.g., the Roter Interaction Analysis System) as it is an evidence-based teaching and assessment resource that is used in medical training programmes internationally to develop doctors' verbal and non-verbal communication skills and structure consultations. The guide delineates the consultation into distinct phases, including 'initiating the session', 'gathering information', 'physical examination', 'explanation and planning', and 'closing the session'. The 'gathering information' component guides doctors through the collection of symptom information relating to the biomedical perspective, patient perspective, and background information that should be elicited during the patient history. Thus, the Calgary-Cambridge Guide offers an ecologically valid framework for guiding the deconstruction and analysis of the information communicated and documented in primary care consultations.

Analysis begun by cross-referencing transcripts with video-recordings to verify accuracy. Transcripts, video-recordings, and medical record entries were then reviewed repeatedly, individually and concomitantly, to become familiar with the content. Video-recordings were reviewed alongside transcripts to observe the nonverbal body language exhibited by patients and GPs. Six nonverbal aspects of communication were annotated in parentheses adjacent to the relevant text. These included posture, proximity, touch, body movements, facial expression, and eye behaviour.(35)

Short verbatim excerpts or 'meaning units' (encompassing nonverbal gestures) were juxtaposed with medical record entries using an adapted consultation proforma originally developed for examining the content of different primary care data sources.(36) Throughout analysis, excerpts from each consultation were kept together to allow contextualization of the data. 'Meaning units' were condensed by VH into an abbreviated narrative capturing the essence of patient's reason for presentation. Care was taken to preserve the patient voice by weaving verbatim descriptions from excerpts into a reconfigured narrative. Patient reported bodily sensations were then compared with

For peer review only - http://bmjopen.bmj.com/site/about/guidelines.xhtml

symptoms documented by GPs in the patients' medical record, and alignments and misalignments in documentation coded. Alignments were symptom information in medical records that both mirrored the content and verbal and non-verbal descriptors patients communicated; misalignments were symptoms that had either not been documented or differed from information given by patients. This task was independently undertaken by VH and two patient and public representatives (PPI) (MJ and JL); differences were resolved through further discussion and consensus with FW and JUS. Coded excerpts constituted the unit of analysis and encompassed multiple features of an individual symptom.

Coded alignments and misalignments were separated from original data sources and, with regular input from study authors, iteratively grouped into descriptive categories, informed by the symptom features listed in the 'gathering information' domains.(35)

Final categories were re-contextualised, and the features of patient's verbal descriptions and non-verbal gestures underpinning alignments and misalignments explored. Attention was given to isolated and discrepant excerpts to illuminate as much diversity of symptom expression encountered in general practice. We also explored differences in GP documentation by GP and patient demographic characteristics.

Ethical considerations

Research ethics approval was obtained from the East of Scotland Research Ethics Committee (REC) prior to commencing the study (reference: 19/ES/0057).

Patient and public involvement

Two patient and public involvement representatives (JL, ML) actively advised the research group from study inception to manuscript preparation. Both representatives were involved in the analysis and interpretation of the data, and will facilitate dissemination of study findings.

Results

Included consultations

Of the 72 consultations requested from the 'One in a Million' data repository, 28 met study eligibility criteria and were included in the final analysis (Figure 1). Participants comprised 28 patients, one caregiver, and 18 GPs. Overall, patients presented with 46 new or ongoing problems, captured in a total of 111 textual excerpts that were relevant for this analysis.

INSERT: Figure 1. Flow diagram of included consultations

Sample characteristics

For peer review only - http://bmjopen.bmj.com/site/about/guidelines.xhtml

Characteristics of included patients and GPs are presented in **Table 1** (the one caregiver's characteristics were unavailable). Patients were mostly female (*n*=17), aged 40 to 89 years, with varying educational attainment. GPs were aged 30 to 69 years and had been qualified as a GP for up to 31 years. Patients and GPs were all born in the UK and spoke English as their primary language. Apart from one patient of mixed ethnicity, our sample was white British. Patients presented with a variety of UGI and systemic bodily sensations (**Supplementary File A**).

For peer review only

Table 1. Sample characteristics

Patient characteristics	Number of patients (*N*=28)	GP characteristics	Number of GPs (*N*=18)
Sex		Sex	
Male	11	Male	10
Female	17	Female	8
Ethnicity*		Ethnicity	
White British/White other	26	White British/White other	18
Mixed/multiple ethnic groups	1	Mixed/multiple ethnic groups	-
Age		Age	
30-39	NA	30-39	5
40-49	5	40-49	5
50-59	6	50-59	7
60-69	6	60-69	1
70-79	9	70-79	-
80-89	2	80-89	-
Main language*		Main language	
English	27	English	18
Country of birth		Country of birth	

For peer review only - http://bmjopen.bmj.com/site/about/guidelines.xhtml

UK	28	UK	18
**Highest educational attainment**		**Years since qualified as a GP**	
Degree or higher degree	8	1-10	5
A/AS-levels or advanced diploma	2	11-20	6
Professional or vocational qualifications	6	21-30	6
Apprenticeship	3	31+	1
No qualifications	4		
**IMD quintiles**			
1st (least deprived)	6		
2nd	8		
3rd	4		
4th	5		
5th (most deprived)	5		

*Not reported (*n*=1); IMD, index of multiple deprivation; NA, not applicable (not eligible for inclusion)

For peer review only

GP alignments and misalignments

Overall, there was more evidence of GP misalignments (*n*=71) than alignments (*n*=40), with a median of 2 (*range:* 0–8) misalignments and 1 (*range:* 0–5) alignments, per consultation.

We identified ten categories reflecting ten clinical features which were informed by the 'gathering information' domains of the Calgary-Cambridge Guide (Figure 2 and Table 2).(35) Of these ten categories/features, seven related to the *'biomedical perspective',* and three to the *'patient's perspective'* (Figure 2). Most misalignments occurred for abdominal sensations that were 'persistent or evolving', or when documenting the 'location', 'effect of on patients' life', and 'self-management' of abdominal sensations (Figure 3). Alignments were most common when abdominal sensations were described as 'pain' compared to 'discomfort', and when capturing patient's ideas and beliefs. Findings for all ten clinical features are detailed below. A summary of the main differences in patient descriptions of each feature between alignments and misalignments is presented in Table 2.

INSERT: Figure 2. Final categories representing the clinical features of abdominal sensations discussed in primary care consultations based on the 'gathering information' domains of the Calgary-Cambridge model

INSERT: Figure 3. Frequency of alignments and misalignments for each feature of patients' abdominal and systemic bodily sensations, according to the 'gathering information' domain of the Calgary-Cambridge Guide

Table 2. Differences in the characteristics of patients' verbal and non-verbal descriptions of abdominal sensations for alignments and misalignments

Clinical feature	Alignments	Misalignments
Domain: Biomedical perspective		

For peer review only - http://bmjopen.bmj.com/site/about/guidelines.xhtml

Persistent or evolving	Well-defined terms, articulating changes in quality, frequency, and intensity of worsening sensations	Non-definitive, figurative speech for bodily sensations that were worsening, improving but not resolved, or re-appraised as worsening
Location of pain/discomfort	Well-defined or medical terms, succinctly communicated; non-verbal gestures pointing to site of pain/discomfort	Difficulty articulating precise site of pain/discomfort; broad or global non-verbal gestures not indicating site of pain/discomfort
Aggravating and relieving factors	Communication of a relationship between activities of daily living and onset or worsening of sensations	Infrequent provocation of sensations; lengthy explanations regarding activities that provoked or worsened sensations; activities that relieved sensations
Discomfort	Adjectives or figurative speech for unpleasant sensations	Adjectives or figurative speech for unpleasant sensations
Pain	Pain explicitly articulated with qualifying adjectives, similes, and verbs describing an aversive sensation	Pain explicitly articulated with qualifying adjectives, similes, and verbs describing an aversive sensation
Duration	Communicating the day or number of weeks or months since onset of symptoms	Vague or oblique sentences without offering a timeframe
Self- management	Reporting sensations that were controlled or settling with medication	Reporting suboptimal therapeutic effect of medication
**Domain: Patient's perspective**		
Effects on life	*(no differences in patient descriptions between alignments and misalignments)*	
Ideas and beliefs	Plausible and convincing explanation as to underlying cause related to medication side-effects, flare-up of pre-existing condition, diet, or stress	Lack of a justified explanation for possible cause of sensations
Concerns about what symptoms might mean	Explicit concerns voiced about the possibility of cancer, or more broad concerns that something was not right	Verbalising concerns about the possibility symptoms had a sinister cause, but not explicitly mentioning cancer

Domain: Biomedical perspective

Persistent or evolving

Abdominal sensations that were persistent or evolving in frequency or intensity were described by patients most often, though were poorly reflected in medical records. Alignments occurred when patients used well-defined or concrete terms to indicate that their bodily sensations were progressing:

**Patient:** *"my poo has got a lot freer and a lot looser probably in about the last six weeks. Most of the time*	**Medical records:** last *6 weeks loose stools and more freq (GP19)*

when I go to the loo I will do some sort of poo as well at the same time. (shakes head)" (P22: F, 40-49 years)

Abdominal sensations described in non-definitive ("not getting better") or figurative speech, that were receding but not resolved, or that patients re-appraised as worsening, were characteristic of misalignments. In these instances, bodily sensations were erroneously documented as normal, resolving, or stabilised, respectively:

Patient: *"bowels are a bit sticky (intermittent eye contact, squeezing toothpaste motion with both hands) but I've been eating prunes and apricots and (laughs)*<br>*GP: "And do you tend to have problems in that area anyway?"*<br>***Patient:*** *"Not normally (looks upwards) and that was part of the symptoms last week" (P1: F, 60-69)*	Medical records: *constipation improving with prunes. (GP23)*

Location of pain/discomfort

The location of patients' problem was discussed in relation to abdominal 'pain' or 'discomfort' on thirteen occasions. Misalignments were slightly more common than alignments. They occurred when pain/discomfort was communicated to be diffuse, move around, radiate to another location, or in instances when patients were unable to articulate the precise location and relied on large non-verbal hand gestures.

GP: *" So where are you point- (tilts head) is it the middle of the belly, upper belly?"*<br>Patient: *"I'm not really sure, (looking down at abdomen) but I'm just going like that with it, (rocking back and forth both arms cradling abdomen) because it..." (P18: F, 50-59 years)*	Medical records: *nothing recorded in relation to location (GP15)*

Location of abdominal pain/discomfort was accurately documented when a specific anatomical site was isolated, communicated in succinct and well-defined (sometimes medical) terms, and supplemented with non-verbal gestures that reinforced the specific site of their pain/discomfort:

Patient: *"I've had an abdominal discomfort for about four or five weeks. It's just sore, really, just below the sternum. (tips of fingers press into epigastric region). (P28: F, 50-59 years)*	Medical records: *epigastric pain (GP12)*

For peer review only - http://bmjopen.bmj.com/site/about/guidelines.xhtml

Aggravating and relieving factors

Aggravating and relieving factors referred to activities of daily living that provoked, aggravated, or worsened patients' bodily sensations. GPs' medical record entries accurately reflected information patients communicated in relation to this feature, when a causal relationship between an activity (i.e., stress, medication, and certain foods) and the onset or worsening of sensations was articulated:

Patient: *"It's like cucumber, red onion, tomatoes; it's certain things. I love to eat jacket potatoes, it just sets off heartburn. (P19: F, 40-49 years)*	Medical records: *Gastro-oesophageal reflux: with certain foods (GP18)*

Information about activities aggravating bodily sensations were omitted from medical records when they were described as infrequent or improving, gave lengthy explanations of the precipitating factors, or in one instance, communicated an activity that eased bodily sensations:

Patient: *"I have weeks now as long as I don't eat sugar and I don't have any stress it's often really quite good [...] At the moment it's a bit worse because I'm fighting for my son's funding and that gets me stressed. (shifts in chair, strokes forehead) Mostly it's so much better." (P25: F, 50-59 years)*	Medical records: *nothing recorded in relation to aggravating and relieving factors (GP20)*

Discomfort

Though there were no differences in patients' communication of discomfort between alignments and misalignments, patients used a range of adjectives and similes to describe abdominal sensations that were causing discomfort. Subtle verbal descriptors, such as "a nuisance" or as though "something was pressing down" conveyed discomfort as a sensation that was interfering or unpleasant. These descriptions were omitted or inaccurately reflected by GPs in most instances:

Patient: *"I can still feel a little something there (left hand touches hollow of neck) [...] a sensation up here but it can't be a physical blockage because they looked [...] it's just I feel like you've got an airlock..." (P20: F, 70-79 years)*	Medical records: *nothing recorded in relation to the quality of the sensation (GP18)*

Pain

GPs almost always documented 'pain' when it was mentioned. There were no observable differences in how patients described pain between alignments and the two misalignments. Patients also qualified 'pain' with supplementary adjectives, verbs, and similes, such as "terrible", "stabbing", and "like waves, like contractions", that communicated an aversive

For peer review only - http://bmjopen.bmj.com/site/about/guidelines.xhtml

sensation. Such qualifiers were characteristic of both alignments and misalignments and rarely captured in medical records.

Duration

The duration of patients' bodily sensations was infrequently described. GPs' accurately documented what patients communicated in relation to this feature in half of the eight instances it was discussed. Offering a discrete timeframe by giving the day of the week or number of weeks or months that had elapsed since the onset of bodily sensations, was equally characteristic of misalignments and alignments:

GP: *"So how long have you been poorly, now?"*<br>Patient: *"Well, it wasn't last week. What's today? Tuesday. It was the Friday week. Last Friday week, yes" (nods head) (P3: F, 70-79 years)*	Medical records: *lower abdo pelvic pain for 10 days (GP5)*

In contrast, vague or open-ended descriptions of the time since onset were slightly more typical of this feature being omitted from medical records.

Self-management

Patients described use of prescription and over-the-counter medication taken to manage their bodily sensations. GP misalignments, which were more commonplace than alignments, were mostly characterised by lengthy explanations that the medication had eased but not resolved, or had failed to appreciably control the severity of bodily sensations:

Patient: *"I took those capsules. I took one in the morning before anything else. And I took one at night before a meal [...] It [upper abdominal pain] settled a little bit. But it's still there (right hand presses upper abdomen) (P17: F, 70-79 years)*	Medical records: n*othing recorded in relation to self-management (GP14)*

In the few instances when GP documentation matched what patients had communicated, patients asserted that the medication was either helping to settle or control bodily sensations, or was having no effect:

Patient: *"I started taking prednisolone… [for colitis flare-up]. Well, it is working. (eye contact, nods head) I've been taking them for a couple of weeks and the condition is improved...(P21: M, 70-79 years)*	Medical records: *ulcerative colitis settled now with use of oral steroids (GP19)*

Domain: The patient's perspective

For peer review only

Effect on life

The effect of patients' bodily sensations on functional and social aspects of their life was discussed on twelve occasions but was rarely documented in medical records. There were no clear language differences between misalignments and alignments.

Ideas and beliefs

Patient's ideas and beliefs were documented on most occasions when patients offered a plausible explanation as to what may be causing their bodily sensations. Patients typically attributed sensations to medication side-effects, flare-up of a pre-existing condition, diet, and stress, which on occasion matched GPs ideas and beliefs. In the one instance that patient's ideas and beliefs were omitted from records, the patient was unable to justify the possible cause.

Concern about what symptoms might mean

Patients rarely expressed concerns about the cause or seriousness of their bodily sensations. In the two instances where patient's concerns were documented in medical records, patients had either relayed their partner's concern about "looking drawn" or explicitly asked the GP whether their bodily sensations could be due to an underlying cancer:

Patient: *"You don't think it's cancerous basically? (shakes head, right hand holding shirt collar)" (P25: F, 50-59 years)*	Medical records: *Very worried about cancer (GP20)*

Patients' concerns were typically not documented when the possibility of a sinister cause was voiced but cancer was not directly articulated:

Patient: *"There's nothing bad really down there? (touches upper abdomen with both hands) […] Because it swells up quite a bit when it happened" (P17: F, 70-79 years)*	Medical records: *nothing recorded in relation to patient concerns (GP14)*

GP and patient characteristics

The frequency of alignments and misalignments for each clinical feature was insufficient for stratifying data to identify GP characteristics (sex, age, years since qualified as a GP) or patient characteristics (sex, age, educational attainment, social deprivation level) as additional sources of GP variation in how abdominal symptoms were documented.

For peer review only - http://bmjopen.bmj.com/site/about/guidelines.xhtml

For peer review only

Discussion

Summary of key findings

A wide range of clinical features pertaining to abdominal and systemic sensations were discussed between patients and GPs during primary care consultations. There was a gap between what patients communicated and the information about these bodily sensations GPs documented in medical records. Vague descriptors, figurative speech, and lengthy explanations of bodily sensations without an exact location, characterised features of patients' history that was not recorded, documented incompletely, or did not match what patients verbalised. Clinical features that were well-defined and succinct with precise gesticulations for abdominal sensations were well documented. Abdominal 'pain' was almost always documented compared to 'discomfort'. 'Discomfort' was conveyed using descriptors that indicated sensations that were unpleasant, whereas 'pain' was communicated as an aversive sensation. Abdominal features were omitted from medical records when large hand gesticulations, which supplemented or superseded verbal descriptions for the location of 'pain' or 'discomfort', were used.

Strengths and limitations

A strength of this work is that our findings are derived from direct comparison of naturalistic data, verified through video observation and medical records. To our knowledge, this is the only study to explore how patients describe undifferentiated symptoms of possible cancer during a consultation and compare this to what is subsequently documented in patients' medical record. Patients in this study communicated a range of abdominal symptoms that UGI cancer patients present with before diagnosis. This affords credible insight into the different ways patients may attempt to convey abdominal symptoms to GPs when presenting to primary care. By structuring our analysis around the 'gathering information' domains of an established communication teaching tool,(37) we were able to characterize nuances in patients' communication of abdominal sensations according to the clinical features doctors are trained to explore during the medical interview and as such are. directly applicable to medical training programs. As abdominal symptoms more often suggest conditions other than cancer, our findings are relevant to health care research concerned with enhancing the information gathered and integrated to safely differentiate serious from non-serious illness.

This study does have some limitations. Our findings reflect the immediately observable aspects of patient and GPs' verbal and non-verbal communication, rather than latent meaning. Thus, misalignments are not synonymous with a poor history as GPs may have

For peer review only - http://bmjopen.bmj.com/site/about/guidelines.xhtml

accurately interpreted patients intended meaning despite literal differences in written documentation.(38) What GPs documented may have been affected by individual characteristics of patients and GPs, patients' agenda, and the structure and relative brevity of medical records in UK general practice, which were beyond the scope of this study.(39) A complete patient history is more than the sum of its parts; parsing abdominal sensations from the patient narrative (as in our approach) potentially negates correct interpretation of the overall clinical picture. As we did not have access to records of patients' prior medical history or final diagnosis, we could not determine whether symptoms documented (or omitted) were truly indicative of serious disease. We therefore identified that there was a difference between what patients communicated and what GPs documented, not whether this difference was clinically important. Our sample was almost exclusively White British and native English speakers so our findings may not be transferable to practices serving patient populations with different characteristics. The focus of this study was limited to GPs. Therefore, our findings have most applicability to GPs in the UK and countries with similar health care structure. However, we anticipate our findings will also be relevant to other primary care providers, such as physician assistants and advanced nursing practitioners seeing patients with new symptoms. This study analyses the content of UK face-to-face consultations before Covid-19 prompted a shift to teleconsulting for triaging new patient problems in primary care.(40) Although face-to-face consultations are gradually resuming, remote consultations remain ubiquitous and may affect how patients communicate and what GPs document. Therefore, our findings are unlikely to reflect communication or documentation practices occurring from remote patient-GP interactions where visual and non-verbal body language are occluded. Finally, data interpretation is subjective and influenced by the epistemic position of the research team; VH is a PhD student with a physiotherapy background where the character of patient's pain is important for ascertaining the involved structure. Consequently, certain patterns in the patient's history may have been more apparent than would have been if approached through a different lens. To ensure confirmability, data interpretation was regularly discussed and revised in collaboration with senior academic GPs (FW, JUS) and PPI representatives (MJ, JL).

Comparison with existing research

As previously mentioned, this is the only study to directly examine how patients describe abdominal sensations with what GPs subsequently document in the context of early cancer diagnosis.

Existing literature has shown that patients with cancer describe abdominal sensations using nuanced vocabulary that extends beyond well-defined medical nomenclature, which they sometimes conflate. Humphrys *et al.* found esophago-gastric cancer patients used terms

For peer review only - http://bmjopen.bmj.com/site/about/guidelines.xhtml

such as “stringy goo” to describe their phlegm and articulated that their legs were “getting thinner” to convey weight loss.(17) In the same study, patients held overlapping conceptualizations of heartburn, dyspepsia and indigestion which were inconsistent with the medical definition of each symptom.(17) For example, patients described heartburn, which is defined by the American Gastroenterological Association as a “burning sensation in the retrosternal area”, in terms of acid reflux or dyspepsia.(17,41) Bankhead *et al.* similarly noted interchangeable use of medical terms in interviews with women with suspected ovarian cancer, where increased abdominal size was referred to as ‘bloating’ instead of the appropriate medical term ‘abdominal distension’.(18) The most comparable study available is a recently published prospective cohort study of over 300 patients, which examined agreement of GP and patient-reported abdominal symptoms at referral for faecal immunochemical testing for suspected colorectal cancer.(19) Considerable variation in agreement was observed by symptom type and symptom severity: GPs most frequently reported abdominal pain and diarrhoea compared to reflux and constipation and increasingly reported abdominal pain and diarrhoea as patient-reported symptom severity on a six-point Likert scale increased (e.g., 63.6% and 53.9% respectively for minor discomfort *vs* 83.3% and 94.4% respectively for very severe discomfort). However, because studies have typically used interviews(17,18) and survey methods(19) for patients referred for suspected cancer or recently diagnosed with cancer, insights yielded may not fully capture how patients communicate bodily sensations to GPs that are yet to prompt suspicion. As we explored how patients communicate abdominal sensations during primary care consultations before diagnosis, our findings extend existing literature by identifying nuances in how patients may convey bodily sensations during earlier consultations. Further, our findings suggest that discrepancies in GPs reporting of abdominal sensations may continue into the medical records depending how they are communicated.

Future research

The finding that well-defined or painful abdominal sensations may be more salient to GPs than vague bodily sensations expressed as ‘discomfort’ warrants further evaluation. Differences between the information patients communicate about their abdominal sensations and what GPs document could be clinically important given the majority of patients with cancer are diagnosed following symptomatic presentation to primary care,(42) and post-hoc case reviews suggest some symptoms are interpreted incorrectly or not acted upon.(43,44) Establishing the importance of discrepancies in medical documentation requires an understanding of the meaning behind the words and phrases patients use, and the extent to which ‘lay’ descriptors can be reconciled with medical nomenclature. Exploring this will be vital for developing strategies that facilitate medical documentation that more closely reflects the patient’s history.

For peer review only - http://bmjopen.bmj.com/site/about/guidelines.xhtml

The influence of identified variations in patients' descriptions of abdominal sensations on GPs subsequent use of tests and referral pathways leading to an UGI cancer diagnosis will also be important to establish. In particular, the impact on the detection of harder-to-diagnose cancers via new rapid diagnostic centres (RDCs) for concerning non-specific symptoms not qualifying for NICE 'fast-track' referral requires consideration.(45) Earlier cancer diagnosis through RDCs is contingent upon prompt recognition of non-specific symptoms needing investigation, which may be hindered if potentially concerning vague symptoms or 'discomfort' are not recognized as important. Gender differences in the way patients describe symptoms of angina has been reported, with male patients using succinct language compared to female patients who used a wider repertoire of descriptors.(46) Thus, variations in descriptions of abdominal sensations by patient gender and other characteristics such as socioeconomic status should be explored given the potential implications for inequalities in the timely investigation of cancer. We identify a range of symptom features (e.g., aggravating and relieving factors and duration) capturing the nature and quality of patients' bodily sensations which are yet to be evaluated in studies developing risk prediction models for early cancer detection that limit inclusion to symptom type.(20,21,47,48) While most patients with cancer are diagnosed after a referral from their GP, the expanding role of nurse practitioners in primary care settings means they are increasingly positioned to encounter patients with concerning symptoms needing investigation.(49) As patients may feel more at ease communicating with nurses than GPs,(50) examining the content of patients' dialogue with nurse practitioners might offer additional insight into how to unpack the patient history.

Implications for policy and practice

Abdominal sensations described in well-defined terms or as 'pain' may be deemed more significant by GPs than those communicated as 'discomfort' or described more vaguely, even though bodily sensations by are subjectively experienced states.(14) This could mean that patients more able to present their abdominal sensations succinctly or in terminology akin to medical terminology may be perceived to have more concerning symptoms which are investigated more promptly than patients with serious illness who do not articulate their symptoms as definitively. The integrity of current and future risk prediction models for UGI cancer (and other cancers of the abdomen)(20,21,51,52) which harvest coded and/or free-text symptoms from medical records may be undermined if consultation notes do not reflect the patient's history. Similarly, growing use of 'chatbot' or App technologies which utilize symptoms entered remotely by patients to suggest clinical management may be flawed if information about abdominal symptoms relies solely on medical terminology.(53)

For peer review only - http://bmjopen.bmj.com/site/about/guidelines.xhtml

GPs need to be cognizant that patients describe abdominal sensations in a variety of ways that differ from medical terminology which may not be indicative of their significance. Policy makers, and researchers and engineers in research and development should avoid interventions or guidance that reinforce false dichotomies that give precedence to abdominal symptoms packaged in medical language over 'lay' expressions or 'discomfort'. Instead, strategies that support GPs to interpret and act on patient meaning across a broader range of abdominal features rather than clinically exacting terminology is needed.

Funding

This work is supported by the CanTest Collaborative which is funded by Cancer Research UK (C8640/A23385), where VH is a PhD student, FMW is Director, and MJT, HS, and JE are Associate Directors. SA is supported by Cancer Research UK grants (C12292/A20861). The Bristol Archive Project was funded by the National Institute for Health Research (NIHR) School for Primary Care Research (208) and the South West GP Trust.

Contributions

VH and FW designed the study. RKB collected the primary data upon which this analysis was conducted. VH led the analysis and interpretation of the data with support from JUS, SA, and FW. VH drafted the manuscript. JUS, SA, FW, RKB, JL, MJ, MT, JE, HS, FMW critically reviewed and commented on the manuscript. All authors approved the final manuscript for submission.

Competing interests

None declared.

Data availability statement

No data are available.

References

1. Mulley AG, Trimble G, Elwyn Glyn. Stop the silent misdiagnosis: patients' preferences matter. *BMJ*. 2012;345:e6572. doi:10.1136/bmj.e6572
2. Committee on Diagnostic Error in Health Care; Institute of Medicine; The National Academies of Sciences, Engineering, and Medicine. Improving Diagnosis in Health Care. Balogh EP, Miller BT, Ball JR, editors. National Academies Press (US). 2015; Dec 29:1–472.
3. Gillard S, Benson J, Silverman J. Teaching and assessment of explanation and planning in medical schools in the United Kingdom: Cross sectional questionnaire survey. *Med Teach*. 2009;31(4):328–31.
4. Zwaan L, Thijs A, Wagner C, Timmermans DRM. Does inappropriate selectivity in

For peer review only

information use relate to diagnostic errors and patient harm? The diagnosis of patients with dyspnea. *Soc Sci Med.* 2013;91:32–8.
5. Singh H, Giardina TD, Meyer AND, Forjuoh SN, Reis MD, Thomas EJ. Types and origins of diagnostic errors in primary care settings. *JAMA Intern Med.* 2013;173(6):418–25.
6. Gruppen LD, Wolf FM, Billi JE. Information gathering and integration as sources of error in diagnostic decision making. *Med Decis Mak.* 1991;11(4):233–9.
7. Koo MM, von Wagner C, Abel GA, McPhail S, Hamilton W, Rubin GP, et al. The nature and frequency of abdominal symptoms in cancer patients and their associations with time to help-seeking: evidence from a national audit of cancer diagnosis. *J Public Health.* 2018 Sep 1;40(3):e388–95. doi: 10.1093/pubmed/fdx188.
8. Rasmussen S, Haastrup PF, Balasubramaniam K, Christensen R, Sondergaard Jens; Jarbol DE. Predictive values of upper gastrointestinal cancer alarm symptoms in the general population: a nationwide cohort study. *BMC Cancer.* 2018;18(440). doi:10.1186/s12885-018-4376-8.
9. National Institute for Health and Care Excellence. Gastrointestinal tract (upper) cancers - recognition and referral NG12. Available online: https://cks.nice.org.uk/topics/gastrointestinal-tract-upper-cancers-recognition-referral/ (accessed on 3 August 2021).
10. Calanzani N, Druce PE, Snudden C, Milley KM, Boscott R, Behiyat D, et al. Identifying Novel Biomarkers Ready for Evaluation in Low-Prevalence Populations for the Early Detection of Upper Gastrointestinal Cancers: A Systematic Review. *Adv Ther.* 2020; 38:793–834.
11. National Cancer Intelligence Network. Routes to Diagnosis 2006-2016 by Year. Available online: http://www.ncin.org.uk/publications/routes_to_diagnosis (accessed on 15 January 2021).
12. Fitzgerald RC, di Pietro M, O'Donovan M, Maroni R, Muldrew B, Debiram-Beecham I, et al. Cytosponge-trefoil factor 3 versus usual care to identify Barrett's oesophagus in a primary care setting: a multicentre, pragmatic, randomised controlled trial. *Lancet.* 2020;396(10247):333–44.
13. Malterud K, Guassora AD, Graungaard AH, Reventlow S. Understanding medical symptoms: a conceptual review and analysis. *Theor Med Bioeth.* 2015;36(6):411–24..
14. Rosendal M, Jarbøl DE, Pedersen AF, Andersen RS. Multiple perspectives on symptom interpretation in primary care research. *BMC Fam Pract.* 2013;14:167.
15. Sonnenberg A, Gogel HK. Translating vague complaints into precise symptoms : the implications of a poor medical history. *Eur J Gastroenterol Hepatol.* 2002;14(3):317–21.
16. Mills K, Birt L, Emery JD, Hall N, Banks J, Johnson M, et al. Understanding symptom appraisal and help-seeking in people with symptoms suggestive of pancreatic cancer: A qualitative study. *BMJ Open.* 2017;7(9):1–9.
17. Humphrys E, Walter FM, Rubin G, Emery JD, Johnson M, Richards A, et al. Patient symptom experience prior to a diagnosis of oesophageal or gastric cancer: A multi-

For peer review only - http://bmjopen.bmj.com/site/about/guidelines.xhtml

methods study. *BJGP Open*. 2020;4(1):1–15.
18. Bankhead CR, Collins C, Stokes-Lampard H, Rose P, Wilson S, Clements A, et al. Identifying symptoms of ovarian cancer: A qualitative and quantitative study. BJOG *An Int J Obstet Gynaecol*. 2008;115(8):1008–14.
19. Högberg C, Karling P, Rutegård J, Lilja M. Patient-reported and doctor-reported symptoms when faecal immunochemical tests are requested in primary care in the diagnosis of colorectal cancer and inflammatory bowel disease: A prospective study. *BMC Fam Pract*. 2020;21(1):1–12.
20. Stapley S, Peters TJ, Neal RD, Rose PW, Walter FM, Hamilton W. The risk of pancreatic cancer in symptomatic patients in primary care: A large case-control study using electronic records. *Br J Cancer*. 2012;106(12):1940–4.
21. Stapley S, Peters TJ, Neal RD, Rose PW, Walter FM, Hamilton W. The risk of oesophago-gastric cancer in symptomatic patients in primary care: A large case-control study using electronic records. *Br J Cancer*. 2013;108(1):25–31.
22. Shephard E, Neal R, Rose P, Walter F, Hamilton WT. Clinical features of kidney cancer in primary care: A case-control study using primary care records. *Br J Gen Pract*. 2013;63(609):250–5.
23. Shephard EA, Hamilton W, Neal RD, Rose PW, Walter FM. Symptoms of adult chronic and acute leukaemia before diagnosis: Large primary care case-control studies using electronic records. *Br J Gen Pract*. 2016;66(644):e182–8. doi: 10.3399/bjgp16X683989.
24. Tate AR, Martin AGR, Ali A, Cassell JA. Using free text information to explore how and when GPs code a diagnosis of ovarian cancer: An observational study using primary care records of patients with ovarian cancer. *BMJ Open*. 2011;1(1):1–7.
25. Koleck TA, Dreisbach C, Bourne PE, Bakken S. Natural language processing of symptoms documented in free-text narratives of electronic health records: A systematic review. *J Am Med Informatics Assoc*. 2019;26(4):364–79.
26. Edwards PJ, Bennett-Britton I, Ridd MJ, Booker M, Barnes RK. Factors affecting the documentation of spoken safety-netting advice in routine GP consultations: a cross-sectional study. *Br J Gen Pract*. 2021;71(712):e869-e876. doi: 10.3399/BJGP.2021.0195.2021.0195.
27. Weiner SJ, Wang S, Kelly B, Sharma G, Schwartz A. How accurate is the medical record? A comparison of the physician's note with a concealed audio recording in unannounced standardized patient encounters. *J Am Med Informatics Assoc*. 2020;27(5):770–5.
28. Barnes RK. One in a Million: A study of primary care consultations. Bristol: University of Bristol, 2017. Available online: https://doi.org/10.5523/bris.l3sq4s0w66ln1x20sye7s47wv (accessed on 3 August 2021).
29. Jepson M, Salisbury C, Ridd MJ, Metcalfe C, Garside L, Barnes RK. The "One in a Million" study: Creating a database of UK primary care consultations. *Br J Gen Pract*. 2017;67(658):e345–51.
30. Din NU, Ukoumunne OC, Rubin G, Hamilton W, Carter B, Stapley S, Neal RD. Age

For peer review only - http://bmjopen.bmj.com/site/about/guidelines.xhtml

and Gender Variations in Cancer Diagnostic Intervals in 15 Cancers: Analysis of Data from the UK Clinical Practice Research Datalink. *PLoS One*. 2015;10(5): e0127717. doi: 10.1371/journal.pone.0127717.
31. Erlingsson C, Brysiewicz P. A hands-on guide to doing content analysis. African J *Emerg Med*. 2017;7(3):93–9.
32. Hsieh HF, Shannon SE. Three approaches to qualitative content analysis. *Qual Health Res*. 2005;15(9):1277–88.
33. Bengtsson M. How to plan and perform a qualitative study using content analysis. *NursingPlus Open*. 2016;2:8–14.
34. Graneheim UH, Lindgren BM, Lundman B. Methodological challenges in qualitative content analysis: A discussion paper. *Nurse Educ Today*. 2017;56(May):29–34.
35. Silverman J, Kurtz S, Draper J. Skills for communicating with patients. 3rd edition. CRC Press Taylor & Francis Group. 2013. p69–130.
36. Procter S, Stewart K, Reeves D, Bowen L, Purdy S, Ridd M, et al. Complex consultations in primary care: A tool for assessing the range of health problems and issues addressed in general practice consultations. *BMC Fam Pract*. 2014;15(1):1–8.
37. Kurtz SM, Silverman JD. The Calgary-Cambridge Referenced Observation Guides: An aid to defining the curriculum and organizing the teaching in communication training programmes. *Med Educ*. 1996;30(2):83–9.
38. Amelung D, Whitaker KL, Lennard D, Ogden M, Sheringham J, Zhou Y, et al. Influence of doctor-patient conversations on behaviours of patients presenting to primary care with new or persistent symptoms: A video observation study. *BMJ Qual Saf*. 2020;29(3):198–208.
39. Colicchio TK, Cimino JJ. Clinicians' reasoning as reflected in electronic clinical note-entry and reading/retrieval: a systematic review and qualitative synthesis. *J Am Med Informatics Assoc*. 2019;26(2):172–84.
40. Mann C, Turner A, Salisbury C. The impact of remote consultations on personalised care Evidence briefing Commissioned by the Personalised Care Institute. *Natl Inst Heal Res*. 2021;(May):3–20.
41. Vakil N, Van Zanten S V., Kahrilas P, Dent J, Jones R, Bianchi LK, et al. The Montreal definition and classification of gastroesophageal reflux disease: A global evidence-based consensus. *Am J Gastroenterol*. 2006;101(8):1900-20.
42. Elliss-Brookes L, McPhail S, Ives A, Greenslade M, Shelton J, Hiom S, et al. Routes to diagnosis for cancer - Determining the patient journey using multiple routine data sets. *Br J Cancer*. 2012;107(8):1220–6.
43. Singh H, Davis Giardina T, Petersen LA, Smith M, Wilson L, Dismukes K, et al. Exploring Situational Awareness in Diagnostic Errors in Primary Care Conclusions— A framework of SA can help analyze and understand diagnostic errors in primary care settings that use EHRs. *BMJ Qual Saf*. 2012;21(110):30–8.
44. Lyratzopoulos G, Vedsted P, Singh H. Understanding missed opportunities for more timely diagnosis of cancer in symptomatic patients after presentation. *Br J Cancer*. 2015;112:S84–91. doi:10.1038/bjc.2015.47
45. Chapman D, Poirier V, Vulkan D, Fitzgerald K, Rubin G, Hamilton W, et al. First

For peer review only

For peer review only - http://bmjopen.bmj.com/site/about/guidelines.xhtml

results from five multidisciplinary diagnostic centre (MDC) projects for non-specific but concerning symptoms, possibly indicative of cancer. *Br J Cancer.* 2020;123:722–729.
46. Kreatsoulas C, Crea-Arsenio M, Shannon HS, Velianou JL, Giacomini M. Interpreting angina: symptoms along a gender continuum. *Open Heart.* 2016;3(1):e000376.
47. Lyratzopoulos G, Abel GA. Assessing patients at risk of symptomatic-but-as-yet-undiagnosed cancer in primary care using information from patient records. *Br J Cancer.* 2020;122(12):1729–31.
48. Koshiaris C, den Bruel A Van, Nicholson BD, Lay-Flurrie S, Richard Hobbs FD, Oke JL. Clinical prediction tools to identify patients at highest risk of myeloma in primary care: a retrospective open cohort study. *Br J Gen Pract.* 2021;71(706):E347–55.
49. Skrobanski H, Ream E, Poole K, Whitaker KL. Understanding primary care nurses' contribution to cancer early diagnosis: A systematic review. *Eur J Oncol Nurs.* 2019;41:149–64.
50. Cromme SK, Whitaker KL, Winstanley K, Renzi C, Smith CF, Wardle J. Worrying about wasting GP time as a barrier to help-seeking: A community-based, qualitative study. *Br J Gen Pract.* 2016;66(648):e474–82. doi:10.3399/bjgp16X685621.
51. Hippisley-Cox J, Coupland C. Identifying patients with suspected pancreatic cancer in primary care: Derivation and validation of an algorithm. *Br J Gen Pract.* 2012;62(594):38–45.
52. Hippisley-Cox, Julia and Coupland C. Identifying patients with suspected gastro-oesophageal cancer in primary care: derivation and validation of an algorithm. *Br J Gen Pract.* 2011;61(592):e707-14. doi: 10.3399/bjgp11X606609.
53. 98point6. On-demand, text-based primary care [Internet]. [cited 2021 Oct 6]. Available from: https://www.98point6.com/

For peer review only - http://bmjopen.bmj.com/site/about/guidelines.xhtml

Figure 1. Flow diagram of included consultations

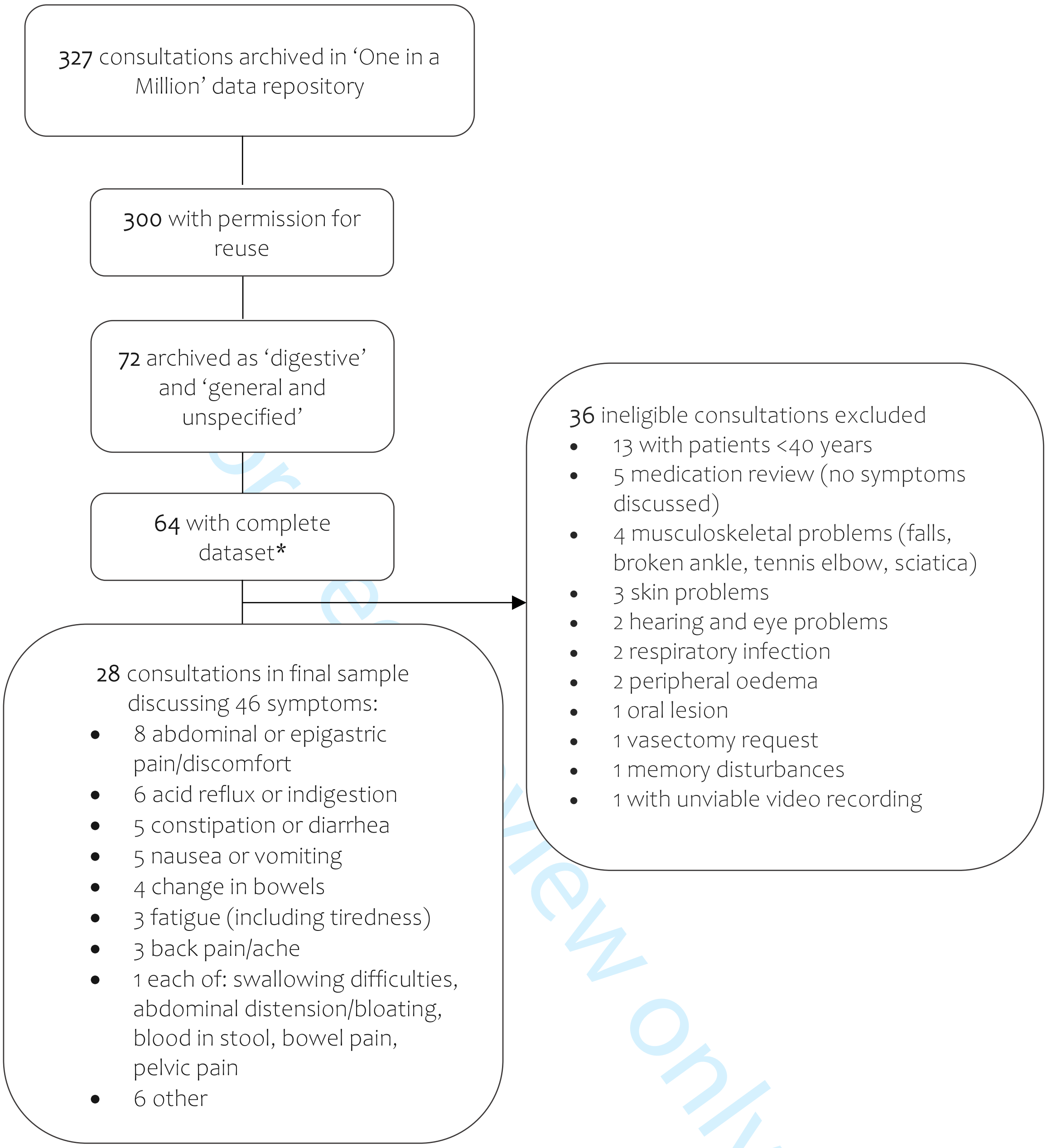

*Video recording, transcript, and medical record entry

Figure 2. Final categories representing the clinical features of abdominal sensations discussed in primary care consultations based on the 'gathering information' domains of the Calgary-Cambridge model

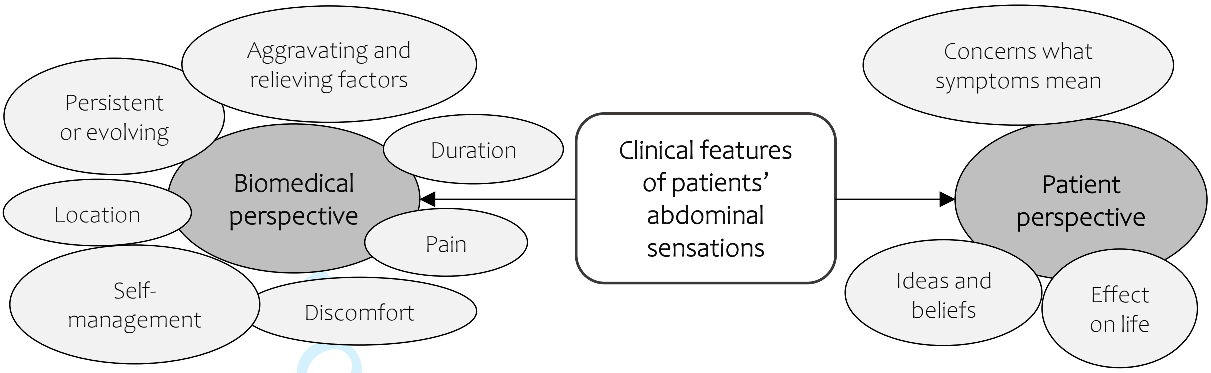

For peer review only

Figure 3. Frequency of alignments and misalignments for each feature of patients' abdominal and systemic bodily sensations, according to the 'gathering information' domain of the Calgary-Cambridge Guide

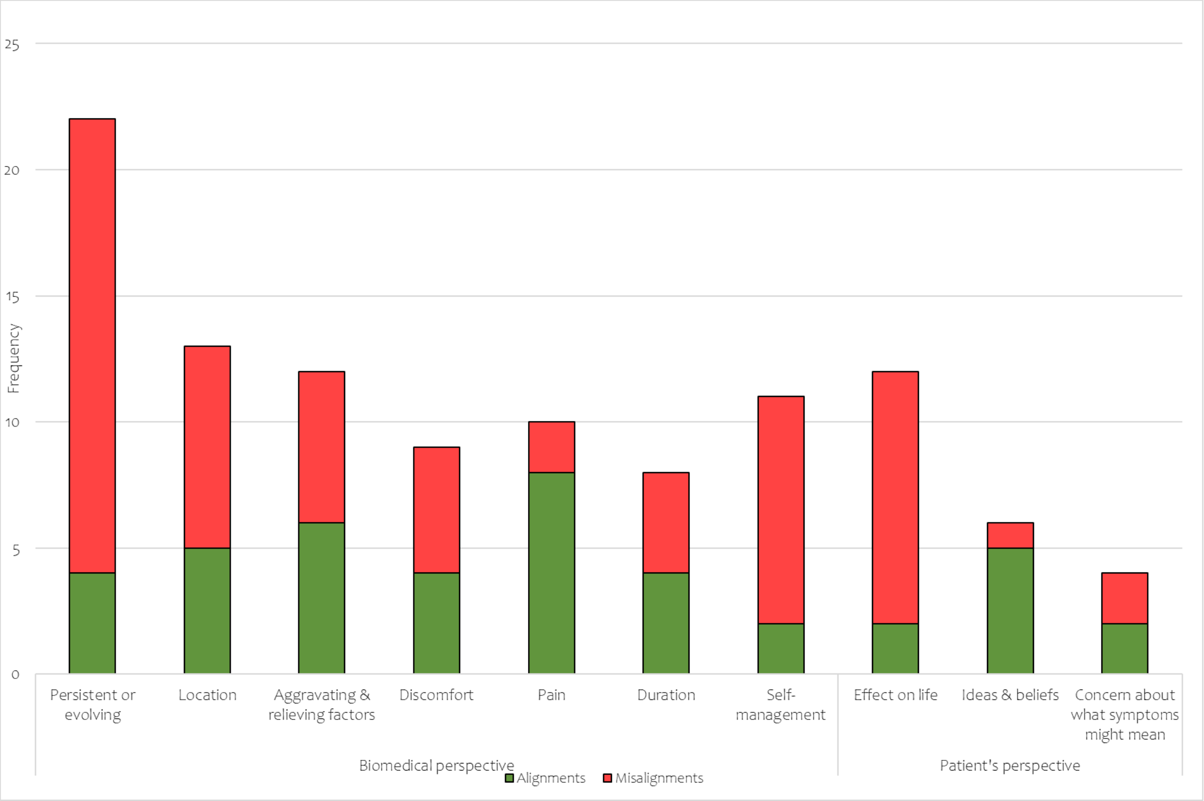

Supplementary file A. Details of included patients and their upper GI/systemic bodily sensations

Patient ID	Gender	Age	Bodily sensations
1	Female	66	Follow-up post-emergency operation for strangulated hernia, 'sticky bowels'
2	Female	71	Nausea, acid reflux
3	Female	72	Abdominal and pelvic pain, back ache
4	Male	55	Abdominal pain
5	Female	65	Nausea
6	Female	79	Heartburn, nausea
7	Female	73	Abdominal pain
8	Male	65	Abdominal and rectal pain, loose stools
9	Male	60	Acid reflux, indigestion
10	Male	59	Tiredness, headaches
11	Female	69	Abdominal distension/bloated
12	Male	73	Bowel pain, constipation
13	Male	83	Swallowing difficulties
14	Female	58	Back pain
15	Female	45	Fatigue, difficulty urinating
16	Male	81	Constipation
17	Female	77	Epigastric and back pain, nausea and vomiting
18	Female	53	Abdominal pain, nausea, diarrhoea, blood in stools
19	Female	42	Heartburn
20	Female	72	Reflux
21	Male	71	Loose stools, colitis flare-up
22	Female	41	Change in bowels, low haemoglobin
23	Female	70	Abdominal pain, change in bowels
24	Male	43	Fatigue, change in bowels
25	Female	59	Voice hoarseness, stomach problems
26	Male	74	Hernia
27	Male	64	Gastritis, follow-up of biopsy reversal
28	Female	53	Epigastric discomfort

For peer review only

COREQ (COnsolidated criteria for REporting Qualitative research) Checklist

A checklist of items that should be included in reports of qualitative research. You must report the page number in your manuscript where you consider each of the items listed in this checklist. If you have not included this information, either revise your manuscript accordingly before submitting or note N/A.

Topic	Item No.	Guide Questions/Description	Reported on Page No.
**Domain 1: Research team and reflexivity**			
Personal characteristics			
Interviewer/facilitator	1	Which author/s conducted the interview or focus group?	N/A
Credentials	2	What were the researcher's credentials? E.g. PhD, MD	15-16
Occupation	3	What was their occupation at the time of the study?	15-16
Gender	4	Was the researcher male or female?	N/A
Experience and training	5	What experience or training did the researcher have?	16
Relationship with participants			
Relationship established	6	Was a relationship established prior to study commencement?	N/A
Participant knowledge of the interviewer	7	What did the participants know about the researcher? e.g. personal goals, reasons for doing the research	N/A
Interviewer characteristics	8	What characteristics were reported about the inter viewer/facilitator? e.g. Bias, assumptions, reasons and interests in the research topic	N/A
**Domain 2: Study design**			
Theoretical framework			
Methodological orientation and Theory	9	What methodological orientation was stated to underpin the study? e.g. grounded theory, discourse analysis, ethnography, phenomenology, content analysis	2, 5
Participant selection			
Sampling	10	How were participants selected? e.g. purposive, convenience, consecutive, snowball	6
Method of approach	11	How were participants approached? e.g. face-to-face, telephone, mail, email	5, 6
Sample size	12	How many participants were in the study?	2, 7, 8
Non-participation	13	How many people refused to participate or dropped out? Reasons?	N/A
Setting			
Setting of data collection	14	Where was the data collected? e.g. home, clinic, workplace	5
Presence of non-participants	15	Was anyone else present besides the participants and researchers?	5
Description of sample	16	What are the important characteristics of the sample? e.g. demographic data, date	8
Data collection			
Interview guide	17	Were questions, prompts, guides provided by the authors? Was it pilot tested?	N/A
Repeat interviews	18	Were repeat inter views carried out? If yes, how many?	N/A
Audio/visual recording	19	Did the research use audio or visual recording to collect the data?	2, 5
Field notes	20	Were field notes made during and/or after the inter view or focus group?	6, 7
Duration	21	What was the duration of the inter views or focus group?	N/A
Data saturation	22	Was data saturation discussed?	N/A
Transcripts returned	23	Were transcripts returned to participants for comment and/or	N/A

For peer review only

Topic	Item No.	Guide Questions/Description	Reported on Page No.
		correction?	
**Domain 3: analysis and findings**			
Data analysis			
Number of data coders	24	How many data coders coded the data?	6-7
Description of the coding tree	25	Did authors provide a description of the coding tree?	6-7
Derivation of themes	26	Were themes identified in advance or derived from the data?	6-7
Software	27	What software, if applicable, was used to manage the data?	6-7
Participant checking	28	Did participants provide feedback on the findings?	15
Reporting			
Quotations presented	29	Were participant quotations presented to illustrate the themes/findings? Was each quotation identified? e.g. participant number	11-14
Data and findings consistent	30	Was there consistency between the data presented and the findings?	11-14
Clarity of major themes	31	Were major themes clearly presented in the findings?	11-14
Clarity of minor themes	32	Is there a description of diverse cases or discussion of minor themes?	11-14

Developed from: Tong A, Sainsbury P, Craig J. Consolidated criteria for reporting qualitative research (COREQ): a 32-item checklist for interviews and focus groups. *International Journal for Quality in Health Care*. 2007. Volume 19, Number 6: pp. 349 – 357

Once you have completed this checklist, please save a copy and upload it as part of your submission. DO NOT include this checklist as part of the main manuscript document. It must be uploaded as a separate file.

For peer review only

BMJ Open

Agreement between patient's description of abdominal symptoms of possible upper gastrointestinal cancer and General Practitioner consultation notes: a qualitative analysis of video-recorded UK primary care consultation data

Journal:	*BMJ Open*
Manuscript ID	bmjopen-2021-058766.R2
Article Type:	Original research
Date Submitted by the Author:	15-Aug-2022

Complete List of Authors:	Hardy, Victoria; University of Cambridge, Public Health and Primary Care<br>Usher-Smith, Juliet; University of Cambridge, Department of Public Health and Primary Care<br>Archer, Stephanie; University of Cambridge, Department of Public Health and Primary Care<br>Barnes, Rebecca ; University of Oxford Nuffield Department of Primary Care Health Sciences<br>Lancaster, John; University of Cambridge, Patient Representative c/o Public Health & Primary Care<br>Johnson, Margaret ; University of Cambridge, Patient Representative c/o Public Health & Primary Care<br>Thompson, Matthew; University of Washington, Department of Family Medicine<br>Emery, Jon; University of Melbourne Victorian Comprehensive Cancer Centre, Centre for Cancer Research and General Practice and Primary Care Academic Centre<br>Singh, Hardeep; Michael E DeBakey VA Medical Center, Houston Veterans Affairs Center for Innovations in Quality, Effectiveness and Safety; Baylor College of Medicine, Department of Medicine<br>Walter, Fiona; Queen Mary University of London, Wolfson Institute of Population Health, Barts and the London School of Medicine and Dentistry; University of Cambridge, Department of Public Health and Primary Care

**Primary Subject Heading**:	General practice / Family practice
Secondary Subject Heading:	Qualitative research, Evidence based practice, Medical education and training
Keywords:	PRIMARY CARE, QUALITATIVE RESEARCH, MEDICAL EDUCATION & TRAINING

SCHOLARONE™ Manuscripts

I, the Submitting Author has the right to grant and does grant on behalf of all authors of the Work (as defined in the below author licence), an exclusive licence and/or a non-exclusive licence for contributions from authors who are: i) UK Crown employees; ii) where BMJ has agreed a CC-BY licence shall apply, and/or iii) in accordance with the terms applicable for US Federal Government officers or employees acting as part of their official duties; on a worldwide, perpetual, irrevocable, royalty-free basis to BMJ Publishing Group Ltd ("BMJ") its licensees and where the relevant Journal is co-owned by BMJ to the co-owners of the Journal, to publish the Work in this journal and any other BMJ products and to exploit all rights, as set out in our licence.

The Submitting Author accepts and understands that any supply made under these terms is made by BMJ to the Submitting Author unless you are acting as an employee on behalf of your employer or a postgraduate student of an affiliated institution which is paying any applicable article publishing charge ("APC") for Open Access articles. Where the Submitting Author wishes to make the Work available on an Open Access basis (and intends to pay the relevant APC), the terms of reuse of such Open Access shall be governed by a Creative Commons licence – details of these licences and which Creative Commons licence will apply to this Work are set out in our licence referred to above.

Other than as permitted in any relevant BMJ Author's Self Archiving Policies, I confirm this Work has not been accepted for publication elsewhere, is not being considered for publication elsewhere and does not duplicate material already published. I confirm all authors consent to publication of this Work and authorise the granting of this licence.

For peer review only

Agreement between patient's description of abdominal symptoms of possible upper gastrointestinal cancer and General Practitioner consultation notes: a qualitative analysis of video-recorded UK primary care consultation data

Victoria Hardy[1], Juliet Usher-Smith[1], Stephanie Archer[1], Rebecca K. Barnes[2], John Lancaster[1], Margaret Johnson[1], Matthew Thompson[3], Jon Emery[4], Hardeep Singh[5], Fiona M. Walter[6,1]

Corresponding author

Victoria Hardy, Department of Public Health & Primary Care, University of Cambridge, Cambridge, UK

veh29@medschl.cam.ac.uk

Affiliations

[1]Department of Public Health & Primary Care, University of Cambridge, Cambridge, UK

[2]Nuffield Department of Primary Care Health Sciences, University of Oxford, Oxford, UK

[3]Department of Family Medicine, University of Washington, Seattle, Washington, USA

[4]Centre for Cancer Research and Department of General Practice, University of Melbourne, Victorian Comprehensive Cancer Centre, Melbourne, Victoria, Australia

[5]Center for Innovations in Quality, Effectiveness and Safety, Michael E. DeBakey VA Medical Center and Baylor College of Medicine, Houston, TX, USA.

[6]Wolfson Institute of Population Health, Barts and the London School of Medicine and Dentistry, Queen Mary University of London, UK

Key words: primary care, symptoms, diagnosis, qualitative research

Word count: 4493

For peer review only - http://bmjopen.bmj.com/site/about/guidelines.xhtml

Abstract

Introduction

Abdominal symptoms are common in primary care but infrequently might be due to an upper gastrointestinal (UGI) cancer. Patients' descriptions may differ from medical terminology used by General Practitioners (GPs). This may affect how information about abdominal symptoms possibly due to an UGI cancer are documented, creating potential missed opportunities for timely investigation.

Objectives

To explore how abdominal symptoms are communicated during primary care consultations, and identify characteristics of patients' descriptions that underpin variation in the accuracy and completeness with which they are documented in medical records.

Methods and analysis

Primary care consultation video recordings, transcripts and medical records from an existing dataset were screened for adults reporting abdominal symptoms. We conducted a qualitative content analysis to capture alignments (medical record entries matching patient verbal and non-verbal descriptions) and misalignments (symptom information omitted or differing from patient descriptions). Categories were informed by the Calgary-Cambridge Guide's 'gathering information' domains and patterns in descriptions explored.

Results

Our sample included 28 consultations (28 patients with 18 GPs): ten categories of different clinical features of abdominal symptoms were discussed. The information GPs documented about these features commonly did not match what patients described, with misalignments more common than alignments (77 vs 44 instances, respectively). Misalignments often featured patients using vague descriptors, figurative speech, lengthy explanations and broad hand gestures. Alignments were characterised by patients using well-defined terms, succinct descriptions and precise gestures for symptoms with an exact location. Abdominal sensations reported as 'pain' were almost always documented compared to expressions of 'discomfort'.

Conclusions

Abdominal symptoms that are well-defined or communicated as 'pain' may be more salient to GPs than those expressed vaguely or as 'discomfort'. Variable documentation of abdominal symptoms in medical records may have implications for the development of clinical decision support systems and decisions to investigate possible UGI cancer.

For peer review only - http://bmjopen.bmj.com/site/about/guidelines.xhtml

ARTICLE SUMMARY

Strengths and limitations

- This is the first study to explore similarities and differences between how patients describe and GPs document abdominal symptoms that could be due to an UGI cancer using naturalistic UK primary care consultation data
- We did not have access to patients' previous consultation history and so could not determine the clinical importance of symptom information that was either not documented in medical records, or did not reflect the patient's history.
- As our sample was mostly White British and native English speakers, patterns in how patients described and GPs documented abdominal symptoms may not be transferable to practices serving patient populations with different characteristics

For peer review only

Introduction

Sir William Osler, lauded as the father of modern medicine, implored his medical students to *"listen to your patient: he is telling you the diagnosis"*.(1) Over a century later, this adage remains germane. As depicted in the National Academy of Medicine's (NAM) conceptual model of the diagnostic process, an accurate and timely diagnosis is predicated on clinicians collecting sufficient data from the patient history.(2) Communication skills teaching is integral to medical training,(3) but suboptimal information gathering is common during the medical interview(4–6) and has been implicated in diagnostic error in primary care settings.(5)

Abdominal symptoms are common in patients presenting to primary care.(7) While such symptoms far more frequently herald benign conditions,(8) they are also described by patients diagnosed with upper gastrointestinal (UGI) cancers (i.e., gastric, oesophageal, and pancreatic), as well as other cancers within the abdomen.(7) In the United Kingdom (UK), compared with 'alarm' symptoms which have relatively high positive predictive value (PPV), abdominal symptoms alone do not meet the 3% PPV threshold recommended by the National Institute for Clinical Excellence (NICE) NG12 (2015) for a 'fast-track' suspected cancer referral.(9) Compounded by a lack of screening programmes or biomarkers reliably raising suspicion of UGI cancer in low prevalence populations,(10) patients with UGI cancers are disproportionately burdened with lengthy diagnostic intervals and late-stage diagnosis, associated with worse survival and patient experiences of healthcare.(11) While the novel 'CytoSponge' test (for Barrett's oesophagus, a precursor lesion for oesophageal cancer(12)) is a promising development for early detection, effective triage of abdominal symptoms for a timely UGI cancer diagnosis, remains currently largely contingent upon the patient history.

Symptoms are subjectively experienced changes in bodily sensations that reflect a perceived departure from the body's "normal" state.(13,14) Patients' experiences of bodily changes are the convergence of sociocultural(13) and experiential factors,(14) and may be communicated differently from standardised medical terminology used by general practitioners (GPs).(15) Studies of UGI cancer patients' symptom appraisal have shown they use a wide range of words and phrases to conceptualise abdominal changes(16,17) that

For peer review only - http://bmjopen.bmj.com/site/about/guidelines.xhtml

are inconsistent with medical definitions familiar to GPs.(17,18) Without a common language, potentially significant bodily sensations risk being overlooked or misinterpreted, and the clinical picture distorted as 'lay' descriptions are translated into equivalent medical nomenclature.(15) Consequently, what GPs document in medical records may not be a complete or accurate representation of the patient's history,(19) potentially creating opportunities for diagnostic delay.

As well as being important at the patient-level for subsequent management decisions, the ways GPs (and other equivalent primary care providers in jurisdictions outside the UK) document patients' bodily sensations in medical records is important for population approaches to early cancer detection. Initiatives in the UK currently harvest coded symptom data from primary care records to develop national guidelines and prediction tools that help GPs estimate patients' current or future cancer risk.(20–23) However, there is mounting interest in harnessing Natural Language Processing techniques to incorporate free-text symptoms.(24,25) Determining how patients describe abdominal sensations to GPs and the extent to which medical records reflect the patient's history is paramount for ensuring clinical decisions tools for UGI (and other) cancers facilitate effective use of tests and referral pathways.

To address this research need, we compared video-recorded primary care consultations featuring new or ongoing abdominal sensations that could be due to an UGI cancer with medical record documentation and explored similarities and differences between patient descriptions of bodily sensations and the way GPs document these as symptoms. We aimed to explore how abdominal symptoms are recorded in the medical record to identify the characteristics of patients' descriptions underpinning GP variation in the medical record entries. We also consider the implications of this on opportunities for earlier diagnosis of UGI cancers. Although other healthcare practitioners in primary care play an important role in the early recognition of symptoms of possible cancer, this study builds on existing observational research that has examined GP's appraisal (26) and documentation of patient's symptoms.(27)

Methods

Design

A cross-sectional descriptive study of video-recorded primary care consultations with linked medical record data entries from the *"One in a Million"* primary care consultation archive, analyzed using qualitative content analysis.

Data source

For peer review only - http://bmjopen.bmj.com/site/about/guidelines.xhtml

The *"One in a Million"* dataset was derived from the 'Bristol Archive project,' a prospective observational cohort study that sought to create a repository of routine primary care consultation data available for use in future research.(28) Between July 2014 and April 2015, twelve purposively sampled general practices in the West of England recorded unselected consultations with adult patients (aged 18 years and above) who presented to primary care over two to three half days.(29) A total of 327 consultations were recorded with linked patient, GP and practice demographic data, and available medical record entries. 89.8% (n=300) of patients consented to selected datasets being re-used in future research, subject to specific ethical approval. All these recordings were anonymized for spoken identifiers, transcribed verbatim and coded for their content according to sixteen "problem type(s)" based on the International Classification of Primary Care – 2nd Edition (ICPC-2) classification scheme.(29) Consultations were assigned more than one code if multiple problems were discussed. We requested all available data for 84 consultations archived as 'digestive' or 'general and unspecified'(29) to yield data directly relevant for addressing our research question.

Eligibility criteria and assessment

We included consultations if there was: a) an accompanying videorecording, transcript, and medical record entry, and b) communication of any new or ongoing abdominal and systemic sensations that could reflect an underlying UGI cancer. We limited inclusion to patients aged 40 years and above, because UGI cancers below this age are rare.(30) Consultations of patients known to have a previous or current cancer diagnosis based on information disclosed during the video-recording were excluded, as a history of cancer provokes more suspicion of serious illness among GPs and patients, and we were interested in GPs' appraisal of patient's bodily sensations in an as-yet undiagnosed population. We also excluded consultations during which no relevant bodily sensations were communicated. This was determined by abstracting all bodily sensations discussed in consultations into a pre-prepared Microsoft Excel form and assessing each as 'include', 'exclude', or 'unsure'. Eligibility assessment was initially undertaken by VH (a non-clinical PhD student) before final adjudication of relevant bodily sensations by FW (a senior academic GP). Consultations for which there were no relevant bodily sensations were excluded at this juncture.

Sampling

As this study was an analysis of a pre-existing archived dataset and no further data collection was possible, we included all consultations of symptomatic patients that met study eligibility criteria.

For peer review only - http://bmjopen.bmj.com/site/about/guidelines.xhtml

Data analysis

Data were analysed using a manifest qualitative content analysis, following the four phases outlined by Erlingsson *et al.*: 'familiarising oneself with the data', 'dividing and condensing the text into meaning units', 'formulating codes', and 'developing categories and themes'.(31) A manifest qualitative content analysis was selected as it is the most suitable analytical technique for exploring and describing visible and explicit patterns (as opposed to the underlying meaning of text as in latent content analysis) across data sources that incorporate visual and textual communication data.(32–34) Data analysis was conducted in excel by VH, with analysis facilitated by SA (senior non-clinical researcher), and data interpretation verified by FW, JUS (senior academic GPs), and PPI (JL, MJ).

We employed a mixed deductive and inductive approach whereby the analytical framework was informed by the 'gathering information' domains of the Calgary-Cambridge Guide. We selected the Calgary-Cambridge Guide over other possible instruments (e.g., the Roter Interaction Analysis System) as it is an evidence-based teaching and assessment resource that is used in medical training programmes internationally to develop doctors' verbal and non-verbal communication skills and structure consultations. The guide delineates the consultation into distinct phases, including 'initiating the session', 'gathering information', 'physical examination', 'explanation and planning', and 'closing the session'. The 'gathering information' component guides doctors through the collection of symptom information relating to the biomedical perspective, patient perspective, and background information that should be elicited during the patient history. Thus, the Calgary-Cambridge Guide offers an ecologically valid framework for guiding the deconstruction and analysis of the information communicated and documented in primary care consultations.

Analysis begun by cross-referencing transcripts with video-recordings to verify accuracy. Transcripts, video-recordings, and medical record entries were then reviewed repeatedly, individually and concomitantly, to become familiar with the content. Video-recordings were reviewed alongside transcripts to observe the nonverbal body language exhibited by patients and GPs. Six nonverbal aspects of communication were annotated in parentheses adjacent to the relevant text. These included posture, proximity, touch, body movements, facial expression, and eye behaviour.(35)

Short verbatim excerpts or 'meaning units' (encompassing nonverbal gestures) were juxtaposed with medical record entries using an adapted consultation proforma originally developed for examining the content of different primary care data sources.(36) Throughout analysis, excerpts from each consultation were kept together to allow contextualization of the data. 'Meaning units' were condensed by VH into an abbreviated narrative capturing the essence of patient's reason for presentation. Care was taken to preserve the patient voice by weaving verbatim descriptions from excerpts into a

For peer review only - http://bmjopen.bmj.com/site/about/guidelines.xhtml

reconfigured narrative. Patient reported bodily sensations were then compared with symptoms documented by GPs in the patients' medical record, and alignments and misalignments in documentation coded. Alignments were symptom information in medical records that both mirrored the content and verbal and non-verbal descriptors patients communicated; misalignments were symptoms that had either not been documented or differed from information given by patients. This task was independently undertaken by VH and two patient and public representatives (PPI) (MJ and JL); differences were resolved through further discussion and consensus with FW and JUS. Coded excerpts constituted the unit of analysis and encompassed multiple features of an individual symptom.

Coded alignments and misalignments were separated from original data sources and, with regular input from study authors, iteratively grouped into descriptive categories, informed by the symptom features listed in the 'gathering information' domains.(35)

Final categories were re-contextualised, and the features of patient's verbal descriptions and non-verbal gestures underpinning alignments and misalignments explored. Attention was given to isolated and discrepant excerpts to illuminate as much diversity of symptom expression encountered in general practice. We also explored differences in GP documentation by GP and patient demographic characteristics.

Ethical considerations

Research ethics approval was obtained from the East of Scotland Research Ethics Committee (REC) prior to commencing the study (reference: 19/ES/0057).

Patient and public involvement

Two patient and public involvement representatives (JL, ML) actively advised the research group from study inception to manuscript preparation. Both representatives were involved in the analysis and interpretation of the data, and will facilitate dissemination of study findings.

Results

Included consultations

Of the 84 consultations requested from the 'One in a Million' data repository, 64 were accompanied with a complete dataset, of which 28 met study eligibility criteria and were included in the final analysis (Figure 1). Participants comprised 28 patients, one caregiver, and 18 GPs. Overall, patients presented with 46 new or ongoing problems, captured in a total of 111 textual excerpts that were relevant for this analysis.

INSERT: Figure 1. Flow diagram of included consultations

For peer review only - http://bmjopen.bmj.com/site/about/guidelines.xhtml

Sample characteristics

Characteristics of included patients and GPs are presented in **Table 1** (the one caregiver's characteristics were unavailable). Patients were mostly female (*n*=16), aged 40 to 89 years, with varying educational attainment. GPs were aged 30 to 69 years and had been qualified as a GP for up to 31 years. Patients and GPs were all born in the UK and spoke English as their primary language. Apart from one patient of mixed ethnicity, our sample was white British. Patients presented with a variety of UGI and systemic bodily sensations (**Supplementary File A**).

For peer review only

Table 1. Sample characteristics

Patient characteristics	Number of patients (*N*=28)	GP characteristics	Number of GPs (*N*=18)
Sex		Sex	
Male	12	Male	10
Female	16	Female	8
Ethnicity*		Ethnicity	
White British/White other	26	White British/White other	18
Mixed/multiple ethnic groups	1	Mixed/multiple ethnic groups	-
Age		Age	
30-39	NA	30-39	5
40-49	5	40-49	5
50-59	6	50-59	7
60-69	6	60-69	1
70-79	9	70-79	-
80-89	2	80-89	-
Main language*		Main language	

For peer review only - http://bmjopen.bmj.com/site/about/guidelines.xhtml

English	27	English	18
**Country of birth**		**Country of birth**	
UK	28	UK	18
**Highest educational attainment**		**Years since qualified as a GP**	
Degree or higher degree	8	1-10	5
A/AS-levels or advanced diploma	2	11-20	6
Professional or vocational qualifications	6	21-30	6
Apprenticeship	3	31+	1
No qualifications	4		
**IMD quintiles**			
1st (least deprived)	6		
2nd	8		
3rd	4		
4th	5		
5th (most deprived)	5		

*Not reported (*n*=1); IMD, index of multiple deprivation; NA, not applicable (not eligible for inclusion)

GP alignments and misalignments

Overall, there was more evidence of GP misalignments (*n*=77) than alignments (*n*=44), with a median of 2 (*range:* 0–8) misalignments and 1 (*range:* 0–5) alignments, per consultation.

We identified ten categories reflecting ten clinical features which were informed by the 'gathering information' domains of the Calgary-Cambridge Guide (Figure 2 and Table 2).(35) Of these ten categories/features, seven related to the *'biomedical perspective'*, and three to the *'patient's perspective'* (Figure 2). Most misalignments occurred for abdominal sensations that were 'persistent or evolving', or when documenting the 'location', 'effect of on patients' life', and 'self-management' of abdominal sensations (Figure 3). Alignments were proportionally most common for 'aggravating and easing' factors, when abdominal sensations were described as 'pain', and when capturing patient's ideas and beliefs. Findings for all ten clinical features are detailed below. A summary of the main differences in patient descriptions of each feature between alignments and misalignments is presented in Table 2.

INSERT: Figure 2. Final categories representing the clinical features of abdominal sensations discussed in primary care consultations based on the 'gathering information' domains of the Calgary-Cambridge model

INSERT: Figure 3. Frequency of alignments and misalignments for each feature of patients' abdominal and systemic bodily sensations, according to the 'gathering information' domain of the Calgary-Cambridge Guide

Table 2. Differences in the characteristics of patients' verbal and non-verbal descriptions of abdominal sensations for alignments and misalignments

For peer review only - http://bmjopen.bmj.com/site/about/guidelines.xhtml

Clinical feature	Alignments	Misalignments
**Domain: Biomedical perspective**		
Persistent or evolving	Well-defined terms, articulating changes in quality, frequency, and intensity of worsening sensations	Non-definitive, figurative speech for bodily sensations that were worsening, improving but not resolved, or re-appraised as worsening
Location of pain/discomfort	Well-defined or medical terms, succinctly communicated; non-verbal gestures pointing to site of pain/discomfort	Difficulty articulating precise site of pain/discomfort; broad or global non-verbal gestures not indicating site of pain/discomfort
Aggravating and relieving factors	Communication of a relationship between activities of daily living and onset or worsening of sensations	Infrequent provocation of sensations; lengthy explanations regarding activities that provoked or worsened sensations; activities that relieved sensations
Discomfort	Adjectives or figurative speech for unpleasant sensations	Adjectives or figurative speech for unpleasant sensations
Pain	Pain explicitly articulated with qualifying adjectives, similes, and verbs describing an aversive sensation	Pain explicitly articulated with qualifying adjectives, similes, and verbs describing an aversive sensation
Duration	Communicating the day or number of weeks or months since onset of symptoms	Vague or oblique sentences without offering a timeframe
Self- management	Reporting sensations that were controlled or settling with medication	Reporting suboptimal therapeutic effect of medication
**Domain: Patient's perspective**		
Effects on life	*(no differences in patient descriptions between alignments and misalignments)*	
Ideas and beliefs	Plausible and convincing explanation as to underlying cause related to medication side-effects, flare-up of pre-existing condition, diet, or stress	Lack of a justified explanation for possible cause of sensations
Concerns about what symptoms might mean	Explicit concerns voiced about the possibility of cancer, or more broad concerns that something was not right	Verbalising concerns about the possibility symptoms had a sinister cause, but not explicitly mentioning cancer

Domain: Biomedical perspective

Persistent or evolving

Abdominal sensations that were persistent or evolving in frequency or intensity were described by patients most often, though were poorly reflected in medical records. Alignments occurred when patients used well-defined or concrete terms to indicate that their bodily sensations were progressing:

**Patient:** *"'my poo has got a lot freer and a lot looser probably in about the last six weeks. Most of the time when I go to the loo I will do some sort of poo as well at the same time. (shakes head)" (P22: F, 40-49 years)*	**Medical records:** last *6 weeks loose stools and more freq (GP19)*

For peer review only - http://bmjopen.bmj.com/site/about/guidelines.xhtml

Abdominal sensations described in non-definitive ("not getting better") or figurative speech, that were receding but not resolved, or that patients re-appraised as worsening, were characteristic of misalignments. In these instances, bodily sensations were erroneously documented as normal, resolving, or stabilised, respectively:

Patient: *"bowels are a bit sticky (intermittent eye contact, squeezing toothpaste motion with both hands) but I've been eating prunes and apricots and (laughs)*<br>*GP: "And do you tend to have problems in that area anyway?"*<br>***Patient:*** *"Not normally (looks upwards) and that was part of the symptoms last week" (P1: F, 60-69)*	Medical records:<br>*constipation improving with prunes. (GP23)*

Location of pain/discomfort

The location of patients' problem was discussed in relation to abdominal 'pain' or 'discomfort' on thirteen occasions. Misalignments were slightly more common than alignments. They occurred when pain/discomfort was communicated to be diffuse, move around, radiate to another location, or in instances when patients were unable to articulate the precise location and relied on large non-verbal hand gestures.

GP: *" So where are you point- (tilts head) is it the middle of the belly, upper belly?"*<br>Patient: *"I'm not really sure, (looking down at abdomen) but I'm just going like that with it, (rocking back and forth both arms cradling abdomen) because it…" (P18: F, 50-59 years)*	Medical records: *nothing recorded in relation to location (GP15)*

Location of abdominal pain/discomfort was accurately documented when a specific anatomical site was isolated, communicated in succinct and well-defined (sometimes medical) terms, and supplemented with non-verbal gestures that reinforced the specific site of their pain/discomfort:

Patient: *"I've had an abdominal discomfort for about four or five weeks. It's just sore, really, just below the sternum. (tips of fingers press into epigastric region). (P28: F, 50-59 years)*	Medical records: *epigastric pain (GP12)*

Aggravating and relieving factors

For peer review only - http://bmjopen.bmj.com/site/about/guidelines.xhtml

Aggravating and relieving factors referred to activities of daily living that provoked, aggravated, or worsened patients' bodily sensations. GPs' medical record entries accurately reflected information patients communicated in relation to this feature, when a causal relationship between an activity (i.e., stress, medication, and certain foods) and the onset or worsening of sensations was articulated:

Patient: *"It's like cucumber, red onion, tomatoes; it's certain things. I love to eat jacket potatoes, it just sets off heartburn. (P19: F, 40-49 years)*	Medical records: *Gastro-oesophageal reflux: with certain foods (GP18)*

Information about activities aggravating bodily sensations were omitted from medical records when they were described as infrequent or improving, gave lengthy explanations of the precipitating factors, or in one instance, communicated an activity that eased bodily sensations:

Patient: *"I have weeks now as long as I don't eat sugar and I don't have any stress it's often really quite good [...] At the moment it's a bit worse because I'm fighting for my son's funding and that gets me stressed. (shifts in chair, strokes forehead) Mostly it's so much better." (P25: F, 50-59 years)*	Medical records: *nothing recorded in relation to aggravating and relieving factors (GP20)*

Discomfort

Though there were no differences in patients' communication of discomfort between alignments and misalignments, patients used a range of adjectives and similes to describe abdominal sensations that were causing discomfort. Subtle verbal descriptors, such as "a nuisance" or as though "something was pressing down" conveyed discomfort as a sensation that was interfering or unpleasant. These descriptions were omitted or inaccurately reflected by GPs in most instances:

Patient: *"I can still feel a little something there (left hand touches hollow of neck) [...] a sensation up here but it can't be a physical blockage because they looked [...] it's just I feel like you've got an airlock..." (P20: F, 70-79 years)*	Medical records: *nothing recorded in relation to the quality of the sensation (GP18)*

Pain

GPs almost always documented 'pain' when it was mentioned. There were no observable differences in how patients described pain between alignments and the two misalignments. Patients also qualified 'pain' with supplementary adjectives, verbs, and similes, such as "terrible", "stabbing", and "like waves, like contractions", that communicated an aversive

For peer review only - http://bmjopen.bmj.com/site/about/guidelines.xhtml

For peer review only

sensation. Such qualifiers were characteristic of both alignments and misalignments and rarely captured in medical records.

Duration

The duration of patients' bodily sensations was infrequently described. GPs' accurately documented what patients communicated in relation to this feature in half of the eight instances it was discussed. Offering a discrete timeframe by giving the day of the week or number of weeks or months that had elapsed since the onset of bodily sensations, was equally characteristic of misalignments and alignments:

GP: *"So how long have you been poorly, now?"*<br>Patient: *"Well, it wasn't last week. What's today? Tuesday. It was the Friday week. Last Friday week, yes" (nods head) (P3: F, 70-79 years)*	Medical records: *lower abdo pelvic pain for 10 days (GP5)*

In contrast, vague or open-ended descriptions of the time since onset were slightly more typical of this feature being omitted from medical records.

Self-management

Patients described use of prescription and over-the-counter medication taken to manage their bodily sensations. GP misalignments, which were more commonplace than alignments, were mostly characterised by lengthy explanations that the medication had eased but not resolved, or had failed to appreciably control the severity of bodily sensations:

Patient: *"I took those capsules. I took one in the morning before anything else. And I took one at night before a meal [...] It [upper abdominal pain] settled a little bit. But it's still there (right hand presses upper abdomen) (P17: F, 70-79 years)*	Medical records: n*othing recorded in relation to self-management (GP14)*

In the few instances when GP documentation matched what patients had communicated, patients asserted that the medication was either helping to settle or control bodily sensations, or was having no effect:

Patient: *"I started taking prednisolone... [for colitis flare-up]. Well, it is working. (eye contact, nods head) I've been taking them for a couple of weeks and the condition is improved...(P21: M, 70-79 years)*	Medical records: *ulcerative colitis settled now with use of oral steroids (GP19)*

Domain: The patient's perspective

For peer review only - http://bmjopen.bmj.com/site/about/guidelines.xhtml

Effect on life

The effect of patients' bodily sensations on functional and social aspects of their life was discussed on twelve occasions but was rarely documented in medical records. There were no clear language differences between misalignments and alignments.

Ideas and beliefs

Patient's ideas and beliefs were documented on most occasions when patients offered a plausible explanation as to what may be causing their bodily sensations. Patients typically attributed sensations to medication side-effects, flare-up of a pre-existing condition, diet, and stress, which on occasion matched GPs ideas and beliefs. In the one instance that patient's ideas and beliefs were omitted from records, the patient was unable to justify the possible cause.

Concern about what symptoms might mean

Patients rarely expressed concerns about the cause or seriousness of their bodily sensations. In the two instances where patient's concerns were documented in medical records, patients had either relayed their partner's concern about "looking drawn" or explicitly asked the GP whether their bodily sensations could be due to an underlying cancer:

Patient: *"You don't think it's cancerous basically? (shakes head, right hand holding shirt collar)" (P25: F, 50-59 years)*	Medical records: *Very worried about cancer (GP20)*

Patients' concerns were typically not documented when the possibility of a sinister cause was voiced but cancer was not directly articulated:

Patient: *"There's nothing bad really down there? (touches upper abdomen with both hands) [...] Because it swells up quite a bit when it happened" (P17: F, 70-79 years)*	Medical records: *nothing recorded in relation to patient concerns (GP14)*

GP and patient characteristics

The frequency of alignments and misalignments for each clinical feature was insufficient for stratifying data to identify GP characteristics (sex, age, years since qualified as a GP) or patient characteristics (sex, age, educational attainment, social deprivation level) as additional sources of GP variation in how abdominal symptoms were documented.

For peer review only - http://bmjopen.bmj.com/site/about/guidelines.xhtml

Discussion

Summary of key findings

A wide range of clinical features pertaining to abdominal and systemic sensations were discussed between patients and GPs during primary care consultations. There was a gap between what patients communicated and the information about these bodily sensations GPs documented in medical records. Vague descriptors, figurative speech, and lengthy explanations of bodily sensations without an exact location, characterised features of patients' history that was not recorded, documented incompletely, or did not match what patients verbalised. Clinical features that were well-defined and succinct with precise gesticulations for abdominal sensations were well documented. Abdominal 'pain' was almost always documented compared to 'discomfort'. 'Discomfort' was conveyed using descriptors that indicated sensations that were unpleasant, whereas 'pain' was communicated as an aversive sensation. Abdominal features were omitted from medical records when large hand gesticulations, which supplemented or superseded verbal descriptions for the location of 'pain' or 'discomfort', were used.

Strengths and limitations

A strength of this work is that our findings are derived from direct comparison of naturalistic data, verified through video observation and medical records. To our knowledge, this is the only study to explore how patients describe undifferentiated symptoms of possible cancer during a consultation and compare this to what is subsequently documented in patients' medical record. Patients in this study communicated a range of abdominal symptoms that UGI cancer patients present with before diagnosis. This affords credible insight into the different ways patients may attempt to convey abdominal symptoms when presenting to primary care. By structuring our analysis around the 'gathering information' domains of an established communication teaching tool,(37) we were able to characterize nuances in patients' communication of abdominal sensations according to the clinical features doctors are trained to explore during the medical interview and as such are. directly applicable to medical training programs. As abdominal symptoms more often suggest conditions other than cancer, our findings are relevant to health care research concerned with enhancing the information gathered and integrated to safely differentiate serious from non-serious illness.

This study does have some limitations. Our findings reflect the immediately observable aspects of patient and GPs' verbal and non-verbal communication, rather than latent meaning. Thus, misalignments are not synonymous with a poor history as GPs may have

For peer review only - http://bmjopen.bmj.com/site/about/guidelines.xhtml

accurately interpreted patients intended meaning despite literal differences in written documentation.(38) What GPs documented may have been affected by individual characteristics of patients and GPs, patients' agenda, and the structure and relative brevity of medical records in UK general practice, which were beyond the scope of this study.(39) A complete patient history is more than the sum of its parts; parsing abdominal sensations from the patient narrative (as in our approach) potentially negates correct interpretation of the overall clinical picture. As we did not have access to records of patients' prior medical history or final diagnosis, we could not determine whether symptoms documented (or omitted) were truly indicative of serious disease. We therefore identified that there was a difference between what patients communicated and what GPs documented, not whether this difference was clinically important. Our sample was almost exclusively White British and native English speakers so our findings may not be transferable to practices serving patient populations with different characteristics. The focus of this study was limited to GPs. Therefore, our findings have most applicability to GPs in the UK and countries with similar health care structure. Our findings are likely to be relevant to other primary care providers in the UK though. For example, advanced nurse practitioners and physician associates who also see and manage patients with symptoms that could be caused by cancer. This study analyses the content of UK face-to-face consultations before Covid-19 prompted a shift to teleconsulting for triaging new patient problems in primary care.(40) Although face-to-face consultations are gradually resuming, remote consultations remain ubiquitous and may affect how patients communicate and what GPs document. Our findings are therefore unlikely to reflect communication or documentation practices occurring from remote patient-GP interactions where visual and non-verbal body language are occluded. Finally, data interpretation is subjective and influenced by the epistemic position of the research team; VH is a PhD student with a physiotherapy background where the character of patient's pain is important for ascertaining the involved structure. Consequently, certain patterns in the patient's history may have been more apparent than would have been if approached through a different lens. To ensure confirmability, data interpretation was regularly discussed and revised in collaboration with senior academic GPs (FW, JUS) and PPI representatives (MJ, JL).

Comparison with existing research

As previously mentioned, this is the only study to directly examine how patients describe abdominal sensations with what GPs subsequently document in the context of early cancer diagnosis.

Existing literature has shown that patients with cancer describe abdominal sensations using nuanced vocabulary that extends beyond well-defined medical nomenclature, which they

For peer review only - http://bmjopen.bmj.com/site/about/guidelines.xhtml

sometimes conflate. Humphrys *et al.* found esophago-gastric cancer patients used terms such as “stringy goo” to describe their phlegm and articulated that their legs were “getting thinner” to convey weight loss.(17) In the same study, patients held overlapping conceptualizations of heartburn, dyspepsia and indigestion which were inconsistent with the medical definition of each symptom.(17) For example, patients described heartburn, which is defined by the American Gastroenterological Association as a “burning sensation in the retrosternal area”, in terms of acid reflux or dyspepsia.(17,41) Bankhead *et al.* similarly noted interchangeable use of medical terms in interviews with women with suspected ovarian cancer, where increased abdominal size was referred to as ‘bloating’ instead of the appropriate medical term ‘abdominal distension’.(18) The most comparable study available is a recently published prospective cohort study of over 300 patients, which examined agreement of GP and patient-reported abdominal symptoms at referral for faecal immunochemical testing for suspected colorectal cancer.(19) Considerable variation in agreement was observed by symptom type and symptom severity: GPs most frequently reported abdominal pain and diarrhoea compared to reflux and constipation and increasingly reported abdominal pain and diarrhoea as patient-reported symptom severity on a six-point Likert scale increased (e.g., 63.6% and 53.9% respectively for minor discomfort *vs* 83.3% and 94.4% respectively for very severe discomfort). However, because studies have typically used interviews(17,18) and survey methods(19) for patients referred for suspected cancer or recently diagnosed with cancer, insights yielded may not fully capture how patients communicate bodily sensations to GPs that are yet to prompt suspicion. As we explored how patients communicate abdominal sensations during primary care consultations before diagnosis, our findings extend existing literature by identifying nuances in how patients may convey bodily sensations during earlier consultations. Further, our findings suggest that discrepancies in GPs reporting of abdominal sensations may continue into the medical records depending how they are communicated.

Future research

The finding that well-defined or painful abdominal sensations may be more salient to GPs than vague bodily sensations expressed as ‘discomfort’ warrants further evaluation. Differences between the information patients communicate about their abdominal sensations and what GPs document could be clinically important given the majority of patients with cancer are diagnosed following symptomatic presentation to primary care,(42) and post-hoc case reviews suggest some symptoms are interpreted incorrectly or not acted upon.(43,44) Establishing the importance of discrepancies in medical documentation requires an understanding of the meaning behind the words and phrases patients use, and the extent to which ‘lay’ descriptors can be reconciled with medical nomenclature. Exploring

For peer review only - http://bmjopen.bmj.com/site/about/guidelines.xhtml

this will be vital for developing strategies that facilitate medical documentation that more closely reflects the patient's history.

The influence of identified variations in patients' descriptions of abdominal sensations on GPs subsequent use of tests and referral pathways leading to an UGI cancer diagnosis will also be important to establish. In particular, the impact on the detection of harder-to-diagnose cancers via new rapid diagnostic centres (RDCs) for concerning non-specific symptoms not qualifying for NICE 'fast-track' referral requires consideration.(45) Earlier cancer diagnosis through RDCs is contingent upon prompt recognition of non-specific symptoms needing investigation, which may be hindered if potentially concerning vague symptoms or 'discomfort' are not recognized as important. Gender differences in the way patients describe symptoms of angina has been reported, with male patients using succinct language compared to female patients who used a wider repertoire of descriptors.(46) Thus, variations in descriptions of abdominal sensations by patient gender and other characteristics such as socioeconomic status should be explored given the potential implications for inequalities in the timely investigation of cancer. We identify a range of symptom features (e.g., aggravating and relieving factors and duration) capturing the nature and quality of patients' bodily sensations which are yet to be evaluated in studies developing risk prediction models for early cancer detection that limit inclusion to symptom type.(20,21,47,48) While most patients with cancer are diagnosed after a referral from their GP, other primary care practitioners are also involved in the timely recognition of symptoms of possible cancer warranting investigation.(49) As patients may feel more at ease communicating with such practitioners,(50) future similar studies that include a broader range of clinicians will also be important.

Implications for policy and practice

Abdominal sensations described in well-defined terms or as 'pain' may be deemed more significant by GPs than those communicated as 'discomfort' or described more vaguely, even though bodily sensations by are subjectively experienced states.(14) This could mean that patients more able to present their abdominal sensations succinctly or in terminology akin to medical terminology may be perceived to have more concerning symptoms which are investigated more promptly than patients with serious illness who do not articulate their symptoms as definitively. The integrity of current and future risk prediction models for UGI cancer (and other cancers of the abdomen)(20,21,51,52) which harvest coded and/or free-text symptoms from medical records may be undermined if consultation notes do not reflect the patient's history. Similarly, growing use of 'chatbot' or App technologies which utilize symptoms entered remotely by patients to suggest clinical management may be flawed if information about abdominal symptoms relies solely on medical terminology.(53)

For peer review only - http://bmjopen.bmj.com/site/about/guidelines.xhtml

GPs need to be cognizant that patients describe abdominal sensations in a variety of ways that differ from medical terminology which may not be indicative of their significance. Policy makers, and researchers and engineers in research and development should avoid interventions or guidance that reinforce false dichotomies that give precedence to abdominal symptoms packaged in medical language over 'lay' expressions or 'discomfort'. Instead, strategies that support GPs to interpret and act on patient meaning across a broader range of abdominal features rather than clinically exacting terminology is needed.

Funding

This work is supported by the CanTest Collaborative which is funded by Cancer Research UK (C8640/A23385), where VH is a PhD student, FMW is Director, and MJT, HS, and JE are Associate Directors. SA is supported by Cancer Research UK grants (C12292/A20861). The Bristol Archive Project was funded by the National Institute for Health Research (NIHR) School for Primary Care Research (208) and the South West GP Trust.

Contributions

VH and FW designed the study. RKB collected the primary data upon which this analysis was conducted. VH led the analysis and interpretation of the data with support from JUS, SA, and FW. VH drafted the manuscript. JUS, SA, FW, RKB, JL, MJ, MT, JE, HS, FMW critically reviewed and commented on the manuscript. All authors approved the final manuscript for submission.

Competing interests

None declared.

Data availability statement

No data are available.

References

1. Mulley AG, Trimble G, Elwyn Glyn. Stop the silent misdiagnosis: patients' preferences matter. *BMJ*. 2012;345:e6572. doi:10.1136/bmj.e6572
2. Committee on Diagnostic Error in Health Care; Institute of Medicine; The National Academies of Sciences, Engineering, and Medicine. Improving Diagnosis in Health Care. Balogh EP, Miller BT, Ball JR, editors. National Academies Press (US). 2015; Dec 29:1–472.
3. Gillard S, Benson J, Silverman J. Teaching and assessment of explanation and planning in medical schools in the United Kingdom: Cross sectional questionnaire survey. *Med Teach*. 2009;31(4):328–31.
4. Zwaan L, Thijs A, Wagner C, Timmermans DRM. Does inappropriate selectivity in

For peer review only - http://bmjopen.bmj.com/site/about/guidelines.xhtml

information use relate to diagnostic errors and patient harm? The diagnosis of patients with dyspnea. *Soc Sci Med.* 2013;91:32–8.
5. Singh H, Giardina TD, Meyer AND, Forjuoh SN, Reis MD, Thomas EJ. Types and origins of diagnostic errors in primary care settings. *JAMA Intern Med.* 2013;173(6):418–25.
6. Gruppen LD, Wolf FM, Billi JE. Information gathering and integration as sources of error in diagnostic decision making. *Med Decis Mak.* 1991;11(4):233–9.
7. Koo MM, von Wagner C, Abel GA, McPhail S, Hamilton W, Rubin GP, et al. The nature and frequency of abdominal symptoms in cancer patients and their associations with time to help-seeking: evidence from a national audit of cancer diagnosis. *J Public Health.* 2018 Sep 1;40(3):e388–95. doi: 10.1093/pubmed/fdx188.
8. Rasmussen S, Haastrup PF, Balasubramaniam K, Christensen R, Sondergaard Jens; Jarbol DE. Predictive values of upper gastrointestinal cancer alarm symptoms in the general population: a nationwide cohort study. *BMC Cancer.* 2018;18(440). doi:10.1186/s12885-018-4376-8.
9. National Institute for Health and Care Excellence. Gastrointestinal tract (upper) cancers - recognition and referral NG12. Available online: https://cks.nice.org.uk/topics/gastrointestinal-tract-upper-cancers-recognition-referral/ (accessed on 3 August 2021).
10. Calanzani N, Druce PE, Snudden C, Milley KM, Boscott R, Behiyat D, et al. Identifying Novel Biomarkers Ready for Evaluation in Low-Prevalence Populations for the Early Detection of Upper Gastrointestinal Cancers: A Systematic Review. *Adv Ther.* 2020; 38:793–834.
11. National Cancer Intelligence Network. Routes to Diagnosis 2006-2016 by Year. Available online: http://www.ncin.org.uk/publications/routes_to_diagnosis (accessed on 15 January 2021).
12. Fitzgerald RC, di Pietro M, O'Donovan M, Maroni R, Muldrew B, Debiram-Beecham I, et al. Cytosponge-trefoil factor 3 versus usual care to identify Barrett's oesophagus in a primary care setting: a multicentre, pragmatic, randomised controlled trial. *Lancet.* 2020;396(10247):333–44.
13. Malterud K, Guassora AD, Graungaard AH, Reventlow S. Understanding medical symptoms: a conceptual review and analysis. *Theor Med Bioeth.* 2015;36(6):411–24..
14. Rosendal M, Jarbøl DE, Pedersen AF, Andersen RS. Multiple perspectives on symptom interpretation in primary care research. *BMC Fam Pract.* 2013;14:167.
15. Sonnenberg A, Gogel HK. Translating vague complaints into precise symptoms : the implications of a poor medical history. *Eur J Gastroenterol Hepatol.* 2002;14(3):317–21.
16. Mills K, Birt L, Emery JD, Hall N, Banks J, Johnson M, et al. Understanding symptom appraisal and help-seeking in people with symptoms suggestive of pancreatic cancer: A qualitative study. *BMJ Open.* 2017;7(9):1–9.
17. Humphrys E, Walter FM, Rubin G, Emery JD, Johnson M, Richards A, et al. Patient symptom experience prior to a diagnosis of oesophageal or gastric cancer: A multi-

For peer review only - http://bmjopen.bmj.com/site/about/guidelines.xhtml

methods study. *BJGP Open*. 2020;4(1):1–15.
18. Bankhead CR, Collins C, Stokes-Lampard H, Rose P, Wilson S, Clements A, et al. Identifying symptoms of ovarian cancer: A qualitative and quantitative study. BJOG *An Int J Obstet Gynaecol*. 2008;115(8):1008–14.
19. Högberg C, Karling P, Rutegård J, Lilja M. Patient-reported and doctor-reported symptoms when faecal immunochemical tests are requested in primary care in the diagnosis of colorectal cancer and inflammatory bowel disease: A prospective study. *BMC Fam Pract*. 2020;21(1):1–12.
20. Stapley S, Peters TJ, Neal RD, Rose PW, Walter FM, Hamilton W. The risk of pancreatic cancer in symptomatic patients in primary care: A large case-control study using electronic records. *Br J Cancer*. 2012;106(12):1940–4.
21. Stapley S, Peters TJ, Neal RD, Rose PW, Walter FM, Hamilton W. The risk of oesophago-gastric cancer in symptomatic patients in primary care: A large case-control study using electronic records. *Br J Cancer*. 2013;108(1):25–31.
22. Shephard E, Neal R, Rose P, Walter F, Hamilton WT. Clinical features of kidney cancer in primary care: A case-control study using primary care records. *Br J Gen Pract*. 2013;63(609):250–5.
23. Shephard EA, Hamilton W, Neal RD, Rose PW, Walter FM. Symptoms of adult chronic and acute leukaemia before diagnosis: Large primary care case-control studies using electronic records. *Br J Gen Pract*. 2016;66(644):e182–8. doi: 10.3399/bjgp16X683989.
24. Tate AR, Martin AGR, Ali A, Cassell JA. Using free text information to explore how and when GPs code a diagnosis of ovarian cancer: An observational study using primary care records of patients with ovarian cancer. *BMJ Open*. 2011;1(1):1–7.
25. Koleck TA, Dreisbach C, Bourne PE, Bakken S. Natural language processing of symptoms documented in free-text narratives of electronic health records: A systematic review. *J Am Med Informatics Assoc*. 2019;26(4):364–79.
26. Edwards PJ, Bennett-Britton I, Ridd MJ, Booker M, Barnes RK. Factors affecting the documentation of spoken safety-netting advice in routine GP consultations: a cross-sectional study. *Br J Gen Pract*. 2021;71(712):e869-e876. doi: 10.3399/BJGP.2021.0195.2021.0195.
27. Weiner SJ, Wang S, Kelly B, Sharma G, Schwartz A. How accurate is the medical record? A comparison of the physician's note with a concealed audio recording in unannounced standardized patient encounters. *J Am Med Informatics Assoc*. 2020;27(5):770–5.
28. Barnes RK. One in a Million: A study of primary care consultations. Bristol: University of Bristol, 2017. Available online: https://doi.org/10.5523/bris.l3sq4s0w66ln1x20sye7s47wv (accessed on 3 August 2021).
29. Jepson M, Salisbury C, Ridd MJ, Metcalfe C, Garside L, Barnes RK. The "One in a Million" study: Creating a database of UK primary care consultations. *Br J Gen Pract*. 2017;67(658):e345–51.
30. Din NU, Ukoumunne OC, Rubin G, Hamilton W, Carter B, Stapley S, Neal RD. Age

For peer review only - http://bmjopen.bmj.com/site/about/guidelines.xhtml

and Gender Variations in Cancer Diagnostic Intervals in 15 Cancers: Analysis of Data from the UK Clinical Practice Research Datalink. *PLoS One*. 2015;10(5): e0127717. doi: 10.1371/journal.pone.0127717.
31. Erlingsson C, Brysiewicz P. A hands-on guide to doing content analysis. African J *Emerg Med*. 2017;7(3):93–9.
32. Hsieh HF, Shannon SE. Three approaches to qualitative content analysis. *Qual Health Res*. 2005;15(9):1277–88.
33. Bengtsson M. How to plan and perform a qualitative study using content analysis. *NursingPlus Open*. 2016;2:8–14.
34. Graneheim UH, Lindgren BM, Lundman B. Methodological challenges in qualitative content analysis: A discussion paper. *Nurse Educ Today*. 2017;56(May):29–34.
35. Silverman J, Kurtz S, Draper J. Skills for communicating with patients. 3rd edition. CRC Press Taylor & Francis Group. 2013. p69–130.
36. Procter S, Stewart K, Reeves D, Bowen L, Purdy S, Ridd M, et al. Complex consultations in primary care: A tool for assessing the range of health problems and issues addressed in general practice consultations. *BMC Fam Pract*. 2014;15(1):1–8.
37. Kurtz SM, Silverman JD. The Calgary-Cambridge Referenced Observation Guides: An aid to defining the curriculum and organizing the teaching in communication training programmes. *Med Educ*. 1996;30(2):83–9.
38. Amelung D, Whitaker KL, Lennard D, Ogden M, Sheringham J, Zhou Y, et al. Influence of doctor-patient conversations on behaviours of patients presenting to primary care with new or persistent symptoms: A video observation study. *BMJ Qual Saf*. 2020;29(3):198–208.
39. Colicchio TK, Cimino JJ. Clinicians' reasoning as reflected in electronic clinical note-entry and reading/retrieval: a systematic review and qualitative synthesis. *J Am Med Informatics Assoc*. 2019;26(2):172–84.
40. Mann C, Turner A, Salisbury C. The impact of remote consultations on personalised care Evidence briefing Commissioned by the Personalised Care Institute. *Natl Inst Heal Res*. 2021;(May):3–20.
41. Vakil N, Van Zanten S V., Kahrilas P, Dent J, Jones R, Bianchi LK, et al. The Montreal definition and classification of gastroesophageal reflux disease: A global evidence-based consensus. *Am J Gastroenterol*. 2006;101(8):1900-20.
42. Elliss-Brookes L, McPhail S, Ives A, Greenslade M, Shelton J, Hiom S, et al. Routes to diagnosis for cancer - Determining the patient journey using multiple routine data sets. *Br J Cancer*. 2012;107(8):1220–6.
43. Singh H, Davis Giardina T, Petersen LA, Smith M, Wilson L, Dismukes K, et al. Exploring Situational Awareness in Diagnostic Errors in Primary Care Conclusions—A framework of SA can help analyze and understand diagnostic errors in primary care settings that use EHRs. *BMJ Qual Saf*. 2012;21(110):30–8.
44. Lyratzopoulos G, Vedsted P, Singh H. Understanding missed opportunities for more timely diagnosis of cancer in symptomatic patients after presentation. *Br J Cancer*. 2015;112:S84–91. doi:10.1038/bjc.2015.47
45. Chapman D, Poirier V, Vulkan D, Fitzgerald K, Rubin G, Hamilton W, et al. First

For peer review only - http://bmjopen.bmj.com/site/about/guidelines.xhtml

results from five multidisciplinary diagnostic centre (MDC) projects for non-specific but concerning symptoms, possibly indicative of cancer. *Br J Cancer.* 2020;123:722–729.

46. Kreatsoulas C, Crea-Arsenio M, Shannon HS, Velianou JL, Giacomini M. Interpreting angina: symptoms along a gender continuum. *Open Heart.* 2016;3(1):e000376.
47. Lyratzopoulos G, Abel GA. Assessing patients at risk of symptomatic-but-as-yet-undiagnosed cancer in primary care using information from patient records. *Br J Cancer.* 2020;122(12):1729–31.
48. Koshiaris C, den Bruel A Van, Nicholson BD, Lay-Flurrie S, Richard Hobbs FD, Oke JL. Clinical prediction tools to identify patients at highest risk of myeloma in primary care: a retrospective open cohort study. *Br J Gen Pract.* 2021;71(706):E347–55.
49. Skrobanski H, Ream E, Poole K, Whitaker KL. Understanding primary care nurses' contribution to cancer early diagnosis: A systematic review. *Eur J Oncol Nurs.* 2019;41:149–64.
50. Cromme SK, Whitaker KL, Winstanley K, Renzi C, Smith CF, Wardle J. Worrying about wasting GP time as a barrier to help-seeking: A community-based, qualitative study. *Br J Gen Pract.* 2016;66(648):e474–82. doi:10.3399/bjgp16X685621.
51. Hippisley-Cox J, Coupland C. Identifying patients with suspected pancreatic cancer in primary care: Derivation and validation of an algorithm. *Br J Gen Pract.* 2012;62(594):38–45.
52. Hippisley-Cox, Julia and Coupland C. Identifying patients with suspected gastro-oesophageal cancer in primary care: derivation and validation of an algorithm. *Br J Gen Pract.* 2011;61(592):e707-14. doi: 10.3399/bjgp11X606609.
53. 98point6. On-demand, text-based primary care [Internet]. [cited 2021 Oct 6]. Available from: https://www.98point6.com/

For peer review only

For peer review only - http://bmjopen.bmj.com/site/about/guidelines.xhtml

Figure 1. Flow diagram of included consultations

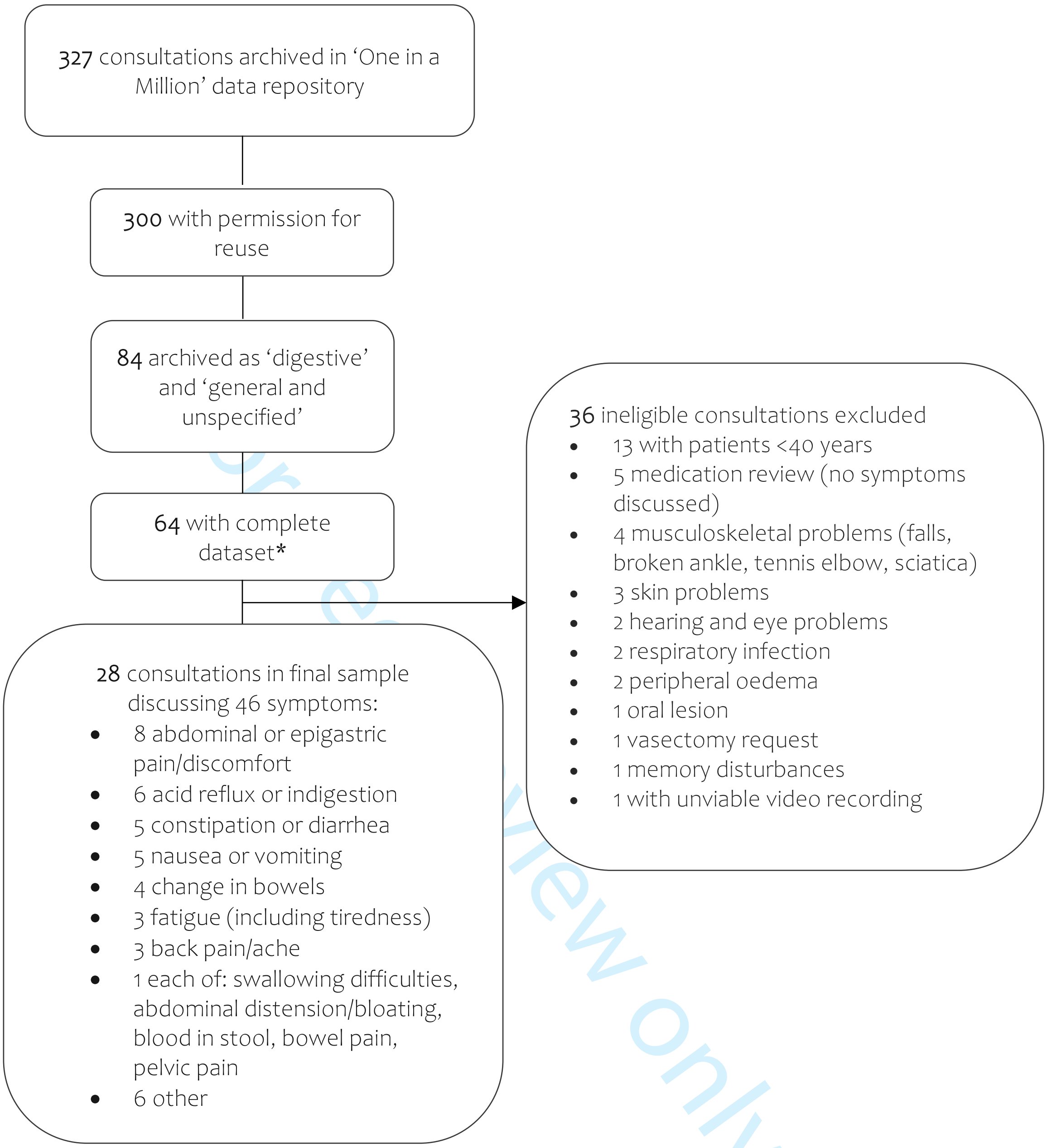

*Video recording, transcript, and medical record entry

Figure 2. Final categories representing the clinical features of abdominal sensations discussed in primary care consultations based on the 'gathering information' domains of the Calgary-Cambridge model

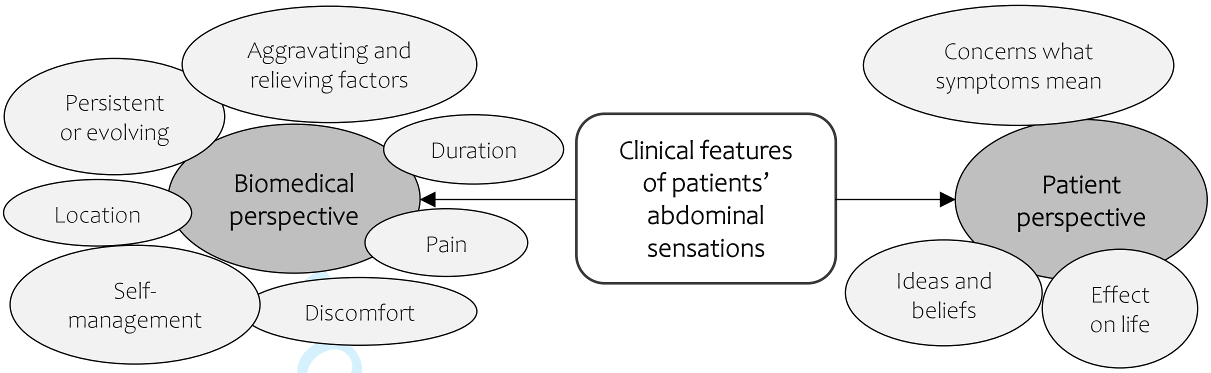

For peer review only

Figure 3. Frequency of alignments and misalignments for each feature of patients' abdominal and systemic bodily sensations, according to the 'gathering information' domain of the Calgary-Cambridge Guide

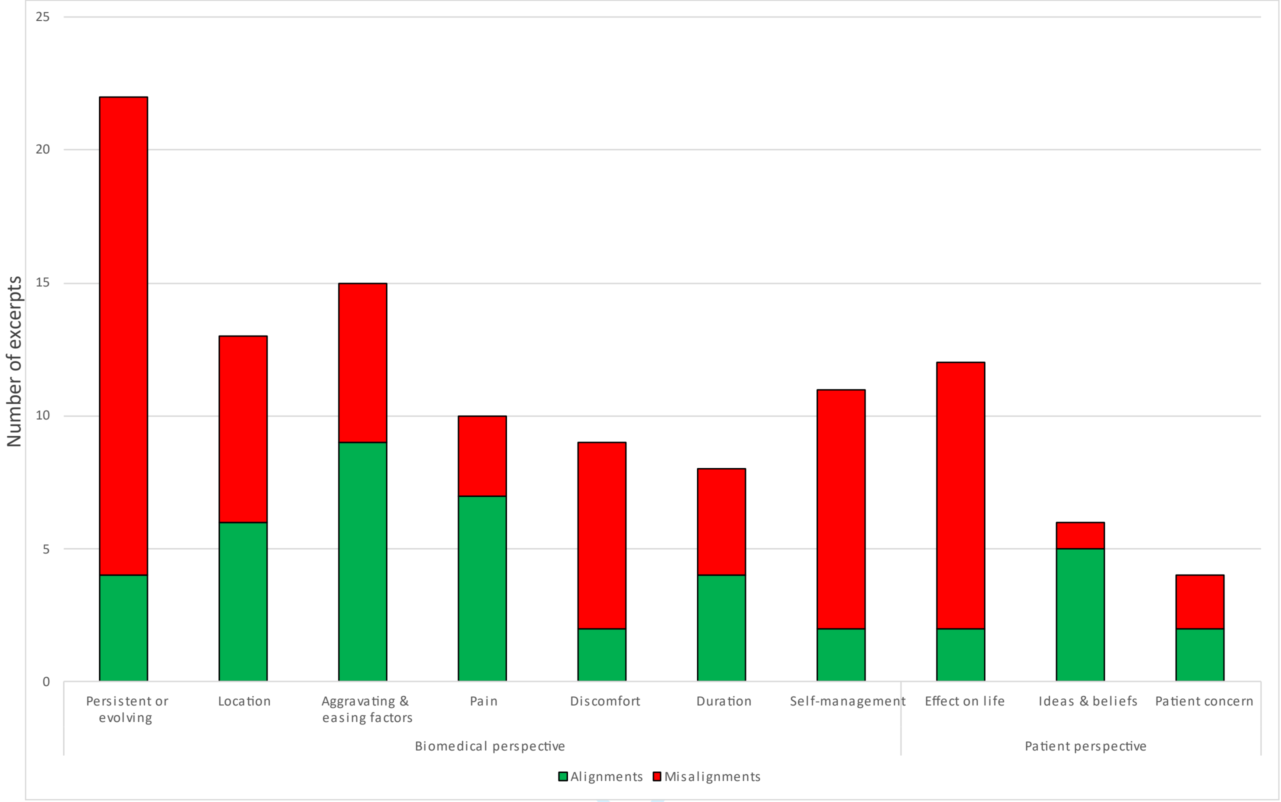

Supplementary file A. Details of included patients and their upper GI/systemic bodily sensations

Patient ID	Gender	Age	Bodily sensations
1	Female	66	Follow-up post-emergency operation for strangulated hernia, 'sticky bowels'
2	Female	71	Nausea, acid reflux
3	Female	72	Abdominal and pelvic pain, back ache
4	Male	55	Abdominal pain
5	Female	65	Nausea
6	Female	79	Heartburn, nausea
7	Female	73	Abdominal pain
8	Male	65	Abdominal and rectal pain, loose stools
9	Male	60	Acid reflux, indigestion
10	Male	59	Tiredness, headaches
11	Female	69	Abdominal distension/bloated
12	Male	73	Bowel pain, constipation
13	Male	83	Swallowing difficulties
14	Female	58	Back pain
15	Female	45	Fatigue, difficulty urinating
16	Male	81	Constipation
17	Female	77	Epigastric and back pain, nausea and vomiting
18	Female	53	Abdominal pain, nausea, diarrhoea, blood in stools
19	Female	42	Heartburn
20	Female	72	Reflux
21	Male	71	Loose stools, colitis flare-up
22	Female	41	Change in bowels, low haemoglobin
23	Female	70	Abdominal pain, change in bowels
24	Male	43	Fatigue, change in bowels
25	Female	59	Voice hoarseness, stomach problems
26	Male	74	Hernia
27	Male	64	Gastritis, follow-up of biopsy reversal
28	Female	53	Epigastric discomfort

For peer review only

COREQ (COnsolidated criteria for REporting Qualitative research) Checklist

A checklist of items that should be included in reports of qualitative research. You must report the page number in your manuscript where you consider each of the items listed in this checklist. If you have not included this information, either revise your manuscript accordingly before submitting or note N/A.

Topic	Item No.	Guide Questions/Description	Reported on Page No.
**Domain 1: Research team and reflexivity**			
Personal characteristics			
Interviewer/facilitator	1	Which author/s conducted the interview or focus group?	N/A
Credentials	2	What were the researcher's credentials? E.g. PhD, MD	15-16
Occupation	3	What was their occupation at the time of the study?	15-16
Gender	4	Was the researcher male or female?	N/A
Experience and training	5	What experience or training did the researcher have?	16
Relationship with participants			
Relationship established	6	Was a relationship established prior to study commencement?	N/A
Participant knowledge of the interviewer	7	What did the participants know about the researcher? e.g. personal goals, reasons for doing the research	N/A
Interviewer characteristics	8	What characteristics were reported about the inter viewer/facilitator? e.g. Bias, assumptions, reasons and interests in the research topic	N/A
**Domain 2: Study design**			
Theoretical framework			
Methodological orientation and Theory	9	What methodological orientation was stated to underpin the study? e.g. grounded theory, discourse analysis, ethnography, phenomenology, content analysis	2, 5
Participant selection			
Sampling	10	How were participants selected? e.g. purposive, convenience, consecutive, snowball	6
Method of approach	11	How were participants approached? e.g. face-to-face, telephone, mail, email	5, 6
Sample size	12	How many participants were in the study?	2, 7, 8
Non-participation	13	How many people refused to participate or dropped out? Reasons?	N/A
Setting			
Setting of data collection	14	Where was the data collected? e.g. home, clinic, workplace	5
Presence of non-participants	15	Was anyone else present besides the participants and researchers?	5
Description of sample	16	What are the important characteristics of the sample? e.g. demographic data, date	8
Data collection			
Interview guide	17	Were questions, prompts, guides provided by the authors? Was it pilot tested?	N/A
Repeat interviews	18	Were repeat inter views carried out? If yes, how many?	N/A
Audio/visual recording	19	Did the research use audio or visual recording to collect the data?	2, 5
Field notes	20	Were field notes made during and/or after the inter view or focus group?	6, 7
Duration	21	What was the duration of the inter views or focus group?	N/A
Data saturation	22	Was data saturation discussed?	N/A
Transcripts returned	23	Were transcripts returned to participants for comment and/or	N/A

For peer review only

Topic	Item No.	Guide Questions/Description	Reported on Page No.
		correction?	
**Domain 3: analysis and findings**			
Data analysis			
Number of data coders	24	How many data coders coded the data?	6-7
Description of the coding tree	25	Did authors provide a description of the coding tree?	6-7
Derivation of themes	26	Were themes identified in advance or derived from the data?	6-7
Software	27	What software, if applicable, was used to manage the data?	6-7
Participant checking	28	Did participants provide feedback on the findings?	15
Reporting			
Quotations presented	29	Were participant quotations presented to illustrate the themes/findings? Was each quotation identified? e.g. participant number	11-14
Data and findings consistent	30	Was there consistency between the data presented and the findings?	11-14
Clarity of major themes	31	Were major themes clearly presented in the findings?	11-14
Clarity of minor themes	32	Is there a description of diverse cases or discussion of minor themes?	11-14

Developed from: Tong A, Sainsbury P, Craig J. Consolidated criteria for reporting qualitative research (COREQ): a 32-item checklist for interviews and focus groups. *International Journal for Quality in Health Care*. 2007. Volume 19, Number 6: pp. 349 – 357

Once you have completed this checklist, please save a copy and upload it as part of your submission. DO NOT include this checklist as part of the main manuscript document. It must be uploaded as a separate file.

For peer review only

BMJ Open

Agreement between patient's description of abdominal symptoms of possible upper gastrointestinal cancer and General Practitioner consultation notes: a qualitative analysis of video-recorded UK primary care consultation data

Journal:	*BMJ Open*
Manuscript ID	bmjopen-2021-058766.R3
Article Type:	Original research
Date Submitted by the Author:	05-Dec-2022
Complete List of Authors:	Hardy, Victoria; University of Cambridge, Public Health and Primary Care<br>Usher-Smith, Juliet; University of Cambridge, Department of Public Health and Primary Care<br>Archer, Stephanie; University of Cambridge, Department of Public Health and Primary Care<br>Barnes, Rebecca ; University of Oxford Nuffield Department of Primary Care Health Sciences<br>Lancaster, John; University of Cambridge, Patient Representative c/o Public Health & Primary Care<br>Johnson, Margaret ; University of Cambridge, Patient Representative c/o Public Health & Primary Care<br>Thompson, Matthew; University of Washington, Department of Family Medicine<br>Emery, Jon; University of Melbourne Victorian Comprehensive Cancer Centre, Centre for Cancer Research and General Practice and Primary Care Academic Centre<br>Singh, Hardeep; Michael E DeBakey VA Medical Center, Houston Veterans Affairs Center for Innovations in Quality, Effectiveness and Safety; Baylor College of Medicine, Department of Medicine<br>Walter, Fiona; Queen Mary University of London, Wolfson Institute of Population Health, Barts and the London School of Medicine and Dentistry; University of Cambridge, Department of Public Health and Primary Care
**Primary Subject Heading**:	General practice / Family practice
Secondary Subject Heading:	Qualitative research, Evidence based practice, Medical education and training
Keywords:	PRIMARY CARE, QUALITATIVE RESEARCH, MEDICAL EDUCATION & TRAINING

SCHOLARONE™
Manuscripts

I, the Submitting Author has the right to grant and does grant on behalf of all authors of the Work (as defined in the below author licence), an exclusive licence and/or a non-exclusive licence for contributions from authors who are: i) UK Crown employees; ii) where BMJ has agreed a CC-BY licence shall apply, and/or iii) in accordance with the terms applicable for US Federal Government officers or employees acting as part of their official duties; on a worldwide, perpetual, irrevocable, royalty-free basis to BMJ Publishing Group Ltd ("BMJ") its licensees and where the relevant Journal is co-owned by BMJ to the co-owners of the Journal, to publish the Work in this journal and any other BMJ products and to exploit all rights, as set out in our licence.

The Submitting Author accepts and understands that any supply made under these terms is made by BMJ to the Submitting Author unless you are acting as an employee on behalf of your employer or a postgraduate student of an affiliated institution which is paying any applicable article publishing charge ("APC") for Open Access articles. Where the Submitting Author wishes to make the Work available on an Open Access basis (and intends to pay the relevant APC), the terms of reuse of such Open Access shall be governed by a Creative Commons licence – details of these licences and which Creative Commons licence will apply to this Work are set out in our licence referred to above.

Other than as permitted in any relevant BMJ Author's Self Archiving Policies, I confirm this Work has not been accepted for publication elsewhere, is not being considered for publication elsewhere and does not duplicate material already published. I confirm all authors consent to publication of this Work and authorise the granting of this licence.

For peer review only

Agreement between patient's description of abdominal symptoms of possible upper gastrointestinal cancer and General Practitioner consultation notes: a qualitative analysis of video-recorded UK primary care consultation data

Victoria Hardy[1], Juliet Usher-Smith[1], Stephanie Archer[1], Rebecca K. Barnes[2], John Lancaster[1], Margaret Johnson[1], Matthew Thompson[3], Jon Emery[4], Hardeep Singh[5], Fiona M. Walter[6,1]

Corresponding author
Victoria Hardy, Department of Public Health & Primary Care, University of Cambridge, Cambridge, UK
veh29@medschl.cam.ac.uk

Affiliations
[1]Department of Public Health & Primary Care, University of Cambridge, Cambridge, UK
[2]Nuffield Department of Primary Care Health Sciences, University of Oxford, Oxford, UK
[3]Department of Family Medicine, University of Washington, Seattle, Washington, USA
[4]Centre for Cancer Research and Department of General Practice, University of Melbourne, Victorian Comprehensive Cancer Centre, Melbourne, Victoria, Australia
[5]Center for Innovations in Quality, Effectiveness and Safety, Michael E. DeBakey VA Medical Center and Baylor College of Medicine, Houston, TX, USA.
[6]Wolfson Institute of Population Health, Barts and the London School of Medicine and Dentistry, Queen Mary University of London, UK

Key words: primary care, symptoms, diagnosis, qualitative research

Word count: 4493

For peer review only - http://bmjopen.bmj.com/site/about/guidelines.xhtml

Abstract

Introduction

Abdominal symptoms are common in primary care but infrequently might be due to an upper gastrointestinal (UGI) cancer. Patients' descriptions may differ from medical terminology used by General Practitioners (GPs). This may affect how information about abdominal symptoms possibly due to an UGI cancer are documented, creating potential missed opportunities for timely investigation.

Objectives

To explore how abdominal symptoms are communicated during primary care consultations, and identify characteristics of patients' descriptions that underpin variation in the accuracy and completeness with which they are documented in medical records.

Methods and analysis

Primary care consultation video recordings, transcripts and medical records from an existing dataset were screened for adults reporting abdominal symptoms. We conducted a qualitative content analysis to capture alignments (medical record entries matching patient verbal and non-verbal descriptions) and misalignments (symptom information omitted or differing from patient descriptions). Categories were informed by the Calgary-Cambridge Guide's 'gathering information' domains and patterns in descriptions explored.

Results

Our sample included 28 consultations (28 patients with 18 GPs): ten categories of different clinical features of abdominal symptoms were discussed. The information GPs documented about these features commonly did not match what patients described, with misalignments more common than alignments (67 vs 40 instances, respectively). Misalignments often featured patients using vague descriptors, figurative speech, lengthy explanations and broad hand gestures. Alignments were characterised by patients using well-defined terms, succinct descriptions and precise gestures for symptoms with an exact location. Abdominal sensations reported as 'pain' were almost always documented compared to expressions of 'discomfort'.

Conclusions

Abdominal symptoms that are well-defined or communicated as 'pain' may be more salient to GPs than those expressed vaguely or as 'discomfort'. Variable documentation of abdominal symptoms in medical records may have implications for the development of clinical decision support systems and decisions to investigate possible UGI cancer.

For peer review only - http://bmjopen.bmj.com/site/about/guidelines.xhtml

ARTICLE SUMMARY

Strengths and limitations

- This is the first study to explore similarities and differences between how patients describe and GPs document abdominal symptoms that could be due to an UGI cancer using naturalistic UK primary care consultation data
- We did not have access to patients' previous consultation history and so could not determine the clinical importance of symptom information that was either not documented in medical records, or did not reflect the patient's history.
- As our sample was mostly White British and native English speakers, patterns in how patients described and GPs documented abdominal symptoms may not be transferable to practices serving patient populations with different characteristics

For peer review only

For peer review only - http://bmjopen.bmj.com/site/about/guidelines.xhtml

Introduction

Sir William Osler, lauded as the father of modern medicine, implored his medical students to *"listen to your patient: he is telling you the diagnosis"*.(1) Over a century later, this adage remains germane. As depicted in the National Academy of Medicine's (NAM) conceptual model of the diagnostic process, an accurate and timely diagnosis is predicated on clinicians collecting sufficient data from the patient history.(2) Communication skills teaching is integral to medical training,(3) but suboptimal information gathering is common during the medical interview(4–6) and has been implicated in diagnostic error in primary care settings.(5)

Abdominal symptoms are common in patients presenting to primary care.(7) While such symptoms far more frequently herald benign conditions,(8) they are also described by patients diagnosed with upper gastrointestinal (UGI) cancers (i.e., gastric, oesophageal, and pancreatic), as well as other cancers within the abdomen.(7) In the United Kingdom (UK), compared with 'alarm' symptoms which have relatively high positive predictive value (PPV), abdominal symptoms alone do not meet the 3% PPV threshold recommended by the National Institute for Clinical Excellence (NICE) NG12 (2015) for a 'fast-track' suspected cancer referral.(9) Compounded by a lack of screening programmes or biomarkers reliably raising suspicion of UGI cancer in low prevalence populations,(10) patients with UGI cancers are disproportionately burdened with lengthy diagnostic intervals and late-stage diagnosis, associated with worse survival and patient experiences of healthcare.(11) While the novel 'CytoSponge' test (for Barrett's oesophagus, a precursor lesion for oesophageal cancer(12)) is a promising development for early detection, effective triage of abdominal symptoms for a timely UGI cancer diagnosis, remains currently largely contingent upon the patient history.

Symptoms are subjectively experienced changes in bodily sensations that reflect a perceived departure from the body's "normal" state.(13,14) Patients' experiences of bodily changes are the convergence of sociocultural(13) and experiential factors,(14) and may be communicated differently from standardised medical terminology used by general practitioners (GPs).(15) Studies of UGI cancer patients' symptom appraisal have shown they use a wide range of words and phrases to conceptualise abdominal changes(16,17) that are inconsistent with medical definitions familiar to GPs.(17,18) Without a common language, potentially significant bodily sensations risk being overlooked or misinterpreted, and the clinical picture distorted as 'lay' descriptions are translated into equivalent medical nomenclature.(15) Consequently, what GPs document in medical records may not be a complete or accurate representation of the patient's history,(19) potentially creating opportunities for diagnostic delay.

As well as being important at the patient-level for subsequent management decisions, the ways GPs (and other equivalent primary care providers in jurisdictions outside the UK) document patients' bodily sensations in medical records is important for population

For peer review only - http://bmjopen.bmj.com/site/about/guidelines.xhtml

approaches to early cancer detection. Initiatives in the UK currently harvest coded symptom data from primary care records to develop national guidelines and prediction tools that help GPs estimate patients' current or future cancer risk.(20–23) However, there is mounting interest in harnessing Natural Language Processing techniques to incorporate free-text symptoms.(24,25) Determining how patients describe abdominal sensations to GPs and the extent to which medical records reflect the patient's history is paramount for ensuring clinical decisions tools for UGI (and other) cancers facilitate effective use of tests and referral pathways.

To address this research need, we compared video-recorded primary care consultations featuring new or ongoing abdominal sensations that could be due to an UGI cancer with medical record documentation and explored similarities and differences between patient descriptions of bodily sensations and the way GPs document these as symptoms. We aimed to explore how abdominal symptoms are recorded in the medical record to identify the characteristics of patients' descriptions underpinning GP variation in the medical record entries. We also consider the implications of this on opportunities for earlier diagnosis of UGI cancers. Although other healthcare practitioners working at an advanced level of practice in primary care play an important role in the early recognition of symptoms of possible cancer, this study builds on existing observational research that has examined GP's appraisal (26) and documentation of patient's symptoms.(27)

Methods

Design

A cross-sectional descriptive study of video-recorded primary care consultations with linked medical record data entries from the *"One in a Million"* primary care consultation archive, analyzed using qualitative content analysis.

Data source

The *"One in a Million"* dataset was derived from the 'Bristol Archive project,' a prospective observational cohort study that sought to create a repository of routine primary care consultation data available for use in future research.(28) Between July 2014 and April 2015, twelve purposively sampled general practices in the West of England recorded unselected consultations with adult patients (aged 18 years and above) who presented to primary care over two to three half days.(29) A total of 327 consultations were recorded with linked patient, GP and practice demographic data, and available medical record entries. 89.8% (n=300) of patients consented to selected datasets being re-used in future research, subject to specific ethical approval. All these recordings were anonymized for spoken identifiers, transcribed verbatim and coded for their content according to sixteen "problem type(s)" based on the International Classification of Primary Care – 2nd Edition (ICPC-2) classification scheme.(29) Consultations were assigned more than one code if multiple problems were discussed. We requested all available data for 84 consultations archived as 'digestive' or

For peer review only - http://bmjopen.bmj.com/site/about/guidelines.xhtml

'general and unspecified'(29) to yield data directly relevant for addressing our research question.

Eligibility criteria and assessment

We included consultations if there was: a) an accompanying videorecording, transcript, and medical record entry, and b) communication of any new or ongoing abdominal and systemic sensations that could reflect an underlying UGI cancer. We limited inclusion to patients aged 40 years and above, because UGI cancers below this age are rare.(30) Consultations of patients known to have a previous or current cancer diagnosis based on information disclosed during the video-recording were excluded, as a history of cancer provokes more suspicion of serious illness among GPs and patients, and we were interested in GPs' appraisal of patient's bodily sensations in an as-yet undiagnosed population. We also excluded consultations during which no relevant bodily sensations were communicated. This was determined by abstracting all bodily sensations discussed in consultations into a pre-prepared Microsoft Excel form and assessing each as 'include', 'exclude', or 'unsure'. Eligibility assessment was initially undertaken by VH (a non-clinical PhD student) before final adjudication of relevant bodily sensations by FW (a senior academic GP). Consultations for which there were no relevant bodily sensations were excluded at this juncture.

Sampling

As this study was an analysis of a pre-existing archived dataset and no further data collection was possible, we included all consultations of symptomatic patients that met study eligibility criteria.

Data analysis

Data were analysed using a manifest qualitative content analysis, following the four phases outlined by Erlingsson *et al.*: 'familiarising oneself with the data', 'dividing and condensing the text into meaning units', 'formulating codes', and 'developing categories and themes'.(31) A manifest qualitative content analysis was selected as it is the most suitable analytical technique for exploring and describing visible and explicit patterns (as opposed to the underlying meaning of text as in latent content analysis) across data sources that incorporate visual and textual communication data.(32–34) Data analysis was conducted in excel by VH, with analysis facilitated by SA (senior non-clinical researcher), and data interpretation verified by FW, JUS (senior academic GPs), and PPI (JL, MJ).

We employed a mixed deductive and inductive approach whereby the analytical framework was informed by the 'gathering information' domains of the Calgary-Cambridge Guide. We selected the Calgary-Cambridge Guide over other possible instruments (e.g., the Roter Interaction Analysis System) as it is an evidence-based teaching and assessment resource that is used in medical training programmes internationally to develop doctors' verbal and non-verbal communication skills and structure consultations. The guide delineates the consultation into distinct phases, including 'initiating the session', 'gathering information',

For peer review only - http://bmjopen.bmj.com/site/about/guidelines.xhtml

'physical examination', 'explanation and planning', and 'closing the session'. The 'gathering information' component guides doctors through the collection of symptom information relating to the biomedical perspective, patient perspective, and background information that should be elicited during the patient history. Thus, the Calgary-Cambridge Guide offers an ecologically valid framework for guiding the deconstruction and analysis of the information communicated and documented in primary care consultations.

Analysis begun by cross-referencing transcripts with video-recordings to verify accuracy. Transcripts, video-recordings, and medical record entries were then reviewed repeatedly, individually and concomitantly, to become familiar with the content. Video-recordings were reviewed alongside transcripts to observe the nonverbal body language exhibited by patients and GPs. Six nonverbal aspects of communication were annotated in parentheses adjacent to the relevant text. These included posture, proximity, touch, body movements, facial expression, and eye behaviour.(35)

Short verbatim excerpts or 'meaning units' (encompassing nonverbal gestures) were juxtaposed with medical record entries using an adapted consultation proforma originally developed for examining the content of different primary care data sources.(36) Throughout analysis, excerpts from each consultation were kept together to allow contextualization of the data. 'Meaning units' were condensed by VH into an abbreviated narrative capturing the essence of patient's reason for presentation. Care was taken to preserve the patient voice by weaving verbatim descriptions from excerpts into a reconfigured narrative. Patient reported bodily sensations were then compared with symptoms documented by GPs in the patients' medical record, and alignments and misalignments in documentation coded. Alignments were symptom information in medical records that both mirrored the content and verbal and non-verbal descriptors patients communicated; misalignments were symptoms that had either not been documented or differed from information given by patients. This task was independently undertaken by VH and two patient and public representatives (PPI) (MJ and JL); differences were resolved through further discussion and consensus with FW and JUS. Coded excerpts constituted the unit of analysis and encompassed multiple features of an individual symptom.

Coded alignments and misalignments were separated from original data sources and, with regular input from study authors, iteratively grouped into descriptive categories, informed by the symptom features listed in the 'gathering information' domains.(35)

Final categories were re-contextualised, and the features of patient's verbal descriptions and non-verbal gestures underpinning alignments and misalignments explored. Attention was given to isolated and discrepant excerpts to illuminate as much diversity of symptom expression encountered in general practice. We also explored differences in GP documentation by GP and patient demographic characteristics.

Patient and public involvement

For peer review only - http://bmjopen.bmj.com/site/about/guidelines.xhtml

Two patient and public involvement representatives (JL, ML) actively advised the research group from study inception to manuscript preparation. Both representatives were involved in the analysis and interpretation of the data, and will facilitate dissemination of study findings.

Results

Included consultations

Of the 77 consultations requested from the ‘One in a Million’ data repository, 64 were accompanied with a complete dataset, of which 28 met study eligibility criteria and were included in the final analysis (**Figure 1**). Participants comprised 28 patients, one caregiver, and 18 GPs. Overall, patients presented with 46 new or ongoing problems, captured in a total of 111 textual excerpts that were relevant for this analysis.

INSERT: Figure 1. Flow diagram of included consultations

Sample characteristics

Characteristics of included patients and GPs are presented in **Table 1** (the one caregiver’s characteristics were unavailable)**.** Patients were mostly female (*n*=16), aged 40 to 89 years, with varying educational attainment. GPs were aged 30 to 69 years and had been qualified as a GP for up to 31 years. Patients and GPs were all born in the UK and spoke English as their primary language. Apart from one patient of mixed ethnicity, our sample was white British. Patients presented with a variety of UGI and systemic bodily sensations (**Supplementary File A)**.

For peer review only - http://bmjopen.bmj.com/site/about/guidelines.xhtml

Table 1. Sample characteristics

Patient characteristics	Number of patients (*N*=28)	GP characteristics	Number of GPs (*N*=18)
**Sex**		**Sex**	
Male	12	Male	10
Female	16	Female	8
**Ethnicity***		**Ethnicity**	
White British/White other	26	White British/White other	18
Mixed/multiple ethnic groups	1	Mixed/multiple ethnic groups	-
**Age**		**Age**	
30-39	NA	30-39	5
40-49	5	40-49	5
50-59	6	50-59	7
60-69	6	60-69	1
70-79	9	70-79	-
80-89	2	80-89	-
**Main language***		**Main language**	
English	27	English	18
**Country of birth**		**Country of birth**	
UK	28	UK	18
**Highest educational attainment**		**Years since qualified as a GP**	
Degree or higher degree	8	1-10	5
A/AS-levels or advanced diploma	2	11-20	6
Professional or vocational qualifications	6	21-30	6
Apprenticeship	3	31+	1
No qualifications	4		
**IMD quintiles**			
1st (least deprived)	6		
2nd	8		
3rd	4		
4th	5		
5th (most deprived)	5		

*Not reported (*n*=1); IMD, index of multiple deprivation; NA, not applicable (not eligible for inclusion)

GP alignments and misalignments

Overall, there was more evidence of GP misalignments (*n*=67) than alignments (*n*=40), with a median of 2 (*range:* 0–8) misalignments and 1 (*range:* 0–5) alignments, per consultation.

We identified ten categories reflecting ten clinical features which were informed by the 'gathering information' domains of the Calgary-Cambridge Guide (**Figure 2** and **Table 2**).(35) Of these ten categories/features, seven related to the *'biomedical perspective',* and three to the *'patient's perspective' (***Figure 2***)*. Most misalignments occurred for abdominal sensations that were 'persistent or evolving', or when documenting the 'location', 'effect of on patients' life', and 'self-management' of abdominal sensations (**Figure 3**). Alignments were

For peer review only - http://bmjopen.bmj.com/site/about/guidelines.xhtml

proportionally most common for 'aggravating and easing' factors, when abdominal sensations were described as 'pain', and when capturing patient's ideas and beliefs. Findings for all ten clinical features are detailed below. A summary of the main differences in patient descriptions of each feature between alignments and misalignments is presented in **Table 2**.

INSERT: Figure 2. Final categories representing the clinical features of abdominal sensations discussed in primary care consultations based on the 'gathering information' domains of the Calgary-Cambridge model

INSERT: Figure 3. Frequency of alignments and misalignments for each feature of patients' abdominal and systemic bodily sensations, according to the 'gathering information' domain of the Calgary-Cambridge Guide

For peer review only

Table 2. Differences in the characteristics of patients' verbal and non-verbal descriptions of abdominal sensations for alignments and misalignments

Clinical feature	Alignments	Misalignments
**Domain: Biomedical perspective**		
Persistent or evolving	Well-defined terms, articulating changes in quality, frequency, and intensity of worsening sensations	Non-definitive, figurative speech for bodily sensations that were worsening, improving but not resolved, or re-appraised as worsening
Location of pain/discomfort	Well-defined or medical terms, succinctly communicated; non-verbal gestures pointing to site of pain/discomfort	Difficulty articulating precise site of pain/discomfort; broad or global non-verbal gestures not indicating site of pain/discomfort
Aggravating and relieving factors	Communication of a relationship between activities of daily living and onset or worsening of sensations	Infrequent provocation of sensations; lengthy explanations regarding activities that provoked or worsened sensations; activities that relieved sensations
Discomfort	Adjectives or figurative speech for unpleasant sensations	Adjectives or figurative speech for unpleasant sensations
Pain	Pain explicitly articulated with qualifying adjectives, similes, and verbs describing an aversive sensation	Pain explicitly articulated with qualifying adjectives, similes, and verbs describing an aversive sensation
Duration	Communicating the day or number of weeks or months since onset of symptoms	Vague or oblique sentences without offering a timeframe
Self- management	Reporting sensations that were controlled or settling with medication	Reporting suboptimal therapeutic effect of medication
**Domain: Patient's perspective**		
Effects on life	*(no differences in patient descriptions between alignments and misalignments)*	
Ideas and beliefs	Plausible and convincing explanation as to underlying cause related to medication side-effects, flare-up of pre-existing condition, diet, or stress	Lack of a justified explanation for possible cause of sensations
Concerns about what symptoms might mean	Explicit concerns voiced about the possibility of cancer, or more broad concerns that something was not right	Verbalising concerns about the possibility symptoms had a sinister cause, but not explicitly mentioning cancer

Domain: Biomedical perspective

For peer review only - http://bmjopen.bmj.com/site/about/guidelines.xhtml

Persistent or evolving

Abdominal sensations that were persistent or evolving in frequency or intensity were described by patients most often (on 22 occasions), though were poorly reflected in medical records. Alignments occurred when patients used well-defined or concrete terms to indicate that their bodily sensations were progressing:

**Patient:** *"'my poo has got a lot freer and a lot looser probably in about the last six weeks. Most of the time when I go to the loo I will do some sort of poo as well at the same time. (shakes head)" (P22: F, 40-49 years)*	**Medical records:** last *6 weeks loose stools and more freq (GP19)*

Abdominal sensations described in non-definitive ("not getting better") or figurative speech, that were receding but not resolved, or that patients re-appraised as worsening, were characteristic of misalignments. In these instances, bodily sensations were erroneously documented as normal, resolving, or stabilised, respectively:

**Patient:** *"bowels are a bit sticky (intermittent eye contact, squeezing toothpaste motion with both hands) but I've been eating prunes and apricots and (laughs)*<br><br>***GP:*** *"And do you tend to have problems in that area anyway?"*<br><br>***Patient:*** *"Not normally (looks upwards) and that was part of the symptoms last week" (P1: F, 60-69)*	**Medical records:** *constipation improving with prunes. (GP23)*

Location of pain/discomfort

The location of patients' problem was discussed in relation to abdominal 'pain' or 'discomfort' on thirteen occasions. Misalignments were slightly more common than alignments. They occurred when pain/discomfort was communicated to be diffuse, move around, radiate to another location, or in instances when patients were unable to articulate the precise location and relied on large non-verbal hand gestures.

**GP:** *"So where are you point- (tilts head) is it the middle of the belly, upper belly?"*<br><br>**Patient:** *"I'm not really sure, (looking down at abdomen) but I'm just going like that with it, (rocking back and forth both arms cradling abdomen) because it…" (P18: F, 50-59 years)*	**Medical records:** *nothing recorded in relation to location (GP15)*

Location of abdominal pain/discomfort was accurately documented when a specific anatomical site was isolated, communicated in succinct and well-defined (sometimes medical) terms, and supplemented with non-verbal gestures that reinforced the specific site of their pain/discomfort:

For peer review only

For peer review only - http://bmjopen.bmj.com/site/about/guidelines.xhtml

Patient: *"I've had an abdominal discomfort for about four or five weeks. It's just sore, really, just below the sternum. (tips of fingers press into epigastric region). (P28: F, 50-59 years)*

Medical records: *epigastric pain (GP12)*

Aggravating and relieving factors

Aggravating and relieving factors referred to activities of daily living that provoked, aggravated, or worsened patients' bodily sensations. GPs' medical record entries accurately reflected information patients communicated in relation to this feature, when a causal relationship between an activity (i.e., stress, medication, and certain foods) and the onset or worsening of sensations was articulated:

Patient: *"It's like cucumber, red onion, tomatoes; it's certain things. I love to eat jacket potatoes, it just sets off heartburn. (P19: F, 40-49 years)*

Medical records: *Gastro-oesophageal reflux: with certain foods (GP18)*

Information about activities aggravating bodily sensations were omitted from medical records when they were described as infrequent or improving, gave lengthy explanations of the precipitating factors, or in one instance, communicated an activity that eased bodily sensations:

Patient: *"I have weeks now as long as I don't eat sugar and I don't have any stress it's often really quite good […] At the moment it's a bit worse because I'm fighting for my son's funding and that gets me stressed. (shifts in chair, strokes forehead) Mostly it's so much better." (P25: F, 50-59 years)*

Medical records: *nothing recorded in relation to aggravating and relieving factors (GP20)*

Discomfort

Though there were no differences in patients' communication of discomfort between alignments and misalignments, patients used a range of adjectives and similes to describe abdominal sensations that were causing discomfort. Subtle verbal descriptors, such as "a nuisance" or as though "something was pressing down" conveyed discomfort as a sensation that was interfering and unpleasant, and distinct from 'pain'. Patient's descriptions of discomfort were omitted or inaccurately reflected by GPs in most instances:

Patient: *"I can still feel a little something there (left hand touches hollow of neck) […] a sensation up here but it can't be a physical blockage because they looked […] it's just I feel like you've got an airlock..." (P20: F, 70-79 years)*

Medical records: *nothing recorded in relation to the quality of the sensation (GP18)*

Pain

For peer review only - http://bmjopen.bmj.com/site/about/guidelines.xhtml

GPs almost always documented 'pain' when it was mentioned. There were no observable differences in how patients described pain between alignments and misalignments. However, patients typically qualified 'pain' with supplementary adjectives, verbs, and similes, including "terrible", "stabbing", and "like waves, like contractions" that communicated an aversive sensation. Such qualifiers were characteristic of both alignments and misalignments and rarely captured in medical records.

Duration

The duration of patients' bodily sensations was infrequently described. GPs' accurately documented what patients communicated in relation to this feature in half of the eight instances it was discussed. Offering a discrete timeframe by giving the day of the week or number of weeks or months that had elapsed since the onset of bodily sensations, was equally characteristic of misalignments and alignments:

**GP:** *"So how long have you been poorly, now?"*<br>**Patient:** *"Well, it wasn't last week. What's today? Tuesday. It was the Friday week. Last Friday week, yes" (nods head) (P3: F, 70-79 years)*	**Medical records:** *lower abdo pelvic pain for 10 days (GP5)*

In contrast, vague or open-ended descriptions of the time since onset were slightly more typical of this feature being omitted from medical records.

Self-management

Patients described use of prescription and over-the-counter medication taken to manage their bodily sensations. GP misalignments, which were more commonplace than alignments, were mostly characterised by lengthy explanations that the medication had eased but not resolved, or had failed to appreciably control the severity of bodily sensations:

**Patient:** *"I took those capsules. I took one in the morning before anything else. And I took one at night before a meal […] It [upper abdominal pain] settled a little bit. But it's still there (right hand presses upper abdomen) (P17: F, 70-79 years)*	**Medical records:** nothing *recorded in relation to self-management (GP14)*

In the two instances GP's documentation matched what patients had communicated, patients asserted that the medication was either helping to settle or control bodily sensations, or was having no effect:

**Patient:** *"I started taking prednisolone… [for colitis flare-up]. Well, it is working. (eye contact, nods head) I've been taking them for a couple of weeks and the condition is improved…(P21: M, 70-79 years)*	**Medical records:** *ulcerative colitis settled now with use of oral steroids (GP19)*

Domain: The patient's perspective

For peer review only - http://bmjopen.bmj.com/site/about/guidelines.xhtml

Effect on life

The effect of patients' bodily sensations on functional and social aspects of their life was discussed on twelve occasions but was rarely documented in medical records. There were no clear language differences between misalignments and alignments.

Ideas and beliefs

Patient's ideas and beliefs were documented on most occasions when patients offered a plausible explanation as to what may be causing their bodily sensations. Patients typically attributed sensations to medication side-effects, flare-up of a pre-existing condition, diet, and stress, which on occasion matched GPs ideas and beliefs. In the one instance that patient's ideas and beliefs were omitted from records, the patient was unable to justify the possible cause.

Concern about what symptoms might mean

Patients rarely expressed concerns about the cause or seriousness of their bodily sensations. In the two instances where patient's concerns were documented in medical records, patients had either relayed their partner's concern about "looking drawn" or explicitly asked the GP whether their bodily sensations could be due to an underlying cancer:

**Patient:** *"You don't think it's cancerous basically? (shakes head, right hand holding shirt collar)" (P25: F, 50-59 years)*	**Medical records:** *Very worried about cancer (GP20)*

Patients' concerns were typically not documented when the possibility of a sinister cause was voiced but cancer was not directly articulated:

**Patient:** *"There's nothing bad really down there? (touches upper abdomen with both hands) […] Because it swells up quite a bit when it happened" (P17: F, 70-79 years)*	**Medical records:** *nothing recorded in relation to patient concerns (GP14)*

GP and patient characteristics

The frequency of alignments and misalignments for each clinical feature was insufficient for stratifying data to identify GP characteristics (sex, age, years since qualified as a GP) or patient characteristics (sex, age, educational attainment, social deprivation level) as additional sources of GP variation in how abdominal symptoms were documented.

For peer review only - http://bmjopen.bmj.com/site/about/guidelines.xhtml

For peer review only

Discussion

Summary of key findings

A wide range of clinical features pertaining to abdominal and systemic sensations were discussed between patients and GPs during primary care consultations. There was a gap between what patients communicated and the information about these bodily sensations GPs documented in medical records. Vague descriptors, figurative speech, and lengthy explanations of bodily sensations without an exact location, characterised features of patients' history that was not recorded, documented incompletely, or did not match what patients verbalised. Clinical features that were well-defined and succinct with precise gesticulations for abdominal sensations were well documented. Abdominal 'pain' was almost always documented compared to 'discomfort'. 'Discomfort' was conveyed using descriptors that indicated sensations that were unpleasant, whereas 'pain' was communicated as an aversive sensation. Abdominal features were omitted from medical records when large hand gesticulations, which supplemented or superseded verbal descriptions for the location of 'pain' or 'discomfort', were used.

Strengths and limitations

A strength of this work is that our findings are derived from direct comparison of naturalistic data, verified through video observation and medical records. To our knowledge, this is the only study to explore how patients describe undifferentiated symptoms of possible cancer during a consultation and compare this to what is subsequently documented in patients' medical record. Patients in this study communicated a range of abdominal symptoms that UGI cancer patients present with before diagnosis. This affords credible insight into the different ways patients may attempt to convey abdominal symptoms when presenting to primary care. By structuring our analysis around the 'gathering information' domains of an established communication teaching tool,(37) we were able to characterize nuances in patients' communication of abdominal sensations according to the clinical features doctors are trained to explore during the medical interview and as such are. directly applicable to medical training programs. As abdominal symptoms more often suggest conditions other than cancer, our findings are relevant to health care research concerned with enhancing the information gathered and integrated to safely differentiate serious from non-serious illness.

This study does have some limitations. Our findings reflect the immediately observable aspects of patient and GPs' verbal and non-verbal communication, rather than latent meaning. Thus, misalignments are not synonymous with a poor history as GPs may have accurately interpreted patients intended meaning despite literal differences in written documentation.(38) What GPs documented may have been affected by individual characteristics of patients and GPs, patients' agenda, and the structure and relative brevity of medical records in UK general practice, which were beyond the scope of this study.(39) A complete patient history is more than the sum of its parts; parsing abdominal sensations from the patient narrative (as in our approach) potentially negates correct interpretation of the

For peer review only - http://bmjopen.bmj.com/site/about/guidelines.xhtml

overall clinical picture. As we did not have access to records of patients' prior medical history or final diagnosis, we could not determine whether symptoms documented (or omitted) were truly indicative of serious disease. We therefore identified that there was a difference between what patients communicated and what GPs documented, not whether this difference was clinically important. Our sample was almost exclusively White British and native English speakers so our findings may not be transferable to practices serving patient populations with different characteristics. The focus of this study was limited to GPs. Therefore, our findings have most applicability to GPs in the UK and countries with similar health care structure. Our findings are likely to be relevant to other primary care providers in the UK though. For example, advanced nurse practitioners and physician associates who also see and manage patients with symptoms that could be caused by cancer. This study analyses the content of UK face-to-face consultations before Covid-19 prompted a shift to teleconsulting for triaging new patient problems in primary care.(40) Although face-to-face consultations are gradually resuming, remote consultations remain ubiquitous and may affect how patients communicate and what GPs document. Our findings are therefore unlikely to reflect communication or documentation practices occurring from remote patient-GP interactions where visual and non-verbal body language are occluded. Finally, data interpretation is subjective and influenced by the epistemic position of the research team; VH is a PhD student with a physiotherapy background where the character of patient's pain is important for ascertaining the involved structure. Consequently, certain patterns in the patient's history may have been more apparent than would have been if approached through a different lens. To ensure confirmability, data interpretation was regularly discussed and revised in collaboration with senior academic GPs (FW, JUS) and PPI representatives (MJ, JL).

Comparison with existing research

As previously mentioned, this is the only study to directly examine how patients describe abdominal sensations with what GPs subsequently document in the context of early cancer diagnosis.

Existing literature has shown that patients with cancer describe abdominal sensations using nuanced vocabulary that extends beyond well-defined medical nomenclature, which they sometimes conflate. Humphrys *et al*. found esophago-gastric cancer patients used terms such as "stringy goo" to describe their phlegm and articulated that their legs were "getting thinner" to convey weight loss.(17) In the same study, patients held overlapping conceptualizations of heartburn, dyspepsia and indigestion which were inconsistent with the medical definition of each symptom.(17) For example, patients described heartburn, which is defined by the American Gastroenterological Association as a "burning sensation in the retrosternal area", in terms of acid reflux or dyspepsia.(17,41) Bankhead *et al*. similarly noted interchangeable use of medical terms in interviews with women with suspected ovarian cancer, where increased abdominal size was referred to as 'bloating' instead of the appropriate medical term 'abdominal distension'.(18) The most comparable study available is a recently published

For peer review only - http://bmjopen.bmj.com/site/about/guidelines.xhtml

prospective cohort study of over 300 patients, which examined agreement of GP and patient-reported abdominal symptoms at referral for faecal immunochemical testing for suspected colorectal cancer.(19) Considerable variation in agreement was observed by symptom type and symptom severity: GPs most frequently reported abdominal pain and diarrhoea compared to reflux and constipation and increasingly reported abdominal pain and diarrhoea as patient-reported symptom severity on a six-point Likert scale increased (e.g., 63.6% and 53.9% respectively for minor discomfort *vs* 83.3% and 94.4% respectively for very severe discomfort). However, because studies have typically used interviews(17,18) and survey methods(19) for patients referred for suspected cancer or recently diagnosed with cancer, insights yielded may not fully capture how patients communicate bodily sensations to GPs that are yet to prompt suspicion. As we explored how patients communicate abdominal sensations during primary care consultations before diagnosis, our findings extend existing literature by identifying nuances in how patients may convey bodily sensations during earlier consultations. Further, our findings suggest that discrepancies in GPs reporting of abdominal sensations may continue into the medical records depending how they are communicated.

Future research

The finding that well-defined or painful abdominal sensations may be more salient to GPs than vague bodily sensations expressed as 'discomfort' warrants further evaluation. Differences between the information patients communicate about their abdominal sensations and what GPs document could be clinically important given the majority of patients with cancer are diagnosed following symptomatic presentation to primary care,(42) and post-hoc case reviews suggest some symptoms are interpreted incorrectly or not acted upon.(43,44) Establishing the importance of discrepancies in medical documentation requires an understanding of the meaning behind the words and phrases patients use, and the extent to which 'lay' descriptors can be reconciled with medical nomenclature. Exploring this will be vital for developing strategies that facilitate medical documentation that more closely reflects the patient's history.

The influence of identified variations in patients' descriptions of abdominal sensations on GPs subsequent use of tests and referral pathways leading to an UGI cancer diagnosis will also be important to establish. In particular, the impact on the detection of harder-to-diagnose cancers via new rapid diagnostic centres (RDCs) for concerning non-specific symptoms not qualifying for NICE 'fast-track' referral requires consideration.(45) Earlier cancer diagnosis through RDCs is contingent upon prompt recognition of non-specific symptoms needing investigation, which may be hindered if potentially concerning vague symptoms or 'discomfort' are not recognized as important. Gender differences in the way patients describe symptoms of angina has been reported, with male patients using succinct language compared to female patients who used a wider repertoire of descriptors.(46) Thus, variations in descriptions of abdominal sensations by patient gender and other characteristics such as socioeconomic status should be explored given the potential implications for inequalities in

For peer review only - http://bmjopen.bmj.com/site/about/guidelines.xhtml

the timely investigation of cancer. We identify a range of symptom features (e.g., aggravating and relieving factors and duration) capturing the nature and quality of patients' bodily sensations which are yet to be evaluated in studies developing risk prediction models for early cancer detection that limit inclusion to symptom type.(20,21,47,48) While most patients with cancer are diagnosed after a referral from their GP, other primary care practitioners are also involved in the timely recognition of symptoms of possible cancer warranting investigation.(49) As patients may feel more at ease communicating with such practitioners,(50) future similar studies that include a broader range of clinicians will also be important.

Implications for policy and practice

Abdominal sensations described in well-defined terms or as 'pain' may be deemed more significant by GPs than those communicated as 'discomfort' or described more vaguely, even though bodily sensations by are subjectively experienced states.(14) This could mean that patients more able to present their abdominal sensations succinctly or in terminology akin to medical terminology may be perceived to have more concerning symptoms which are investigated more promptly than patients with serious illness who do not articulate their symptoms as definitively. The integrity of current and future risk prediction models for UGI cancer (and other cancers of the abdomen)(20,21,51,52) which harvest coded and/or free-text symptoms from medical records may be undermined if consultation notes do not reflect the patient's history. Similarly, growing use of 'chatbot' or App technologies which utilize symptoms entered remotely by patients to suggest clinical management may be flawed if information about abdominal symptoms relies solely on medical terminology.(53)

GPs need to be cognizant that patients describe abdominal sensations in a variety of ways that differ from medical terminology which may not be indicative of their significance. Policy makers, and researchers and engineers in research and development should avoid interventions or guidance that reinforce false dichotomies that give precedence to abdominal symptoms packaged in medical language over 'lay' expressions or 'discomfort'. Instead, strategies that support GPs to interpret and act on patient meaning across a broader range of abdominal features rather than clinically exacting terminology is needed.

Funding

This work is supported by the CanTest Collaborative which is funded by Cancer Research UK (C8640/A23385), where VH is a PhD student, FW is Director, and MJT, HS, and JE are Associate Directors. SA is supported by Cancer Research UK grants (C12292/A20861). The Bristol Archive Project was funded by the National Institute for Health Research (NIHR) School for Primary Care Research (208) and the South West GP Trust.

Contributions

For peer review only - http://bmjopen.bmj.com/site/about/guidelines.xhtml

VH and FW designed the study. RKB collected the primary data upon which this analysis was conducted. VH led the analysis and interpretation of the data with support from JUS, SA, and FW. VH drafted the manuscript. JUS, SA, FW, RKB, JL, MJ, MT, JE, HS, FW critically reviewed and commented on the manuscript. All authors approved the final manuscript for submission.

Competing interests

None declared.

Ethics approval

Research ethics approval was obtained from the East of Scotland Research Ethics Committee (REC) prior to commencing the study (reference: 19/ES/0057).

Data availability statement

No data are available.

References

1. Mulley AG, Trimble G, Elwyn Glyn. Stop the silent misdiagnosis: patients' preferences matter. *BMJ.* **2012**;345:e6572. doi:10.1136/bmj.e6572
2. Committee on Diagnostic Error in Health Care; Institute of Medicine; The National Academies of Sciences, Engineering, and Medicine. Improving Diagnosis in Health Care. Balogh EP, Miller BT, Ball JR, editors. National Academies Press (US). **2015**; Dec 29:1–472.
3. Gillard S, Benson J, Silverman J. Teaching and assessment of explanation and planning in medical schools in the United Kingdom: Cross sectional questionnaire survey. *Med Teach.* **2009**;31(4):328–31.
4. Zwaan L, Thijs A, Wagner C, Timmermans DRM. Does inappropriate selectivity in information use relate to diagnostic errors and patient harm? The diagnosis of patients with dyspnea. *Soc Sci Med.* **2013**;91:32–8.
5. Singh H, Giardina TD, Meyer AND, Forjuoh SN, Reis MD, Thomas EJ. Types and origins of diagnostic errors in primary care settings. *JAMA Intern Med*. **2013**;173(6):418–25.
6. Gruppen LD, Wolf FM, Billi JE. Information gathering and integration as sources of error in diagnostic decision making. *Med Decis Mak.* **1991**;11(4):233–9.
7. Koo MM, von Wagner C, Abel GA, McPhail S, Hamilton W, Rubin GP, et al. The nature and frequency of abdominal symptoms in cancer patients and their associations with time to help-seeking: evidence from a national audit of cancer diagnosis. *J Public Health.* **2018** Sep 1;40(3):e388–95. doi: 10.1093/pubmed/fdx188.
8. Rasmussen S, Haastrup PF, Balasubramaniam K, Christensen R, Sondergaard Jens; Jarbol DE. Predictive values of upper gastrointestinal cancer alarm symptoms in the general population: a nationwide cohort study. *BMC Cancer.* **2018**;18(440). doi:10.1186/s12885-018-4376-8.
9. National Institute for Health and Care Excellence. Gastrointestinal tract (upper)

For peer review only - http://bmjopen.bmj.com/site/about/guidelines.xhtml

cancers - recognition and referral NG12. Available online: https://cks.nice.org.uk/topics/gastrointestinal-tract-upper-cancers-recognition-referral/ (accessed on 3 August 2021).
10. Calanzani N, Druce PE, Snudden C, Milley KM, Boscott R, Behiyat D, et al. Identifying Novel Biomarkers Ready for Evaluation in Low-Prevalence Populations for the Early Detection of Upper Gastrointestinal Cancers: A Systematic Review. *Adv Ther*. **2020**; 38:793–834.
11. National Cancer Intelligence Network. Routes to Diagnosis 2006-2016 by Year. Available online: http://www.ncin.org.uk/publications/routes_to_diagnosis (accessed on 15 January 2021).
12. Fitzgerald RC, di Pietro M, O'Donovan M, Maroni R, Muldrew B, Debiram-Beecham I, et al. Cytosponge-trefoil factor 3 versus usual care to identify Barrett's oesophagus in a primary care setting: a multicentre, pragmatic, randomised controlled trial. *Lancet.* **2020**;396(10247):333–44.
13. Malterud K, Guassora AD, Graungaard AH, Reventlow S. Understanding medical symptoms: a conceptual review and analysis. *Theor Med Bioeth.* **2015**;36(6):411–24..
14. Rosendal M, Jarbøl DE, Pedersen AF, Andersen RS. Multiple perspectives on symptom interpretation in primary care research. *BMC Fam Pract*. **2013**;14:167.
15. Sonnenberg A, Gogel HK. Translating vague complaints into precise symptoms : the implications of a poor medical history. *Eur J Gastroenterol Hepatol.* **2002**;14(3):317–21.
16. Mills K, Birt L, Emery JD, Hall N, Banks J, Johnson M, et al. Understanding symptom appraisal and help-seeking in people with symptoms suggestive of pancreatic cancer: A qualitative study. *BMJ Open.* **2017**;7(9):1–9.
17. Humphrys E, Walter FM, Rubin G, Emery JD, Johnson M, Richards A, et al. Patient symptom experience prior to a diagnosis of oesophageal or gastric cancer: A multi-methods study. *BJGP Open*. **2020**;4(1):1–15.
18. Bankhead CR, Collins C, Stokes-Lampard H, Rose P, Wilson S, Clements A, et al. Identifying symptoms of ovarian cancer: A qualitative and quantitative study. BJOG *An Int J Obstet Gynaecol.* **2008**;115(8):1008–14.
19. Högberg C, Karling P, Rutegård J, Lilja M. Patient-reported and doctor-reported symptoms when faecal immunochemical tests are requested in primary care in the diagnosis of colorectal cancer and inflammatory bowel disease: A prospective study. *BMC Fam Pract.* **2020**;21(1):1–12.
20. Stapley S, Peters TJ, Neal RD, Rose PW, Walter FM, Hamilton W. The risk of pancreatic cancer in symptomatic patients in primary care: A large case-control study using electronic records. *Br J Cancer.* **2012**;106(12):1940–4.
21. Stapley S, Peters TJ, Neal RD, Rose PW, Walter FM, Hamilton W. The risk of oesophago-gastric cancer in symptomatic patients in primary care: A large case-control study using electronic records. *Br J Cancer.* **2013**;108(1):25–31.
22. Shephard E, Neal R, Rose P, Walter F, Hamilton WT. Clinical features of kidney cancer in primary care: A case-control study using primary care records. *Br J Gen Pract.* **2013**;63(609):250–5.
23. Shephard EA, Hamilton W, Neal RD, Rose PW, Walter FM. Symptoms of adult chronic and acute leukaemia before diagnosis: Large primary care case-control studies using electronic records. *Br J Gen Pract.* **2016**;66(644):e182–8. doi: 10.3399/bjgp16X683989.

For peer review only - http://bmjopen.bmj.com/site/about/guidelines.xhtml

24. Tate AR, Martin AGR, Ali A, Cassell JA. Using free text information to explore how and when GPs code a diagnosis of ovarian cancer: An observational study using primary care records of patients with ovarian cancer. *BMJ Open*. **2011**;1(1):1–7.
25. Koleck TA, Dreisbach C, Bourne PE, Bakken S. Natural language processing of symptoms documented in free-text narratives of electronic health records: A systematic review. *J Am Med Informatics Assoc.* **2019**;26(4):364–79.
26. Edwards PJ, Bennett-Britton I, Ridd MJ, Booker M, Barnes RK. Factors affecting the documentation of spoken safety-netting advice in routine GP consultations: a cross-sectional study. *Br J Gen Pract.* **2021**;71(712):e869-e876. doi: 10.3399/BJGP.2021.0195.2021.0195.
27. Weiner SJ, Wang S, Kelly B, Sharma G, Schwartz A. How accurate is the medical record? A comparison of the physician's note with a concealed audio recording in unannounced standardized patient encounters. *J Am Med Informatics Assoc*. **2020**;27(5):770–5.
28. Barnes RK. One in a Million: A study of primary care consultations. Bristol: University of Bristol, 2017. Available online: https://doi.org/10.5523/bris.l3sq4s0w66ln1x20sye7s47wv (accessed on 3 August 2021).
29. Jepson M, Salisbury C, Ridd MJ, Metcalfe C, Garside L, Barnes RK. The "One in a Million" study: Creating a database of UK primary care consultations. *Br J Gen Pract.* **2017**;67(658):e345–51.
30. Din NU, Ukoumunne OC, Rubin G, Hamilton W, Carter B, Stapley S, Neal RD. Age and Gender Variations in Cancer Diagnostic Intervals in 15 Cancers: Analysis of Data from the UK Clinical Practice Research Datalink. *PLoS One.* **2015**;10(5): e0127717. doi: 10.1371/journal.pone.0127717.
31. Erlingsson C, Brysiewicz P. A hands-on guide to doing content analysis. African J *Emerg Med.* **2017**;7(3):93–9.
32. Hsieh HF, Shannon SE. Three approaches to qualitative content analysis. *Qual Health Res.* **2005**;15(9):1277–88.
33. Bengtsson M. How to plan and perform a qualitative study using content analysis. *NursingPlus Open*. **2016**;2:8–14.
34. Graneheim UH, Lindgren BM, Lundman B. Methodological challenges in qualitative content analysis: A discussion paper. *Nurse Educ Today.* **2017**;56(May):29–34.
35. Silverman J, Kurtz S, Draper J. Skills for communicating with patients. 3rd edition. CRC Press Taylor & Francis Group. 2013. p69–130.
36. Procter S, Stewart K, Reeves D, Bowen L, Purdy S, Ridd M, et al. Complex consultations in primary care: A tool for assessing the range of health problems and issues addressed in general practice consultations. *BMC Fam Pract.* **2014**;15(1):1–8.
37. Kurtz SM, Silverman JD. The Calgary-Cambridge Referenced Observation Guides: An aid to defining the curriculum and organizing the teaching in communication training programmes. *Med Educ.* **1996**;30(2):83–9.
38. Amelung D, Whitaker KL, Lennard D, Ogden M, Sheringham J, Zhou Y, et al. Influence of doctor-patient conversations on behaviours of patients presenting to primary care with new or persistent symptoms: A video observation study. *BMJ Qual Saf.* **2020**;29(3):198–208.
39. Colicchio TK, Cimino JJ. Clinicians' reasoning as reflected in electronic clinical note-entry and reading/retrieval: a systematic review and qualitative synthesis. *J Am Med*

For peer review only - http://bmjopen.bmj.com/site/about/guidelines.xhtml

Informatics Assoc. **2019**;26(2):172–84.
40. Mann C, Turner A, Salisbury C. The impact of remote consultations on personalised care Evidence briefing Commissioned by the Personalised Care Institute. *Natl Inst Heal Res.* **2021**;(May):3–20.
41. Vakil N, Van Zanten S V., Kahrilas P, Dent J, Jones R, Bianchi LK, et al. The Montreal definition and classification of gastroesophageal reflux disease: A global evidence-based consensus. *Am J Gastroenterol.* **2006**;101(8):1900-20.
42. Elliss-Brookes L, McPhail S, Ives A, Greenslade M, Shelton J, Hiom S, et al. Routes to diagnosis for cancer - Determining the patient journey using multiple routine data sets. *Br J Cancer.* **2012**;107(8):1220–6.
43. Singh H, Davis Giardina T, Petersen LA, Smith M, Wilson L, Dismukes K, et al. Exploring Situational Awareness in Diagnostic Errors in Primary Care Conclusions—A framework of SA can help analyze and understand diagnostic errors in primary care settings that use EHRs. *BMJ Qual Saf.* **2012**;21(110):30–8.
44. Lyratzopoulos G, Vedsted P, Singh H. Understanding missed opportunities for more timely diagnosis of cancer in symptomatic patients after presentation. *Br J Cancer.* **2015**;112:S84–91. doi:10.1038/bjc.2015.47
45. Chapman D, Poirier V, Vulkan D, Fitzgerald K, Rubin G, Hamilton W, et al. First results from five multidisciplinary diagnostic centre (MDC) projects for non-specific but concerning symptoms, possibly indicative of cancer. *Br J Cancer.* **2020**;123:722–729.
46. Kreatsoulas C, Crea-Arsenio M, Shannon HS, Velianou JL, Giacomini M. Interpreting angina: symptoms along a gender continuum. *Open Heart.* **2016**;3(1):e000376.
47. Lyratzopoulos G, Abel GA. Assessing patients at risk of symptomatic-but-as-yet-undiagnosed cancer in primary care using information from patient records. *Br J Cancer.* **2020**;122(12):1729–31.
48. Koshiaris C, den Bruel A Van, Nicholson BD, Lay-Flurrie S, Richard Hobbs FD, Oke JL. Clinical prediction tools to identify patients at highest risk of myeloma in primary care: a retrospective open cohort study. *Br J Gen Pract*. **2021**;71(706):E347–55.
49. Skrobanski H, Ream E, Poole K, Whitaker KL. Understanding primary care nurses' contribution to cancer early diagnosis: A systematic review. *Eur J Oncol Nurs.* **2019**;41:149–64.
50. Cromme SK, Whitaker KL, Winstanley K, Renzi C, Smith CF, Wardle J. Worrying about wasting GP time as a barrier to help-seeking: A community-based, qualitative study. *Br J Gen Pract.* **2016**;66(648):e474–82. doi:10.3399/bjgp16X685621.
51. Hippisley-Cox J, Coupland C. Identifying patients with suspected pancreatic cancer in primary care: Derivation and validation of an algorithm. *Br J Gen Pract.* **2012**;62(594):38–45.
52. Hippisley-Cox, Julia and Coupland C. Identifying patients with suspected gastro-oesophageal cancer in primary care: derivation and validation of an algorithm. *Br J Gen Pract.* **2011**;61(592):e707-14. doi: 10.3399/bjgp11X606609.
53. 98point6. On-demand, text-based primary care [Internet]. [cited 2021 Oct 6]. Available from: https://www.98point6.com/

For peer review only - http://bmjopen.bmj.com/site/about/guidelines.xhtml

Figure 1. Flow diagram of included consultations

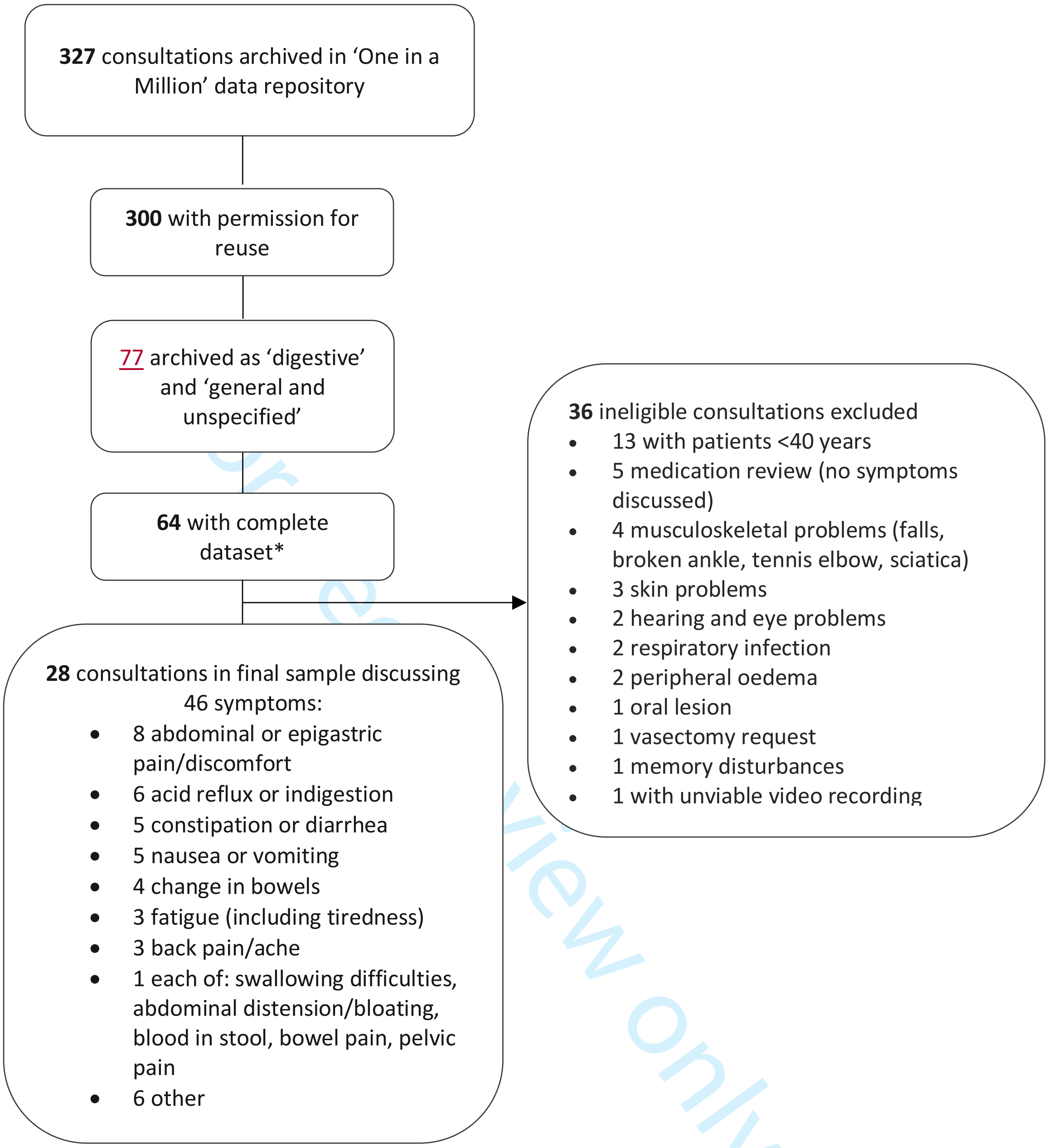

*Video recording, transcript, and medical record entry

Figure 2. Final categories representing the clinical features of abdominal sensations discussed in primary care consultations based on the 'gathering information' domains of the Calgary-Cambridge model

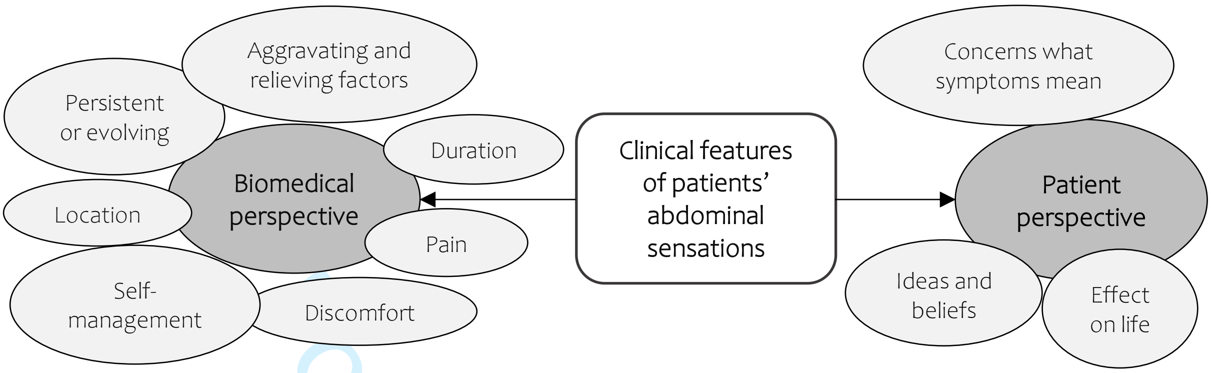

For peer review only

Figure 3. Frequency of alignments and misalignments for each feature of patients' abdominal and systemic bodily sensations, according to the 'gathering information' domain of the Calgary-Cambridge Guide

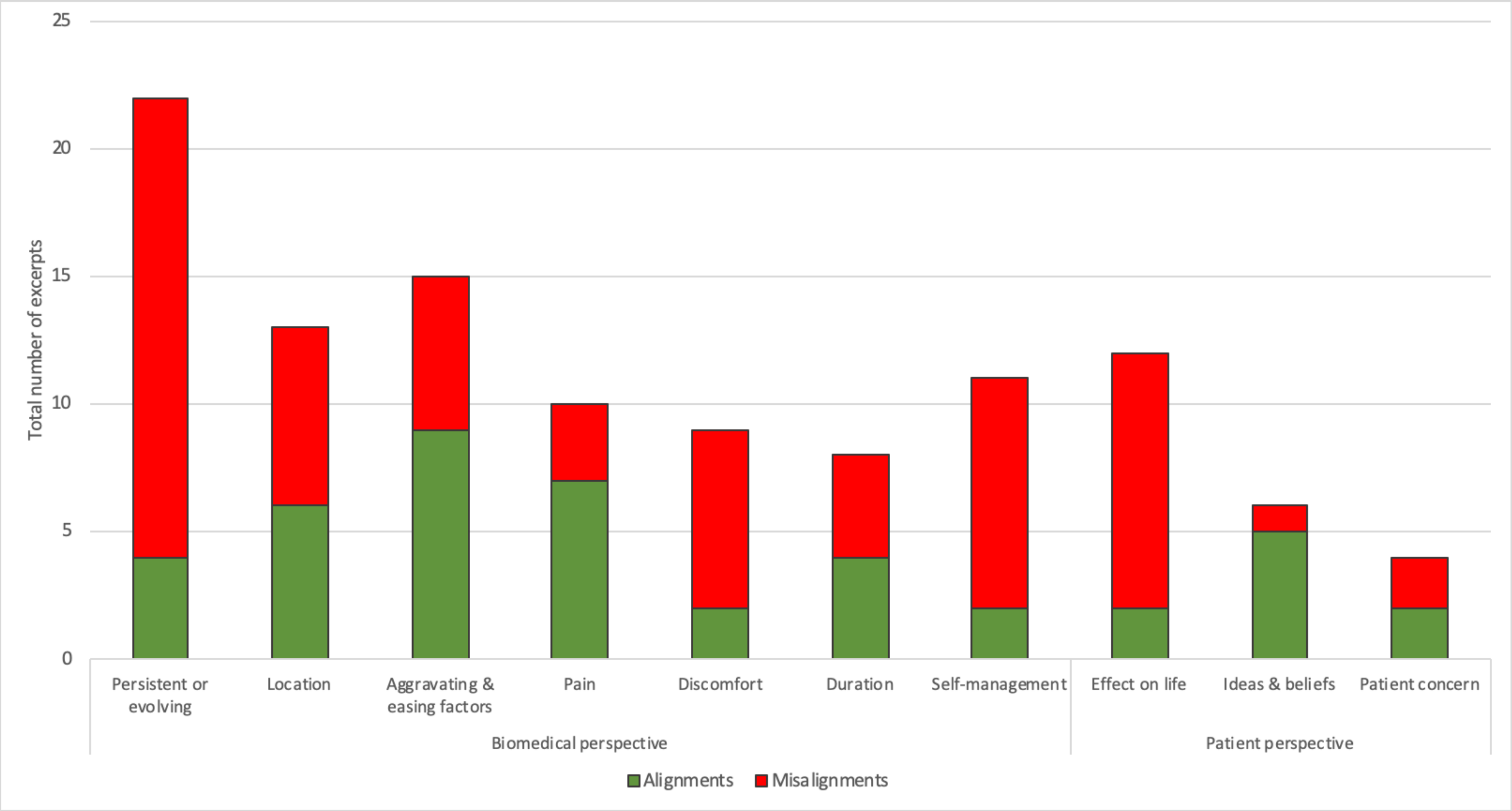

Supplementary file A. Details of included patients and their upper GI/systemic bodily sensations

Patient ID	Gender	Age	Bodily sensations
1	Female	66	Follow-up post-emergency operation for strangulated hernia, 'sticky bowels'
2	Female	71	Nausea, acid reflux
3	Female	72	Abdominal and pelvic pain, back ache
4	Male	55	Abdominal pain
5	Female	65	Nausea
6	Female	79	Heartburn, nausea
7	Female	73	Abdominal pain
8	Male	65	Abdominal and rectal pain, loose stools
9	Male	60	Acid reflux, indigestion
10	Male	59	Tiredness, headaches
11	Female	69	Abdominal distension/bloated
12	Male	73	Bowel pain, constipation
13	Male	83	Swallowing difficulties
14	Female	58	Back pain
15	Female	45	Fatigue, difficulty urinating
16	Male	81	Constipation
17	Female	77	Epigastric and back pain, nausea and vomiting
18	Female	53	Abdominal pain, nausea, diarrhoea, blood in stools
19	Female	42	Heartburn
20	Female	72	Reflux
21	Male	71	Loose stools, colitis flare-up
22	Female	41	Change in bowels, low haemoglobin
23	Female	70	Abdominal pain, change in bowels
24	Male	43	Fatigue, change in bowels
25	Female	59	Voice hoarseness, stomach problems
26	Male	74	Hernia
27	Male	64	Gastritis, follow-up of biopsy reversal
28	Female	53	Epigastric discomfort

For peer review only

COREQ (COnsolidated criteria for REporting Qualitative research) Checklist

A checklist of items that should be included in reports of qualitative research. You must report the page number in your manuscript where you consider each of the items listed in this checklist. If you have not included this information, either revise your manuscript accordingly before submitting or note N/A.

Topic	Item No.	Guide Questions/Description	Reported on Page No.
**Domain 1: Research team and reflexivity**			
Personal characteristics			
Interviewer/facilitator	1	Which author/s conducted the interview or focus group?	N/A
Credentials	2	What were the researcher's credentials? E.g. PhD, MD	15-16
Occupation	3	What was their occupation at the time of the study?	15-16
Gender	4	Was the researcher male or female?	N/A
Experience and training	5	What experience or training did the researcher have?	16
Relationship with participants			
Relationship established	6	Was a relationship established prior to study commencement?	N/A
Participant knowledge of the interviewer	7	What did the participants know about the researcher? e.g. personal goals, reasons for doing the research	N/A
Interviewer characteristics	8	What characteristics were reported about the inter viewer/facilitator? e.g. Bias, assumptions, reasons and interests in the research topic	N/A
**Domain 2: Study design**			
Theoretical framework			
Methodological orientation and Theory	9	What methodological orientation was stated to underpin the study? e.g. grounded theory, discourse analysis, ethnography, phenomenology, content analysis	2, 5
Participant selection			
Sampling	10	How were participants selected? e.g. purposive, convenience, consecutive, snowball	6
Method of approach	11	How were participants approached? e.g. face-to-face, telephone, mail, email	5, 6
Sample size	12	How many participants were in the study?	2, 7, 8
Non-participation	13	How many people refused to participate or dropped out? Reasons?	N/A
Setting			
Setting of data collection	14	Where was the data collected? e.g. home, clinic, workplace	5
Presence of non-participants	15	Was anyone else present besides the participants and researchers?	5
Description of sample	16	What are the important characteristics of the sample? e.g. demographic data, date	8
Data collection			
Interview guide	17	Were questions, prompts, guides provided by the authors? Was it pilot tested?	N/A
Repeat interviews	18	Were repeat inter views carried out? If yes, how many?	N/A
Audio/visual recording	19	Did the research use audio or visual recording to collect the data?	2, 5
Field notes	20	Were field notes made during and/or after the inter view or focus group?	6, 7
Duration	21	What was the duration of the inter views or focus group?	N/A
Data saturation	22	Was data saturation discussed?	N/A
Transcripts returned	23	Were transcripts returned to participants for comment and/or	N/A

For peer review only

For peer review only - http://bmjopen.bmj.com/site/about/guidelines.xhtml

Topic	Item No.	Guide Questions/Description	Reported on Page No.
		correction?	
**Domain 3: analysis and findings**			
Data analysis			
Number of data coders	24	How many data coders coded the data?	6-7
Description of the coding tree	25	Did authors provide a description of the coding tree?	6-7
Derivation of themes	26	Were themes identified in advance or derived from the data?	6-7
Software	27	What software, if applicable, was used to manage the data?	6-7
Participant checking	28	Did participants provide feedback on the findings?	15
Reporting			
Quotations presented	29	Were participant quotations presented to illustrate the themes/findings? Was each quotation identified? e.g. participant number	11-14
Data and findings consistent	30	Was there consistency between the data presented and the findings?	11-14
Clarity of major themes	31	Were major themes clearly presented in the findings?	11-14
Clarity of minor themes	32	Is there a description of diverse cases or discussion of minor themes?	11-14

Developed from: Tong A, Sainsbury P, Craig J. Consolidated criteria for reporting qualitative research (COREQ): a 32-item checklist for interviews and focus groups. *International Journal for Quality in Health Care*. 2007. Volume 19, Number 6: pp. 349 – 357

Once you have completed this checklist, please save a copy and upload it as part of your submission. DO NOT include this checklist as part of the main manuscript document. It must be uploaded as a separate file.

For peer review only
